# Experimental and Computational Investigations of the Reactions between α,β‐Unsaturated Lactones and 1,3‐Dienes by Cooperative Lewis Acid/Brønsted Acid Catalysis

**DOI:** 10.1002/anie.202008365

**Published:** 2020-08-18

**Authors:** Anja Weber, Martin Breugst, Jörg Pietruszka

**Affiliations:** ^1^ Institut für Bioorganische Chemie Heinrich-Heine-Universität Düsseldorf im Forschungszentrum Jülich Stetternicher Forst, Geb. 15.8 52426 Jülich Germany; ^2^ Department für Chemie Universität zu Köln Greinstraße 4 50939 Köln Germany; ^3^ Institut für Bio- und Geowissenschaften: Biotechnologie (IBG-1) Forschungszentrum Jülich GmbH 52428 Jülich Germany

**Keywords:** catalysis, computational chemistry, density functional calculations, Michael additions, natural products

## Abstract

The reactions of α,β‐unsaturated δ‐lactones with activated dienes such as 1,3‐dimethoxy‐1‐[(trimethylsilyl)oxy]‐1,3‐butadiene (Brassard's diene) are barely known in literature and show high potential for the synthesis of isocoumarin moieties. An in‐depth investigation of this reaction proved a stepwise mechanism via the vinylogous Michael‐products. Subsequent cyclisation and oxidation by LHMDS and DDQ, respectively, provided six mellein derivatives (30–84 %) and four angelicoin derivatives (40–78 %) over three steps. DFT‐calculations provide insights into the reaction mechanism and support the theory of a stepwise reaction.

## Graphical abstract

The reaction between α,β‐unsaturated lactones and 1,3‐dienes looks like a Diels–Alder reaction, but it is a vinylogous Michael addition as indicated by experimental results and DFT calculations. A short sequence was established accessing (natural) mellein and angelicoin derivatives.

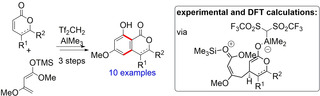

## Introduction

**Figure 1:**
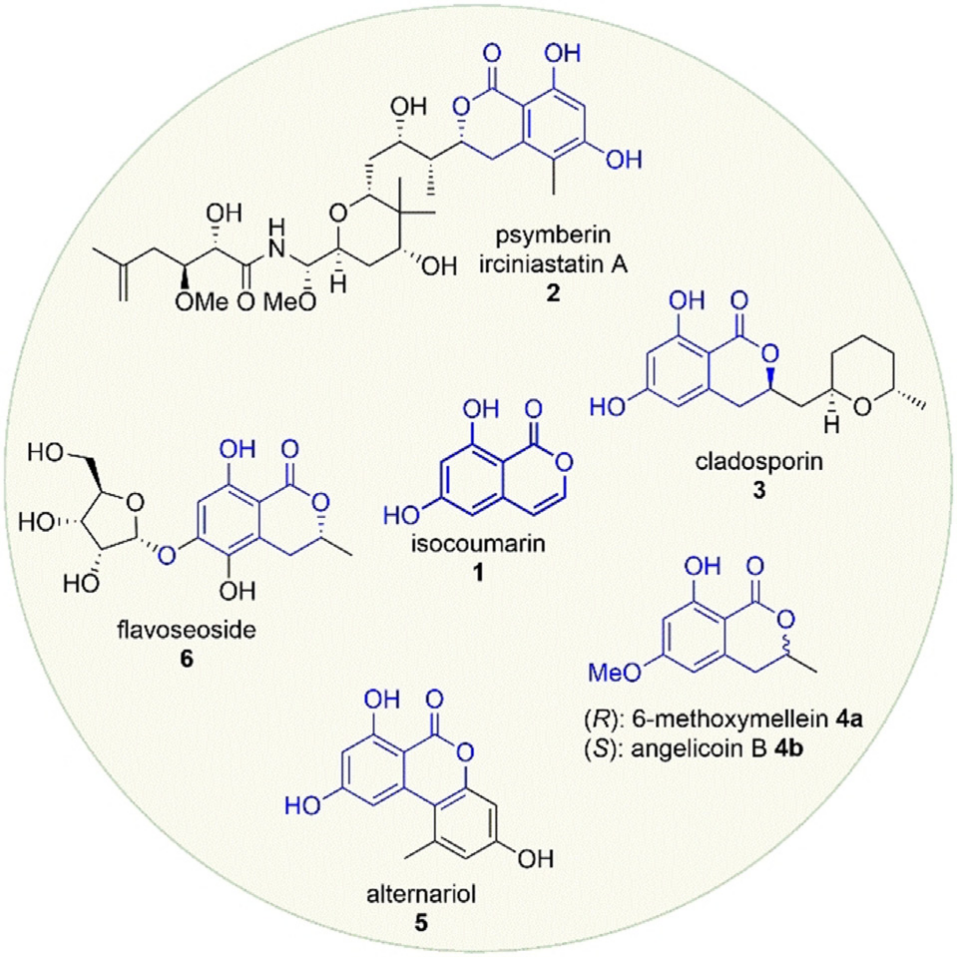
Selected structures of natural products containing an isocoumarin moiety (blue).

Isocoumarins **1**–**6**, *δ*‐valerolactones with a fused 1,3‐dihydroxybenzene, are prominent structural moieties in natural products. One example containing an isocoumarin moiety, is the marine natural product psymberin (**2**), also named irciniastatin A (**2**) (Figure 1).^1^, ^2^, ^3^, ^4^, ^5^, ^6^, ^7^, ^8^, ^9^ They were isolated independently from marine sponges *Psammocinia sp*. and *Ircinia ramose* by the two groups of Crews^7^ and Pettit in 2004.^8^ The structures were elucidated and claimed to be diastereoisomers, which was revised later by the first total synthesis and stereochemical assignment by de Barbander and co‐workers.^3^ Psymberin (**2**) has been tested on 60 different human cancer cell lines according to the NCI developmental therapeutics in vitro screening program. It showed promising cytotoxicity against melanoma, breast, and colon cancer cell lines (LC_50_<2.5×10^−9^ m).^7^ Cladosporin (**3**) is another mentionable natural product, which was first isolated from fungi *Cladosporium cladosporioides* by van Waalbeek and co‐workers in 1971 (Figure 1).^10^ The first asymmetric total synthesis was published by the group of She.^11^ Compound **3** shows promising antimalarial activity.^12^, ^13^

Mellein (**4 a**) is one of the simplest isocoumarin containing natural products.^14^ Mellein (**4 a**) and its derivatives have shown a wide biological activity range from antibacterial, antifungal to HCV‐protease inhibitory effects.^15^, ^16^, ^17^, ^18^ The (*S*)‐configured enantiomer is named angelicoin B (**4 b**).^19^, ^20^ The tricyclic alternariol (**5**) is a mycotoxin, which leads to crop loss.^21^ Alternariol (**5**) and its derivatives show high activity against bacteria, fungi, and cytotoxicity on human cancer cell lines.^22^ The Podlech group synthesised alternariol (**5**) in seven steps from orcinol and 3,5‐dimethoxybromobenzene in 2005, probably providing the best access to this compound today.^23^ One of the most recently isolated natural compounds with an isocoumarin moiety is flavoseoside (**6**) from *Malbranchea flavorose*. The structure could be elucidated in 2017.^24^

All these examples show that there is a need for a short and efficient synthesis of isocoumarins **1**–**6** and their derivatives as building blocks for natural product synthesis. For the increasing necessity of new drugs, caused by the growing population, resistances, and new diseases, we need to understand the mode of action of drugs, but also the chemical reactions. Therefore, it is a major challenge to develop general and predictable methods to achieve building block syntheses such as isocoumarins in an applicable way.

Currently, only few methods are established for the reaction of α,β‐unsaturated lactones with unsubstituted 1,3‐dienes,^25^, ^26^ alkynes,^27^ and bis(SiEt_3_)‐substituted dienes^28^ towards bicyclic isochromenones, whereupon none leads to 6,8‐dihydroxyisochroman‐1‐ones. The reported methods describe a concerted Diels–Alder type reaction between α,β‐unsaturated δ‐lactones with different types of dienes. The Diels–Alder [4+2]‐cycloaddition reaction is a powerful tool for the formation of C−C bonds and the synthesis of six‐membered rings. Accordingly, it is widely used in natural product synthesis and for industrial application.^29^, ^30^ Here we report a short synthesis of isocoumarins from α,β‐unsaturated lactones with 1,3‐dienes also providing some theoretical insight into the reaction mechanism. DFT calculations were used to describe the reaction mechanism of 5,6‐dihydro‐2*H*‐pyran‐2‐one (**7**) with (*Z*)‐[(1,3‐dimethoxybuta‐1,3‐dien‐1‐yl)oxy]trimethylsilane (Brassard's diene **8**).^31^ The combination of experimental and theoretical investigations then allowed us to propose a reaction mechanism for the isocoumarin formation.

## Results and Discussion

**Scheme 1:**
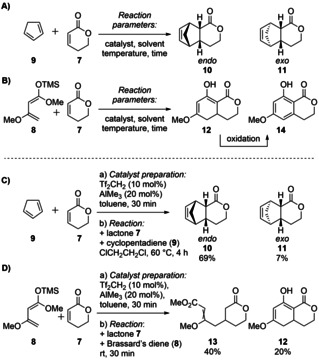
A) Diels–Alder product of α,β‐unsaturated δ‐lactone **7** and cyclopentadiene (**9**) as positive control. B) Initial approach towards Diels–Alder product after desilylation and subsequent oxidation. C) Reaction conditions for Diels–Alder reaction between α,β‐unsaturated δ‐lactone **7** and cyclopentadiene (**9**). D) Reaction conditions for reaction between α,β‐unsaturated δ‐lactone **7** and Brassard's diene (**8**).

*Initial Screening*. Initially, it was anticipated to perform a *Diels–Alder* reaction between an α,β‐unsaturated δ‐lactone and activated dienes such as Brassard's diene (**8**), thus obtaining isocoumarins as natural product building blocks after oxidation (Scheme 1). The initial reactions were examined with commercially available 5,6‐dihydro‐2*H*‐pyran‐2‐one (**7**) as a model dienophile and freshly distilled cyclopentadiene (**9**) (Scheme 1 A) or freshly prepared Brassard's diene (**8**) (Scheme 1 B). The reaction of 5,6‐dihydro‐2*H*‐pyran‐2‐one (**7**) with cyclopentadiene (**9**), was already known and used as a positive control for the tested reaction parameters.^26^, ^32^, ^33^

After an extensive catalyst [Lewis acids: AlBr_3_, AlMe_3_, AlCl_3_, Sc(OTf)_3_, Yb(OTf)_3_, Sm(OTf)_3_, ZnCl_2_, ZnI_2_, ZnBr_2_, EtAlCl_2_; Brønsted acids: Tf_2_CH_2_, Tf_2_NH], solvent (toluene, dichloromethane, acetonitrile, dichloroethane, *n*‐pentane), and temperature screening (−20 °C up to 100 °C), we found that the best working catalyst system was a combined system, using AlMe_3_ as Lewis acid and Tf_2_CH_2_ as Brønsted acid.^33^ Under all tested conditions without catalyst the reaction did not occur. Utilizing cyclopentadiene (**9**) as diene at 60 °C, 69 % *endo*‐product **10** and 7 % *exo*‐product **11** were obtained after 4 h when using 20 mol % AlMe_3_ and 10 mol % Tf_2_CH_2_ (Scheme 1, C). The same catalyst loadings also gave the desired 8‐hydroxy‐6‐methoxy‐3,4,4a,5‐tetrahydro‐1*H*‐iso‐chromen‐1‐one (**12**) when 5,6‐dihydro‐2*H*‐pyran‐2‐one (**7**) and freshly prepared Brassard's diene (**8**) were used. However, the conditions needed to be adapted because of the low stability of the diene at elevated temperature. Furthermore, low yields of about 10 % were observed after one‐hour reaction time in dichloroethane at room temperature. TLC and NMR analysis suggested full conversion of α,β‐unsaturated δ‐lactone **7** and Brassard's diene **8**, but indicated the formation of various side products. Changing the solvent to toluene and decreasing the reaction time to 30 minutes doubled the yield of the product to 20 %, but also gave the vinylogous Michael addition product methyl 3‐methoxy‐4‐(2‐oxotetrahydro‐2*H*‐pyran‐4‐yl)but‐2‐enoate (**13**) as the major product with 40 % yield (Scheme 1 D). Later this product was identified as the (*E*)‐configured product by nOe experiments.

**Scheme 2:**
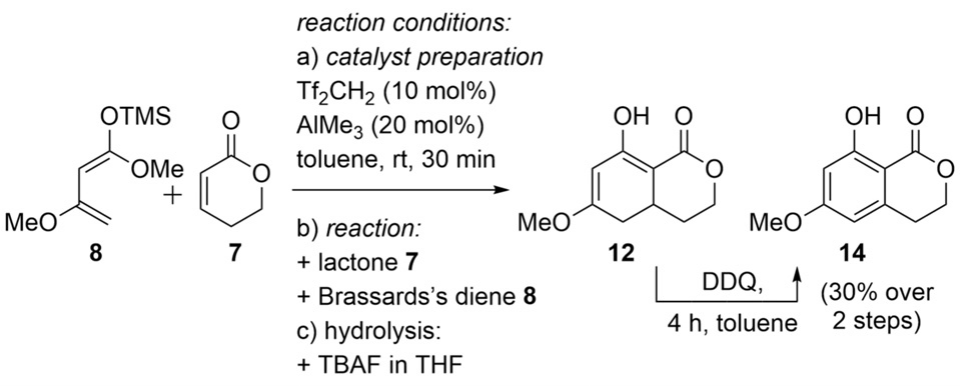
Diels–Alder type reaction of Brassard's diene (**8**) and 5,6‐dihydro‐2*H*‐pyran‐2‐one (**7**).

*Improving the Sequence*. Obviously, the low conversion to diene **12** led to low yields of the desired product **14**. Different catalyst ratios (Tf_2_CH_2_:AlMe_3_=1:1, 1:2, 1:3) were tested with 10 mol % Tf_2_CH_2_ and 20 mol % AlMe_3_ showing the best results. Furthermore, it was observed that the workup conditions were strongly influencing the yield. Two main aspects were considered for optimization, a) hydrolysis of the silyl ketene acetal and b) oxidation towards the aromatic product (Scheme 2; for details see Supporting Information). 2,3‐Dichloro‐5,6‐dicyano‐1,4‐benzoquinone (DDQ) (1.3 equiv, 4 h, quant.) gave the best results providing the desired isocoumarin **14** in quantitative yield from 8‐hydroxy‐6‐methoxy‐3,4,4a,5‐tetrahydro‐1*H*‐isochromen‐1‐one (**12**), and hence we exclusively used DDQ for further oxidations. Next, various conditions were tested for the hydrolysis of the silyl derivatives leading first to 6‐hydroxy‐8‐methoxy‐3,4,4a,5‐tetrahydro‐1*H*‐isochromen‐1‐one (**12**) and after oxidation to 8‐hydroxy‐6‐methoxy‐3,4,4a,5‐tetrahydro‐1*H*‐isochromen‐1‐one (**14**). Starting from the initial result using tetra‐*n*‐butylammonium fluoride (TBAF)^34^ in THF and a yield of 20 % over two steps, we tested alternative reagents. However, when directly oxidizing the crude mixture with DDQ without isolation of intermediate **12**, this setup proved superior and isochromen‐1‐one **14** was isolated in moderate 30 % yield.

**Scheme 3:**
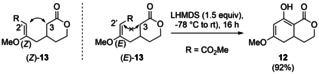
Proton proximity as detected by the nOe coupling between 3‐H_a_ and 2′‐H for (*Z*)‐**13** (left). Dieckmann reaction vinylogous (*E*)‐Michael‐product (*E*)‐**13** (right).

Until now, the yield could be increased from 10 % to 30 % of the desired 8‐hydroxy‐6‐methoxyisochroman‐1‐one (**14**), detecting full consumption of the α,β‐unsaturated δ‐lactone **7** by proton NMR and isolating the vinylogous Michael‐product **13** as the major product in 40 % yield. Measuring the proton NMR kinetic (see Supporting Information for details) at room temperature in [D_8_]toluene led to the formation of two products. They could be identified after isolation as the presumed vinylogous Michael addition products methyl (*Z*)‐3‐methoxy‐4‐(2‐oxotetrahydro‐2*H*‐pyran‐4‐yl)but‐2‐enoate [(*Z*)‐**13**] and methyl (*E*)‐3‐methoxy‐4‐(2‐oxotetrahydro‐2*H*‐pyran‐4‐yl)but‐2‐enoate [(*E*)‐**13**]. The assignment was based on the observed nOe between 3‐H_a_ and 2′‐H for the minor component (*Z*)‐**13** (25 %) and its absence in the major product (*E*)‐**13** (68 %, Scheme 3). To conclude, the NMR kinetics showed that the starting materials and intermediates **13** are stable under the reaction conditions, thus establishing a competing vinylogous Michael addition. This was confirmed by the fact that the major isomer [(*E*)‐**13**] could be deprotonated at the α‐carbon of the lactone, subsequently leading to the desired 6‐hydroxy‐8‐methoxy‐3,4,4a,5‐tetrahydro‐1*H*‐isochromen‐1‐one (**12**) via a Dieckmann condensation. While lithium diisopropylamide (LDA)^35^ gave only low yield, best results (for details see supporting information) were obtained with lithium bis(trimethylsilyl)amide (LHMDS)^36^, ^37^ (92 % yield).

**Scheme 4:**
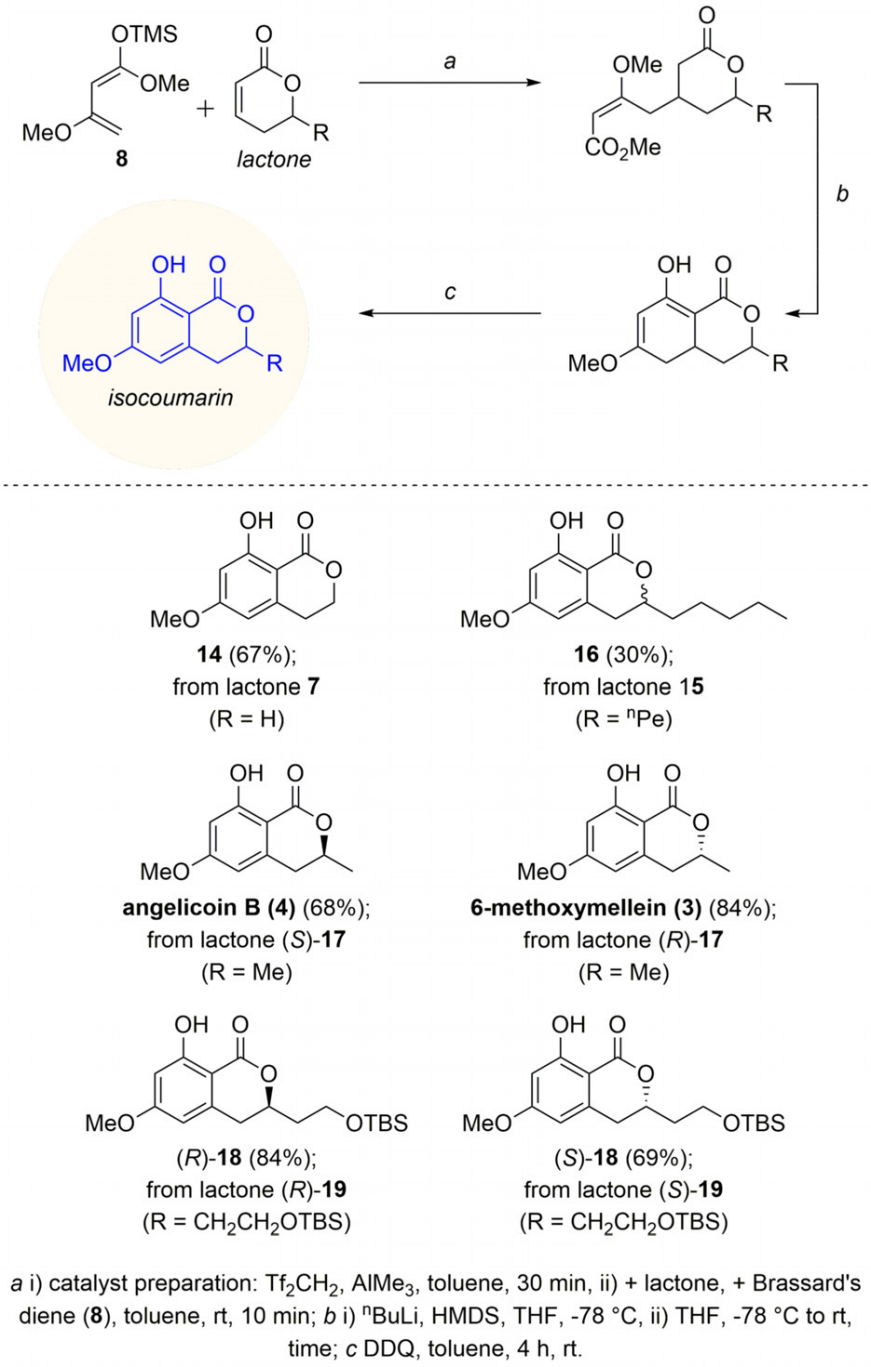
Synthesis of isocoumarins from *δ*‐substituted α,β‐unsaturated *δ*‐lactone.

*Substrate scope*. After optimization of the catalytic system, the work‐up conditions, the Dieckmann reaction as well as the final oxidation, the three‐step sequence was combined: The improved protocol led first to a separable mixture of 6‐hydroxy‐8‐methoxy‐3,4,4a,5‐tetrahydro‐1*H*‐isochromen‐1‐one (**12**) (30 %) together with (*E*)‐**13** (40 %). In a consecutive step, the (*E*)‐Michael‐product (*E*)‐**13** could be easily converted to the corresponding 6‐hydroxy‐8‐methoxy‐3,4,4a,5‐tetrahydro‐1*H*‐isochromen‐1‐one (**12**) with LHMDS (92 %). Finally, both fractions **12** could be oxidized with DDQ to the desired 8‐hydroxy‐6‐methoxyisochroman‐1‐one (**14**) in quantitative yield. Over three steps, the isocoumarin **14** was obtained in 67 % yield (Scheme 4).

Having established a convenient three‐step reaction sequence, the substrate scope was extended to lactones bearing substituents in δ‐position. Enantiomerically pure α,β‐unsaturated δ‐lactones were synthesized via a chemoenzymatic route,^38^, ^39^ utilizing α‐substituted allylboronic esters,^40^, ^41^ or were commercially available. The yields range from 30 % (R=^*n*^Pe: **15** to **16**) to 84 % [R=Me: (*R*)‐**17** to 6‐methoxymellein (**3**)]. Besides natural products such as angelicoin B (**4**) [68 % from (*S*)‐**17**], potential intermediates bearing a common protecting group (TBS: ^t^BuMe_2_Si) are also readily available (isocoumarin **18** from the corresponding lactone **19**). As expected, no racemization was observed as proven by HPLC analysis of lactone and isocoumarin product.

**Scheme 5:**
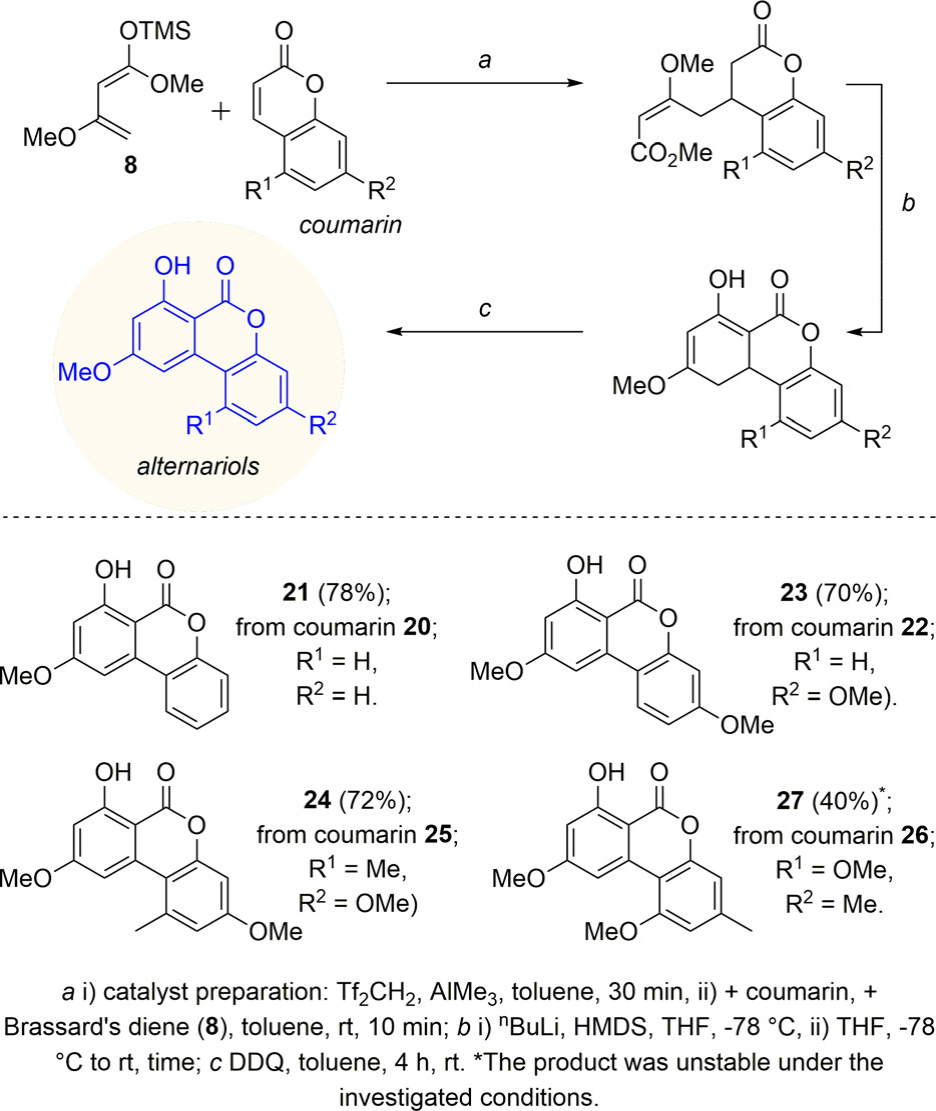
Synthesis of alternariol derivatives from coumarins.

Next, an expansion of the substrate scope from δ‐substituted lactones to coumarins was desirable, generating tricyclic, alternariol‐like products. Initially, the reaction with commercially available coumarin **20** (2*H*‐chromen‐2‐one) and Brassard's diene **8** was tested obtaining good yields (78 %) of product **21** over three steps (Scheme 5). Unprotected umbelliferon derivatives did not react. However, utilizing 3‐methoxy‐umbelliferon (**22**)^42^ the reaction worked well (70 % of product **23**). 3,9‐Dimethoxy alternariol (**24**) could also be synthesized in good yields of 72 %. The precursor, 7‐methoxy‐5‐methyl‐2*H*‐chromen‐2‐one (**25**), has been synthesized in a Pechmann reaction of 5‐methylresorcin with propiolic acid, catalysed by ytterbium(III) trifluoromethanesulfonate^43^ and direct protection of the hydroxyl‐group with dimethyl sulfate. The reaction gave the constitutional isomers 5‐methoxy‐7‐methyl‐2*H*‐chromen‐2‐one (**26**) and 7‐methoxy‐5‐methyl‐2*H*‐chromen‐2‐one (**25**) in a 1:1‐ratio. The constitutional isomer 5‐methoxy‐7‐methyl‐2*H*‐chromen‐2‐one (**26**) gave the unstable alternariol derivative **27** in moderate yield (40 %) over three steps.

*Summary Synthetic Investigation*. After extensive catalyst screening, a cooperative catalyst system of AlMe_3_ and Tf_2_CH_2_ was found as the best working system for the investigated reaction. Getting the vinylogous Michael‐product as the major product and determining its (*E*)‐configuration by nOe measurements, the idea of direct cyclisation of this (*E*)‐configured Michael‐product towards the desired isocoumarin was established. The crude product could readily be oxidised by DDQ to the desired isocoumarins. Overall, six different mellein derivatives and four different angelicoin derivatives could be synthesized in moderate to good yields over three steps (30–84 %).

**Scheme 6:**
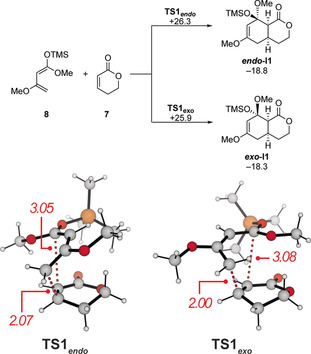
Calculated Gibbs free energies (in kcal mol^−1^) for the uncatalyzed cycloaddition between **7** and **8** (above) and structure as well as selected bond lengths (in Å) for the transition states **TS1** (below).

*Computational investigation*. In order to receive more detailed information on the reaction mechanism and to understand as well as explain the unexpected high reactivity of the catalytic system Tf_2_CH_2_/AlMe_3_ in the reactions between α,β‐unsaturated lactones and 1,3‐dienes, next we carefully analysed the reaction between lactone **7** and Brassard's diene (**8**) computationally [M06‐2X‐D3/def2‐QZVP/IEFPCM(toluene)//M06‐L‐D3/6–31+G(d,p)/IEFPCM(toluene)].^44^ In the absence of any catalyst, the reaction between **7** and **8** is thermodynamically favourable to yield ***endo*** **‐** and ***exo*** **‐I1** (Δ*G*=−18.8 and −18.3 kcal mol^−1^). The cycloaddition proceeds through transition states **TS1**_***endo***_ and **TS1**_***exo***_ of very similar energies (Δ*G* ^≠^=26.3 and 25.9 kcal mol^−1^). The forming C−C bond lengths significantly differ (2.07 and 2.00 vs. 3.05 and 3.08 Å, Scheme 6) within **TS1** but no zwitterionic intermediates could be identified when following the intrinsic reaction coordinate (IRC) path. In line with this, most zwitterionic structures collapsed to the cycloadducts in separate calculations and stable structures were found to be significantly higher in energy (>33 kcal mol^−1^). Therefore, it can be concluded that a putative background reaction should proceed through a concerted, yet asynchronous reaction. The high barrier of ca. 26 kcal mol^−1^ is qualitatively in line with the experimental finding that no cycloaddition product could be detected even upon heating to 100 °C. Instead, a decomposition of Brassard's diene (**8**) was observed at elevated temperatures.

**Scheme 7:**
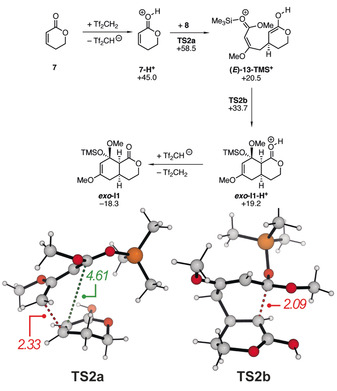
Calculated Gibbs free energies (in kcal mol^−1^) for the Brønsted acid catalyzed cycloaddition between **7** and **8** (above) and structure as well as selected bond lengths (in Å) for the transition states **TS2** (below).

Different mechanistic proposals can be suggested for the Tf_2_CH_2_/AlMe_3_‐catalyzed reaction: Brønsted acid catalysis by Tf_2_CH_2_ or a more acidic Tf_2_CH_2_‐AlMe_3_ adduct, Lewis acid catalysis by AlMe_3_, or alternative catalytic species formed in the reaction must be considered. We first investigated a Brønsted acid catalysis pathway as summarized in Scheme 7, with selected structures shown. This reaction starts with the protonation of the lactone by Tf_2_CH_2_ (values for other Brønsted acids like TfOH or HCl are shown in the Supporting Information. According to our calculations, this process is highly endergonic (+45 kcal mol^−1^). The value is probably overestimated due to the unfavourable charge separation in the calculations and additional specific solvent‐solute interactions not taken into account in continuum models. All attempts to locate concerted pathways failed in these cases and all transition‐state guesses resulted in step‐wise mechanisms with the formation of a zwitterionic intermediate. The most M06‐2X‐D3/def2‐QZVP/IEFPCM(toluene)//M06‐L‐ transition state for the Brønsted acid catalysis **TS2 a** requires an activation free energy of 58.5 kcal mol^−1^ and results in the unstable zwitterion **(*E*)‐13‐TMS^+^**. In this transition state, the length of the forming C−C bond is 2.33 Å, while the second set of carbon atoms is still well separated (4.61 Å). Interestingly, transition states leading to a *Z*‐configured double bond within **13‐TMS^+^** are significantly lower in energy (Δ*G* ^≠^=+46.1 kcal mol^−1^, not shown in Scheme 7). However, these structures are unproductive as they cannot react further to yield the cycloadduct, as observed and described experimentally. The zwitterion **(*E*)‐13‐TMS^+^** then collapses via a small barrier (**TS2 b**, Δ*G* ^≠^=+48.0 kcal mol^−1^) to give the protonated cycloadduct ***exo*** **‐I1‐H^+^**. In line with experimental results, the calculations (even though the barriers might be overestimated) clearly demonstrate that a simple Brønsted acid catalysis is not likely to be the origin of the high activities.

**Scheme 8:**
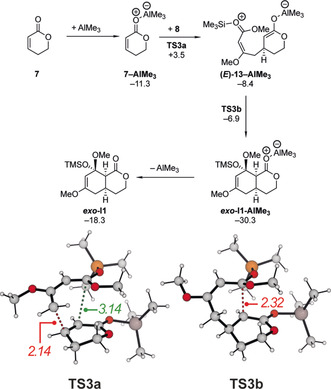
Calculated Gibbs free energies (in kcal mol^−1^) for the AlMe_3_‐catalyzed cycloaddition between **7** and **8** (above) and structure as well as selected bond lengths (in Å) for the transition states **TS3** (below).

We next focused our attention on the potential Lewis acid catalysis by AlMe_3_ as catalyst (Scheme 8). Again, the first step is the activation of the lactone through coordination to the Lewis acid. Based on our calculations this is a favourable process (Δ*G*=−11.3 kcal mol^−1^) that should occur readily. Again, all identified transition state structures resulted in stepwise reactions as discussed for the Brønsted acid catalysis above. The formation of the first C−C bond proceeds with an activation free energy of 14.8 kcal mol^−1^ through **TS3 a** and results in the zwitterionic intermediate **(*E*)‐13‐AlMe_3_**. Similar to the Brønsted acid catalysis described above, the formation of a *Z*‐configured intermediate is also possible for AlMe_3_ and proceeds with a comparable barrier. The zwitterion collapses in the next step without significant barrier via **TS3 b** (Δ*G* ^≠^=+1.5 kcal mol^−1^) with only a very small barrier. Based on the computed activation free energy of 14.8 kcal mol^−1^, a reaction should be observable in the presence of catalytic amounts of AlMe_3_, although no product formation was detected experimentally under the screening conditions. As other functionals (DSD‐BLYP‐D3BJ, *ω*B97X‐D, B3LYP‐D3BJ) resulted in similar barriers around 15 kcal mol^−1^, we can exclude a systematic error in the M06‐2X calculations. Similarly, our calculations further indicate that the interaction between AlMe_3_ and with the lactone (Δ*G*=−11.3 kcal mol^−1^) is slightly stronger than an interaction with any of the three oxygen atoms of Brassard's diene (−7.5<Δ*G*<−4.1 kcal mol^−1^). Therefore, we have to conclude that either the solvent, which is present in a large excess, or the product interacts with the Lewis acid and lowers the reactivity and in turn increase the activation free energy.

**Scheme 9:**
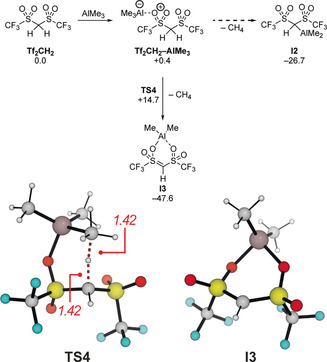
Formation of the catalytically active species between Tf_2_CH_2_ and AlMe_3_ (free energies in kcal mol^−1^) (above) and structures of the transition state **TS4** and the potential catalyst **I3** and selected bond lengths (in Å, below).

Finally, we addressed the full catalytic system consisting of Tf_2_CH_2_ and AlMe_3_. In previous investigations, Taguchi and co‐workers proposed that Tf_2_CH_2_ reacts with AlMe_3_ to form the aluminum methide **I2** and methane. **I2** could then either react as a stronger Brønsted or Lewis acid to catalyze the Diels–Alder reaction.^33^ Based on our calculations (Scheme 9), a Lewis acid‐base pair is formed first in a thermoneutral reaction. The proposed aluminum methide **I2** could then be formed in an exergonic reaction (Δ*G*=−27.3 kcal mol^−1^), but no transition states could be identified for this reaction. Instead, all transition states indicate that the isomeric O‐substituted species **I3** is formed instead. **I3** is not only considerably more stable than its isomer **I2**, but it is also formed through a small activation free energy of 14.4 kcal mol^−1^ (**TS4**). The dual coordination of the AlMe_2_‐fragment to both sulfonyl groups significantly contributes to the higher thermodynamic stability of **I3**. The up‐field shifts for the Tf_2_ *C*H‐carbon and Tf_2_C*H*‐proton reported by Taguchi and co‐workers are both in agreement with **I2** and the isomeric structure **I3**.

**Scheme 10:**
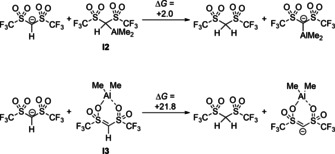
Calculated free energies for the isodesmic proton‐transfer reactions between **I2** and **I3** and the Tf_2_CH anion (in kcal mol^−1^).

Both **I2** and **I3** could now act either as Brønsted acids or Lewis acids to catalyze the subsequent [4+2] cycloaddition. For an estimate of the change in Brønsted acidity, we calculated the reaction free energies for the isodesmic proton‐transfer reactions as shown in Scheme 10. As these reactions are either almost thermoneutral (**I2**) or highly unfavourable (**I3**), one has to conclude that **I2** and **I3** cannot be considered as significantly stronger Brønsted acids compared to the free Tf_2_CH_2_. Consequently, a Brønsted acid catalysis is rather unlikely as the origin of the catalytic activity.

**Scheme 11:**
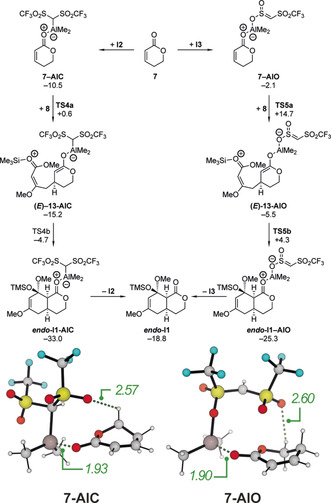
Calculated Gibbs free energies (in kcal mol^−1^) for the cycloaddition between **7** and **8** catalyzed by the Lewis acid **I2** or **I3** (above). Structure and selected bond lengths (in Å) for the complexes between **7** and **I2** or **I3**, respectively (below).

Therefore, we focused on the Lewis acid catalysis pathway and wondered how **I2** and **I3** activate lactone **7**. The interaction of **I2** with the lactone **7** leading to **7‐AlC** is again an exergonic reaction and comparable yet slightly weaker than that of AlMe_3_ (Scheme 11, left). Interestingly, the *Z*‐configuration is more stable compared to the *E*‐configuration, which might be attributed to the stronger C−H⋅⋅⋅O hydrogen bond (Scheme 11). However, the subsequent C−C bond formation occurs through **TS4 a** with an activation free energy of only 11.1 kcal mol^−1^. In contrast to the previous systems, the zwitterionic intermediate **(*E*)‐13‐AlC** next collapses through a comparable barrier of 10.5 kcal mol^−1^ to give the *endo* cycloadduct ***endo*** **‐I1‐AlC**. The higher stability of the zwitterionic intermediate with respect to the second bond formation also explains, why the Michael adduct **13** is observed as the main product in the absence of oxidants or fluoride salts (Scheme 1 d).

Alternatively, **I3** could be the active catalyst of the AlMe_3_‐Tf_2_CH_2_ mixture and the calculated Gibbs free energies are summarized in Scheme 11 (right). Based on our computations, the interaction of **I3** with the lactone **7** is considerably weaker (Δ*G*=−2.1 kcal mol^−1^) than that of **I2**. In contrast, the direct comparison of the isomeric adducts **7‐AlC** and **7‐AlO** (Scheme 11, below) reveals, that the latter is thermodynamically preferred over the former by 12.4 kcal mol^−1^ (not shown in Scheme 11). This can be attributed to the high intrinsic stability of the free Lewis acid **I3**. Interestingly, no additional hydrogen bonds (e.g. between the Tf_2_C‐H and the O‐atom of the ester) stabilize these complexes. The subsequent C−C bond formation proceeds via **TS5 a** with an activation barrier of 16.8 kcal mol^−1^, followed by the cyclization via **TS5 b** with an activation free energy of 9.8 kcal mol^−1^. Again, the second C−C bond formation occurs much faster than the first one, but as the barriers of both steps are closer in energy than e.g, in Scheme 8, the life time of the intermediate (*E*)‐**13‐AlO** should also be larger.

When comparing the different mechanistic pathways of Schemes 7, 8, 9, and 11, a Lewis acid catalysis by the AlMe_3_‐Tf_2_CH_2_‐mixture results in the lowest activation free energy. Among the different isomers of the catalyst, the computational data indicate that **I2** is most likely the catalytically active species. The calculated activation free energies for the Lewis acid catalyzed reactions are probably underestimated as both Lewis acids are likely to interact with the solvent molecules in the system. Given the similar interaction energies with carbonyl groups, it can be expected that the solvent‐solute interactions are also comparable in both cases. These findings are also in line with previous ^13^C NMR investigations by Taguchi and colleagues, as a stronger change in chemical shifts was observed for AlMe_3_‐Tf_2_CH_2_ than for AlMe_3_ alone.^26^ This indicates that coordination to the former results in a larger lowering of the LUMO of the Michael acceptor.

## Conclusion

Experimental and computational investigations show that the reaction of 5,6‐dihydro‐2*H*‐pyran‐2‐one (**7**) and (*Z*)‐((1,3‐dimethoxybuta‐1,3‐dien‐1‐yl)oxy)trimethylsilane (**8**) (Brassard's diene) catalyzed by AlMe_3_ and Tf_2_CH_2_ undergoes a stepwise mechanism and no concerted *Diels–Alder* like reaction, making a competing vinylogous Michael addition possible. The experiments show that the major Michael‐product is (*E*)‐configured **(*E*)‐13**, which could be verified by nOe‐spectra and the convenient conversion into the cyclized product **12** by LHMDS. Oxidation of the intermediates **12** with DDQ gives the aromatic isocoumarins **14**. Overall, six mellein derivatives and four angelicoin derivatives could be synthesized in moderate to good yields over three steps (30–84 %). The computational results underline the experimental results, showing the vinylogous Michael addition and the AlMe_3_/Tf_2_CH_2_‐system as catalyst to be energetically favoured, in comparison to the direct formation of the Diels–Alder product and single AlMe_3_ as catalyst.

## Conflict of interest

The authors declare no conflict of interest.

## Supporting information

As a service to our authors and readers, this journal provides supporting information supplied by the authors. Such materials are peer reviewed and may be re‐organized for online delivery, but are not copy‐edited or typeset. Technical support issues arising from supporting information (other than missing files) should be addressed to the authors.

Supplementary

## References

[1] Y. Feng , X. Jiang , J. K. De Brabander , J. Am. Chem. Soc. 2012, 134, 17083–17093. 23004238 10.1021/ja3057612 PMC3482988

[2] X. Huang , N. Shao , R. Huryk , A. Palani , R. Aslanian , C. Seidel-Dugan , Org. Lett. 2009, 11, 867–870. 19199777 10.1021/ol802772s

[3] X. Jiang , J. García-Fortanet , J. K. De Brabander , J. Am. Chem. Soc. 2005, 127, 11254–11255. 16089449 10.1021/ja0537068

[4] S. Wan , F. Wu , J. C. Rech , M. E. Green , R. Balachandran , W. S. Horne , B. W. Day , P. E. Floreancig , J. Am. Chem. Soc. 2011, 133, 16668–16679. 21902245 10.1021/ja207331m

[5] J. Yu , M. Yang , Y. Guo , T. Ye , Org. Lett. 2019, 21, 3670–3673. 31063392 10.1021/acs.orglett.9b01113

[6] M. Bielitza , J. Pietruszka , Angew. Chem. Int. Ed. 2013, 52, 10960–10985; 10.1002/anie.201301259 24105772

[7] R. H. Cichewicz , F. A. Valeriote , P. Crews , Org. Lett. 2004, 6, 1951–1954. 15176791 10.1021/ol049503q

[8] G. R. Pettit , J.-P. Xu , J.-C. Chapuis , R. K. Pettit , L. P. Tackett , D. L. Doubek , J. N. A. Hooper , J. M. Schmidt , J. Med. Chem. 2004, 47, 1149–1152. 14971894 10.1021/jm030207d

[9] Q. Liu , C. An , K. TenDyke , H. Cheng , Y. Y. Shen , A. T. Hoye , A. B. Smith , J. Org. Chem. 2016, 81, 1930–1942. 26879056 10.1021/acs.joc.5b02771 PMC4782725

[10] P. M. Scott , W. Van Walbeek , W. M. MacLean , J. Antibiot. 1971, 24, 747–755. 10.7164/antibiotics.24.747 5169000

[11] H. Zheng , C. Zhao , B. Fang , P. Jing , J. Yang , X. Xie , X. She , J. Org. Chem. 2012, 77, 5656–5663. 22663064 10.1021/jo300805n

[12] S. Khan , A. Sharma , H. Belrhali , M. Yogavel , A. Sharma , J. Struct. Funct. Genomics 2014, 15, 63–71. 24935905 10.1007/s10969-014-9182-1

[13] W. A. Guiguemde , R. K. Guy , Cell Host Microbe 2012, 11, 555–557. 22704614 10.1016/j.chom.2012.05.008

[14] H. Nishikawa , Bull. Agric. Chem. Soc. Jpn. 1933, 9, 107–109.

[15] C. Wohlfarth , T. Efferth , Acta Pharm. Sin. 2009, 30, 25–30. 10.1038/aps.2008.5 PMC4006527 19060918

[16] K. Krohn , R. Bahramsari , U. Flörke , K. Ludewig , C. Kliche-Spory , A. Michel , H.-J. Aust , S. Draeger , B. Schulz , S. Antus , Phytochemistry 1997, 45, 313–320. 9141717 10.1016/s0031-9422(96)00854-0

[17] U. Höller , G. M. König , A. D. Wright , J. Nat. Prod. 1999, 62, 114–118. 9917295 10.1021/np980341e

[18] F. Marinelli , U. Zanelli , V. N. Ronchi , Phytochemistry 1996, 42, 641–643.

[19] M. Shibano , H. Naito , M. Taniguchi , N.-H. Wang , K. Baba , Chem. Pharm. Bull. 2006, 54, 717–718. 10.1248/cpb.54.717 16651776

[20] K. Anderson , F. Calo , T. Pfaffeneder , A. J. P. White , A. G. M. Barrett , Org. Lett. 2011, 13, 5748–5750. 21936533 10.1021/ol202320m

[21] H. Raistrick , C. E. Stickings , R. Thomas , Biochem. J. 1953, 55, 421–433. 13105649 10.1042/bj0550421 PMC1269293

[22] D. Lai , A. Wang , Y. Cao , K. Zhou , Z. Mao , X. Dong , J. Tian , D. Xu , J. Dai , Y. Peng , L. Zhou , Y. Liu , J. Nat. Prod. 2016, 79, 2022–2031. 27441892 10.1021/acs.jnatprod.6b00327

[23] K. Koch , J. Podlech , E. Pfeiffer , M. Metzler , J. Org. Chem. 2005, 70, 3275–3276. 15822993 10.1021/jo050075r

[24] B. Verastegui-Omaña , D. Rebollar-Ramos , A. Pérez-Vásquez , A. L. Martínez , A. Madariaga-Mazón , L. Flores-Bocanegra , R. Mata , J. Nat. Prod. 2017, 80, 190–195. 28060505 10.1021/acs.jnatprod.6b00977

[25] Y. Zhang , Y. Tian , P. Xiang , N. Huang , J. Wang , J.-H. Xu , M. Zhang , Org. Biomol. Chem. 2016, 14, 9874–9882. 27722745 10.1039/c6ob01701k

[26] A. Saito , H. Yanai , T. Taguchi , Tetrahedron Lett. 2004, 45, 9439–9442.

[27] T. Sambaiah , L.-P. Li , D.-J. Huang , C.-H. Lin , D. K. Rayabarapu , C.-H. Cheng , J. Org. Chem. 1999, 64, 3663–3670. 11674495 10.1021/jo9900580

[28] Z. Liu , X. Lin , N. Yang , Z. Su , C. Hu , P. Xiao , Y. He , Z. Song , J. Am. Chem. Soc. 2016, 138, 1877–1883. 26799581 10.1021/jacs.5b09689

[29] K. C. Nicolaou , S. A. Snyder , T. Montagnon , G. Vassilikogiannakis , Angew. Chem. Int. Ed. 2002, 41, 1668–1698; 10.1002/1521-3773(20020517)41:10<1668::aid-anie1668>3.0.co;2-z 19750686

[30] J.-A. Funel , S. Abele , Angew. Chem. Int. Ed. 2013, 52, 3822–3863; 10.1002/anie.201201636 23447554

[31] J. Savard , P. Brassard , Tetrahedron Lett. 1979, 20, 4911–4914.

[32] T. Ikeda , S. Yue , C. R. Hutchinson , J. Org. Chem. 1985, 50, 5193–5199.

[33] H. Yanai , A. Takahashi , T. Taguchi , Tetrahedron 2007, 63, 12149–12159.

[34] M. Tamiya , K. Ohmori , M. Kitamura , H. Kato , T. Arai , M. Oorui , K. Suzuki , Chem. Eur. J. 2007, 13, 9791–9823. 17907132 10.1002/chem.200700863

[35] C. Kowalski , X. Creary , A. J. Rollin , M. C. Burke , J. Org. Chem. 1978, 43, 2601–2608.

[36] M. W. Rathke , J. Am. Chem. Soc. 1970, 92, 3222–3223.

[37] B. L. Lucht , D. B. Collum , J. Am. Chem. Soc. 1994, 116, 6009–6010.

[38] T. Fischer , J. Pietruszka , Adv. Synth. Catal. 2012, 354, 2521–2530.

[39] T. Fischer , J. Pietruszka , Adv. Synth. Catal. 2007, 349, 1533–1536.

[40] D. Böse , P. Niesobski , M. Lübcke , J. Pietruszka , J. Org. Chem. 2014, 79, 4699–4703. 24745807 10.1021/jo5004168

[41] S. Bartlett , D. Böse , D. Ghori , B. Mechsner , J. Pietruszka , Synthesis 2013, 45, 1106–1114.

[42] G. N. Vyas , N. M. Shah , Org. Synth. 1951, 31, 90.

[43] S. Fiorito , F. Epifano , V. A. Taddeo , S. Genovese , Tetrahedron Lett. 2016, 57, 2939–2942.

[44] See the Supporting Information for details of the employed computational methods.

