# Supplementary material for: Experimental and Computational Investigations of the Reactions between α,β‐Unsaturated Lactones and 1,3‐Dienes by Cooperative Lewis Acid/Brønsted Acid Catalysis
Source: Angew Chem Int Ed Engl. 2020 Aug 18;59(42):18709–16. doi: 10.1002/anie.202008365 (PMC7589441; doi:10.1002/anie.202008365)
Supplement: Supplementary file 1 — Supplementary [file ANIE-59-18709-s001.pdf]

Angewandte Chemie
Eine Zeitschrift der Gesellschaft Deutscher Chemiker

Supporting Information

Experimental and Computational Investigations of the Reactions between α,β-Unsaturated Lactones and 1,3-Dienes by Cooperative Lewis Acid/Brønsted Acid Catalysis

Anja Weber, Martin Breugst, and Jörg Pietruszka**

anie_202008365_sm_miscellaneous_information.pdf

Supporting Information

Table of Content

1. General Information S5

1.1 General Procedure for the catalyst preparation S5

1.2 General procedure for the deprotonation-cyclisation of the (*E*)-*Michael*-product S5

1.3 General procedure for the oxidation with DDQ S6

2. Optimisation Studies S7

2.1 Workup and Oxidation Condition Screening S7

2.2 Cyclisation of the Vinylogous Michael-Product S8

3. Products S9

8-Hydroxy-6-methoxy-3,4,4a,5-tetrahydro-1*H*-isochromen-1-one (12) S9

8-Hydroxy-6-methoxyisochroman-1-one (14) S9

Methyl (*E*)-3-methoxy-4-(2-oxotetrahydro-2*H*-pyran-4-yl)but-2-enoate [(*E*)-13] S10

Methyl (*Z*)-3-methoxy-4-(2-oxotetrahydro-2*H*-pyran-4-yl)but-2-enoate [(*Z*)-13] S10

(*R*)-8-Hydroxy-6-methoxy-3-methylisochroman-1-one [(*R*)-3] S11

Methyl (*E*)-3-methoxy-4-((2*R*)-2-methyl-6-oxotetrahydro-2*H*-pyran-4-yl)but-2-enoate S11

(*S*)-8-Hydroxy-6-methoxy-3-methylisochroman-1-one [(*S*)-4] S12

Methyl (*E*)-3-methoxy-4-((2*S*)-2-methyl-6-oxotetrahydro-2*H*-pyran-4-yl)but-2-enoate S12

8-Hydroxy-6-methoxy-3-pentylisochroman-1-one (16) S13

Methyl (*E*)-3-methoxy-4-(2-oxo-6-pentyltetrahydro-2*H*-pyran-4-yl)but-2-enoate S13

(*S*)-3-(2-((*tert*-Butyldimethylsilyl)oxy)ethyl)-8-hydroxy-6-methoxyisochroman-1-one [(*S*)-18] S14

Methyl (*E*)-4-((2*S*)-2-(2-((*tert*-butyldimethylsilyl)oxy)ethyl)-6-oxotetrahydro-2*H*-pyran-4-yl)-3-methoxybut-2-enoate S14

(*R*)-3-(2-((*tert*-Butyldimethylsilyl)oxy)ethyl)-8-hydroxy-6-methoxyisochroman-1-one [(*R*)-18] S15

Methyl (*E*)-4-((2*R*)-2-(2-((*tert*-butyldimethylsilyl)oxy)ethyl)-6-oxotetrahydro-2*H*-pyran-4-yl)-3-methoxybut-2-enoate S15

7-Hydroxy-9-methoxy-6*H*-benzo[*c*]chromen-6-one (21) S16

Methyl (*E*)-3-methoxy-4-(2-oxochroman-4-yl)but-2-enoate S17

7-Hydroxy-3,9-dimethoxy-6*H*-benzo[*c*]chromen-6-one (23) S17

Methyl (*E*)-3-methoxy-4-(7-methoxy-2-oxochroman-4-yl)but-2-enoate S18

7-Hydroxy-3,9-dimethoxy-1-methyl-6*H*-benzo[*c*]chromen-6-one (24) S18

Methyl (*E*)-3-methoxy-4-(7-methoxy-5-methyl-2-oxochroman-4-yl)but-2-enoate S19

7-Hydroxy-1,9-dimethoxy-3-methyl-6*H*-benzo[*c*]chromen-6-one (27) S19

Methyl (*E*)-3-methoxy-4-(5-methoxy-7-methyl-2-oxochroman-4-yl)but-2-enoate S20

4. NMR Kinetics S21

5. NMR Spectra S22

8-Hydroxy-6-methoxy-3,4,4a,5-tetrahydro-1*H*-isochromen-1-one (12) S22

8-Hydroxy-6-methoxyisochroman-1-one (14) S23

Methyl (*E*)-3-methoxy-4-(2-oxotetrahydro-2*H*-pyran-4-yl)but-2-enoate [(*E*)-13] S24

Methyl (*Z*)-3-methoxy-4-(2-oxotetrahydro-2*H*-pyran-4-yl)but-2-enoate [(*Z*)-13] S25

(*R*)-8-Hydroxy-6-methoxy-3-methylisochroman-1-one [(*R*)-3] S26

Methyl (*E*)-3-methoxy-4-((2*R*)-2-methyl-6-oxotetrahydro-2*H*-pyran-4-yl)but-2-enoate S27

(*S*)-8-Hydroxy-6-methoxy-3-methylisochroman-1-one [(*S*)-4] S28

Methyl (*E*)-3-methoxy-4-((2*S*)-2-methyl-6-oxotetrahydro-2*H*-pyran-4-yl)but-2-enoate S29

8-Hydroxy-6-methoxy-3-pentylisochroman-1-one (16) S30

Methyl (*E*)-3-methoxy-4-(2-oxo-6-pentyltetrahydro-2*H*-pyran-4-yl)but-2-enoate S31

(*S*)-3-(2-((*tert*-Butyldimethylsilyl)oxy)ethyl)-8-hydroxy-6-methoxyisochroman-1-one [(*S*)-18] S32

Methyl (*E*)-4-((2*S*)-2-(2-((*tert*-butyldimethylsilyl)oxy)ethyl)-6-oxotetrahydro-2*H*-pyran-4-yl)-3-methoxybut-2-enoate S33

(*R*)-3-(2-((*tert*-butyldimethylsilyl)oxy)ethyl)-8-hydroxy-6-methoxyisochroman-1-one [(*R*)-18] S34

Methyl (*E*)-4-((2*R*)-2-(2-((*tert*-butyldimethylsilyl)oxy)ethyl)-6-oxotetrahydro-2*H*-pyran-4-yl)-3-methoxybut-2-enoate S35
7-Hydroxy-9-methoxy-6*H*-benzo[*c*]chromen-6-one (21) S36
Methyl (*E*)-3-methoxy-4-(2-oxochroman-4-yl)but-2-enoate S37
7-Hydroxy-3,9-dimethoxy-6H-benzo[c]chromen-6-one (23) S38
Methyl (*E*)-3-methoxy-4-(7-methoxy-2-oxochroman-4-yl)but-2-enoate S39
7-Hydroxy-3,9-dimethoxy-1-methyl-6*H*-benzo[*c*]chromen-6-one (24) S40
Methyl (*E*)-3-methoxy-4-(7-methoxy-5-methyl-2-oxochroman-4-yl)but-2-enoate S41
7-Hydroxy-1,9-dimethoxy-3-methyl-6*H*-benzo[*c*]chromen-6-one (27) S42
Methyl (*E*)-3-methoxy-4-(5-methoxy-7-methyl-2-oxochroman-4-yl)but-2-enoate S43

6. Computational Details S44

7. Functional Test for the $AlMe_3$-Catalysed Reaction S44

8. Interaction of $AlMe_3$ with the Reactants S44

9. Calculated Energies and Geometries for the Uncatalysed Reaction S45

9.1 Lactone 7 ..... S45
9.2 Brassard's diene (8) S45
9.3 Transition State $TS1_{endo}$ S46
9.4 Transition State $TS1_{exo}$ S47
9.5 Cycloadduct *endo*-I1 ..... S48
9.6 Cycloadduct *exo*-I1 ..... S49

10. Calculated Energies and Geometries for the Brønsted-Acid Catalysed Reaction S51

10.1 Bis(trifluoromethanesulfonyl)methane Tf_2CH_2 ..... S51
10.2 Bis(trifluoromethanesulfonyl)methane Anion S51
10.3 Triflic Acid S52
10.4 Triflate Anion S52
10.5 HCl S52
10.6 Chloride Anion S53
10.7 Oxonium Ion S53
10.8 Water S53
10.9 Protonated Lactone 7-H^+ S54
10.10 Transition State TS2a leading to *E*-Intermediate S54
10.11 Transition State leading to *Z*-Intermediate S55
10.12 Intermediate (*E*)-13-TMS^+ S56
10.13 Intermediate (*Z*)-13-TMS^+ S57
10.14 Transition State TS2b S58
10.15 Protonated Cycloadduct *exo*-I1-H^+ S59
10.16 Protonated Cycloadduct *endo*-I1-H^+ S60

11. Calculated Energies and Geometries for the $AlMe_3$-Catalysed Reaction S62

11.1 Trimethylaluminium $AlMe_3$ S62
11.2 Lactone-$AlMe_3$ Complex 7-$AlMe_3$ S62
11.3 Brassard-$AlMe_3$ Complex via OTMS-Group S63
11.4 Brassard-$AlMe_3$ Complex via OMe-Group (Enol Ether) S64
11.5 Brassard-$AlMe_3$ Complex via OMe-Group (Silyl Ketene Acetal) S65
11.6 Transition State TS3a leading to *E*-Intermediate S66
11.7 Transition State leading to *Z*-Intermediate S67
11.8 Intermediate (*E*)-13-$AlMe_3$ S68
11.9 Intermediate (*Z*)-13-$AlMe_3$ S70
11.10 Transition State TS3b S71
11.11 Cycloadduct *exo*-I1-$AlMe_3$ S72
11.12 Cycloadduct *endo*-I1-$AlMe_3$ S73

12. Calculated Energies and Geometries for the Formation of the Active Catalyst and its Brønsted Acidity S75
12.1 Tf_2CH_2-$AlMe_3$ Complex S75
12.2 Aluminum Methide I2 .... S75
12.3 Methane S76
12.4 Transition State TS4 .... S76
12.5 Isomeric Catalyst Species I3 .... S77
12.6 Deprotonated I2 .... S78
12.7 Deprotonated I3 .... S78

13. Calculated Energies and Geometries for the I2-Catalysed Reaction S80
13.1 Lactone 7-AlC S80
13.2 Transition State TS4a S80
13.3 Intermediate (*E*)-13-AlC S82
13.4 Transition State TS4b S83
13.5 Cycloadduct *endo*-I2-AlC S85

14. Calculated Energies and Geometries for the I3-Catalysed Reaction S87
14.1 Lactone 7-AlO S87
14.2 Transition State 5a S87
14.3 Intermediate (*E*)-13-AlO S89
14.4 Transition State TS5b S90
14.5 Cycloadduct *endo*-I1-AlO S92

15. References S94

1. General Information

Unless otherwise specified the reactions were carried out using standard Schlenk techniques under dry N_2 with magnetic stirring. Glassware was oven dried at 120 °C overnight. Solvents were dried and purified by conventional methods prior to use. All reagents were used as purchased from commercial suppliers without further purification. Common solvents for chromatography [*n*-pentane, EtOAc] were distilled prior to use. Flash column chromatography was performed on silica gel 60, 0.040–0.063 mm (230–400 mesh). TLC (monitoring the course of the reaction) was performed on precoated plastic sheets with detection by UV (254 nm) and/or by coloration with cerium molybdenum soln [phosphomolybdic acid (25 g), $Ce(SO_4)_2$ H_2O (10 g), conc. H_2SO_4 (60 mL), H_2O (940 mL)]. ^1H and ^{13}C NMR spectra were recorded at r.t. in $CDCl_3$ on a spectrometer at 600 and 151 MHz respectively, relative to internal standard TMS (^1H: δ(TMS) = 0.00) or relative to the resonance of the solvent [^{13}C: δ($CDCl_3$) = 77.0]. Higher order δ and *J* values are not corrected. ^{13}C signals were assigned by means of C, H, COSY and HSQC, or HMBC or NOESY spectroscopy. Enantiomeric excesses were determined by HPLC analysis using Dionex (UltiMate® 3000) equipped with a Chiralpak IC, 250 x 4.6 mm, Daicel column. Optical rotations were measured using a quartz cell with 0.5 mL capacity and a 1 cm path length. Melting points are uncorrected. Synthesis of lactones was accomplished according to literature procedures or are commercially available.[1-3]

1.1 General Procedure for the catalyst preparation

In a dried and nitrogen flushed *Schlenk* flask 10 mol% Tf_2CH_2 (14 mg, 0.05 mmol) were dissolved in 1 mL dry toluene. 20 mol% $AlMe_3$ (2 M solution in hexane, 50 µL, 0.1 mmol) were added by Hamilton syringe. The mixture was stirred for 30 min at room temperature. Additionally, 0.5 mmol (1.0 equiv.) lactone and 250 mg (2.5 equiv., 1.25 mmol) Brassard's diene were added to the catalyst solution. After full conversion of the starting material (monitored by proton NMR), the reaction mixture was quenched by addition of 750 µL TBAF-solution (1 M in THF, 1.5 equiv., 0.75 mmol). The mixture was stirred for 15 min at room temperature and 1.5 equiv. of water were added. After extraction with ethyl acetate (3 x 5 mL) and washing with brine (1 x 5 mL) the combined organic layers were dried by $MgSO_4$ and filtered. The solvent was evaporated under vacuum to give the crude product. After column chromatography (*n*-pentane: EtOAc 90:10) the pure (*E*)-*Michael*-product could be isolated together with the crude *Diels-Alder* product as the minor product.

1.2 General procedure for the deprotonation-cyclisation of the (*E*)-*Michael*-product

In a dried, nitrogen flushed *Schlenk*-flask containing dry THF (20 µL/µL *n*-BuLi) 1.6 equivalents HMDS were diluted and cooled to -78 °C. At -78 °C 1.5 equivalents of *n*-BuLi were added. The LHMDS was stirred for 15 minutes at -78 °C. Afterwards 1.0 equivalent of (*E*)-*Michael*-product in dry THF (100 µL/2 mg (*E*)-*Michael*-product) have been added at -78 °C. The solution turned red-orange and was stirred for 2 h at -78 °C. Afterwards the acetone-dry-ice-bath was removed and reaction mixture was allowed to come to room temperature overnight. After full conversion of the starting material (TLC), the mixture was quenched with ammonium chloride solution and extracted with ethyl acetate (3 x 1 mL/mg starting material EtOAc). The combined organic layers were dried with magnesium sulphate, filtered and concentrated *in vacuo*. The crude product was purified by flash column chromatography (*n*-pentane:EtOAc 90:10).

1.3 General procedure for the oxidation with DDQ

In a round bottom flask 1.0 equivalent of *Diels-Alder* product was dissolved in toluene (1 mL/0.1 mmol DDQ). Additionally, 1.3 equivalents of DDQ were added and the mixture was stirred at room temperature for 4 h. After full conversion of the starting material (TLC), the reaction was quenched by adding sodium hydrogen carbonate solution and stirred for 1 h at room temperature. The mixture was extracted with Et_2O (3 x 5 mL) and washed with brine (1 x 5 mL). The combined organic layers were dried with $MgSO_4$, filtered and the solvent was removed under vacuum. The crude product was purified by flash column chromatography (*n*-pentane:EtOAc 90:10) to give pure isocoumarines.

2. Optimisation Studies

2.1 Workup and Oxidation Condition Screening

It was observed that the workup conditions were strongly influencing the yield. Two main points were considered, a) hydrolysis of the silyl ketene acetal (Table S2) and b) oxidation towards the aromatic product (Table S1). For oxidation we tested ceric ammonium nitrate (1.3 equiv., 16 h, 19%), oxygen (direct gassing into reaction vessel, 16 h, 17%) and 2,3-dichloro-5,6-dicyano-1,4-benzoquinone (DDQ) (1.3 equiv., 4 h, quant.). Due to the fact, that DDQ provided the desired isocoumarin **14** in quantitative yield from 8-hydroxy-6-methoxy-3,4,4a,5-tetrahydro-1*H*-isochromen-1-one (**12**), we exclusively used DDQ for further oxidations.

Table S1. Conditions for Oxidation towards isocoumarines
(CAN: ceric ammonium nitrate; * direct gassing into the vessel)

entry	oxidant	equivalents	time	conversion	yield
1	DDQ	1.3	4 h	full	quant.
2	O_2	-*	16 h	19%	19%
3	CAN	1.3	16 h	17%	17%

Table S2 summarizes the conditions tested for the hydrolysis of the silyl derivatives leading first to 6-hydroxy-8-methoxy-3,4,4a,5-tetrahydro-1*H*-isochromen-1-one (**12**) and after oxidation to 8-hydroxy-6-methoxy-3,4,4a,5-tetrahydro-1*H*-isochromen-1-one (**14**). Starting from the initial result using tetra-*n*-butylammonium fluoride (TBAF) in THF and a yield of 20% over two steps (entry 1), we tested alternative reagents. Obviously, acidic conditions or hydrogen fluoride pyridine (entries 2-4) gave only unsatisfactory results. Changing to tetra-*n*-butylammonium fluoride trihydrate did not improve the yield compared to the initial results (entry 5, 17%), but buffering the basic fluoride solution with trifluoroacetic acid (TFA) did (entry 6, 25%). However, when directly oxidizing the crude mixture with DDQ without isolation intermediate **12** (entries 7+8), the original setup proved superior and isochromen-1-one **14** was isolated in moderate 30% yield (entry 7).

Table S2. Diels-Alder reaction of Brassard’s diene (**8**) and 5,6-dihydro-2*H*-pyran-2-one (**7**).

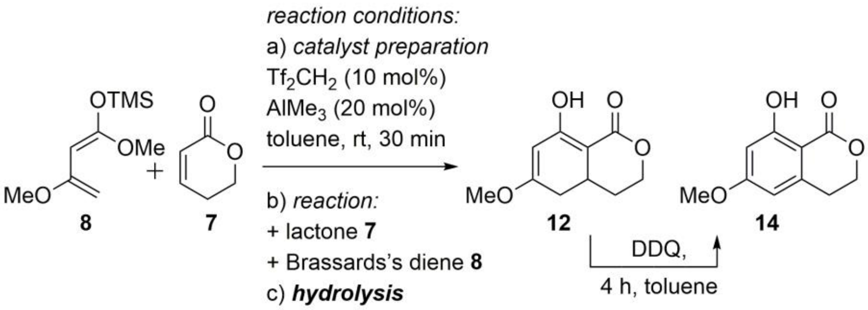

entry	hydrolysis condition (0.5 h, 1.5 equiv)	yield
1	TBAF in THF (1.0 M)	20%[a]
2	aq. HCl (1.0 M)	5%[a]
3	SiO_2	4%[a]
4	HF-pyridine	2%[a]
5	TBAF x 3 H_2O in toluene (1.0 M)	17%[a]
6	TBAF x 3 H_2O in toluene (1.0 M) + TFA (1:1)	25%[a]
7	TBAF x 3 H_2O in toluene (1.0 M) + TFA (1:1)	16%[b]
8	TBAF in THF	30%[b]

[a] yield after 2 isolated steps; [b] yields after 2 steps without isolation of **12**

2.2 Cyclisation of the Vinylogous Michael-Product

Proving the configuration of the products and ultimately obtaining increased yields of the isocoumarin **14**, it was hypothesised that the major isomer methyl (*E*)-3-methoxy-4-(2-oxotetrahydro-2*H*-pyran-4-yl)but-2-enoate [(*E*)-**13**] could be deprotonated at the α-carbon of the lactone, subsequently leading to the desired 6-hydroxy-8-methoxy-3,4,4a,5-tetrahydro-1*H*-isochromen-1-one (**12**) via a Dieckmann condensation, which should be readily oxidised via DDQ to the corresponding isocoumarin **14** in a consecutive step. The corresponding (*Z*)-isomer (*Z*)-**13** should not be amenable to this transformation, which could be proven experimentally. For the deprotonation-cyclization approach different bases were screened (Table S2). In a first assay, five different bases were screened (entries 1-5): While lithium diisopropylamide (LDA)[35] (entry 1, 20%), sodium ethoxide (entry 2, no product formation), potassium bis(trimethylsilyl)amide (KHMDS) (entry 3, no product formation), and potassium *tert*-butoxide (entry 4, 59%, impure product) did either not form 6-hydroxy-8-methoxy-3,4,4a,5-tetrahydro-1*H*-isochromen-1-one (**12**) or in low purity or in low yield, respectively, lithium bis(trimethylsilyl)amide (LHMDS)[36, 37] (entry 5, 58%) looked more promising. Increasing the number of equivalents (to 1.5 equiv) and the reaction time to 16 h (entries 6-8) finally provided product **12** in 92% yield.

Table S3. Dieckmann reaction vinylogous (*E*)-Michael-product (***E***)-**13**.

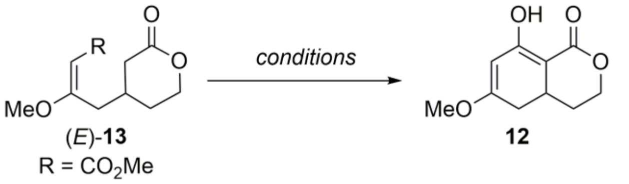

entry	conditions (in THF)	yield
1	LDA (1.1 equiv), -78 °C to rt, 4.5 h	20%
2	NaOEt (1.1 equiv), -78 °C to rt, 16 h	-
3	KHMDS (1.1 equiv), -78 °C to 10 °C, 19 h	-
4	KO^{t}Bu (1.1 equiv), -40 °C to rt, 2 h	59%[a]
5	LHMDS (1.1 equiv), -78 °C to rt, 3.5 h	58%
6	LHMDS (1.25 equiv), -78 °C to rt, 3.5 h	70%
7	LHMDS (1.5 equiv), -78 °C to -25 °C, 2 h	85%
8	LHMDS (1.5 equiv), -78 °C to rt), 16 h	92%

[a] yield of crude product

3. Products

8-Hydroxy-6-methoxy-3,4,4a,5-tetrahydro-1*H*-isochromen-1-one (12)

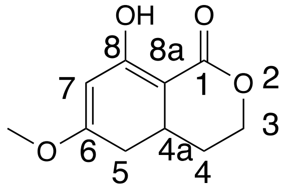

^{1}H-NMR (CDCl$_3$, 600 MHz): δ [ppm] = 1.80 (ddt, $^2J_{4a,4b}$= 13.3 Hz, $^3J_{4a,3}$= 4.0 Hz, $^3J_{4a,5}$= 1.1 Hz, 1 H, 4-H$_a$), 2.00 (ddt, $^2J_{4b,4a}$= 13.3 Hz, $^3J_{4b,5}$= 4.1 Hz, $^3J_{4b,3}$= 2.1 Hz, 1 H, 4-H$_b$), 2.28 (m, 1 H, 6-Ha), 2.30 (m, 1 H, 6-H$_b$), 2.95 (m, 1 H, 5-H), 3.72 (s, 3 H, O*CH$_3$*), 4.19 (ddd, $^2J_{3a,3b}$= 11.3 Hz, $^3J_{3a,4a}$= 4.0 Hz, $^3J_{3a,4b}$= 2.1 Hz, 1 H, 3-H$_a$), 4.44 (ddd, $^2J_{3b,3a}$= 11.3 Hz, $^3J_{3b,4a}$= 4.0 Hz, $^3J_{3b,4b}$= 2.1 Hz, 1 H, 3-H$_b$), 5.18 (s, 1 H, 8-H), 13.23 (s, 1 H, OH).
^{13}C-NMR (CDCl$_3$, 151 MHz): δ [ppm] = 29.7 (C-4), 30.9 (C-5), 35.0 (C-6), 55.8 (O*C*H$_3$), 67.7 (C-3), 87.2 (C-8), 93.7 (C-10), 170.3 (C-7), 171.4 (C-1), 173.2 (C-9).
IR (ATR Film): $\tilde{\nu}$ [cm^{-1}] = 2955, 1715 (C=O), 1622, 1438, 1378, 1343, 1259, 1140 (C-O), 1101, 1074, 1053, 969, 931, 842, 743, 707.
GC-MS (EI, positive ion, 70 eV): *m/z* (%) = 196 (46) [M$^+$].
HRMS (ESI, positive on): found: 197.0811 [(M+H)$^+$], calc: 197.0808 [(M+H)$^+$].
R$_f$= 0.08 (PE:EtOAc 80:20)
yellow oil

8-Hydroxy-6-methoxyisochroman-1-one (14)

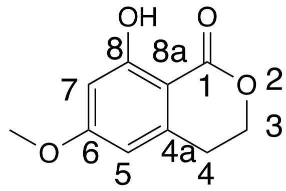

^{1}H-NMR (CDCl$_3$, 600 MHz): δ [ppm] = 2.99 (t, $^3J_{4,3}$= 6.1 Hz, 2 H, 4-H), 3.83 (s, 3 H, 11-H), 4.52 (t, $^3J_{3,4}$= 6.1 Hz, 2 H, 3-H), 6.28 (d, $^3J_{5,7}$= 2.4 Hz, 1 H, 5-H), 6.38 (d, $^3J_{7,5}$= 2.4 Hz, 1 H, 7-H), 11.19 (s, 1 H, OH).
^{13}C-NMR (CDCl$_3$, 151 MHz): δ [ppm] = 27.9 (C-4), 55.6 (O*C*H$_3$-6), 67.6 (C-3), 99.5 (C-7), 101.9 (C-8a), 106.1 (C-5), 141.2 (C-4a), 164.7 (C-8), 165.8 (C-6), 169.5 (C-1).
IR (ATR Film): $\tilde{\nu}$ [cm^{-1}] = 3088, 2970, 2919, 2851, 2236, 1727, 1663, 1628 (C=O), 1583, 1510, 1474, 1440, 1429, 1415, 1382, 1340, 1306, 1249, 1201, 1158, 1111, 1072, 1037, 1026, 960, 918, 892, 850, 799, 745, 710, 699, 667.
GC-MS (EI, positive ion, 70 eV): *m/z* (%) = 194 (65) [M$^+$], 164 (100) [($C_9H_8O_3^+$].
HRMS (ESI, positive ion): Found: 195.06519 ($C_{10}H_{11}O_4$) [(M+H)$^+$], Calc.: 195.06519 ($C_{10}H_{11}O_4$) [(M+H)$^+$].
Smp.: 106.2 °C (pink solid)
R$_f$= 0.55 (PE:EtOAc 50:50)

Methyl (*E*)-3-methoxy-4-(2-oxotetrahydro-2*H*-pyran-4-yl)but-2-enoate [(*E*)-13]

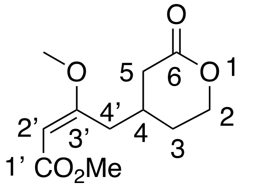

^{1}H-NMR (CDCl$_3$, 600 MHz): δ [ppm] = 1.65 (m, 1 H, 3-H$_a$), 1.93 (ddt, $^2J_{3b,3a}$= 14.2 Hz, $^3J_{3b,4}$= 4.1 Hz, $^3J_{3b,2}$= 1.5 Hz, 1 H, 3-H$_b$), 2.28 (ddd, $^2J_{5a,5b}$= 17.1 Hz, $^3J_{5a,4}$= 10.9 Hz, $^3J_{5a,4'a}$= 1.1 Hz, 1 H, 5-H$_a$), 2.35 (m, 1 H, 4-H), 2.66 (ddd, $^2J_{5b,5a}$= 17.1 Hz, $^3J_{5b,4}$= 5.4 Hz, $^4J_{5b,4'b}$= 1.7 Hz, 1 H, 5-H$_b$), 2.81 (ddd, $^2J_{4'a,4'b}$= 13.1 Hz, $^3J_{4'a,4}$= 7.5 Hz, $^2J_{4'a,5a}$= 1.1 Hz, 1 H, 4'-H$_a$), 2.87 (ddd, $^2J_{4'b,4'a}$= 13.1 Hz, $^3J_{4'b,4}$= 6.9 Hz, $^3J_{4'b,5b}$= 1.7 Hz, 1 H, 4'-H$_b$), 3.64 (s, 3 H, 1'-OCH$_3$), 3.68 (s, 3 H, 3'-OCH$_3$), 4.25 (tdd, $^2J_{2a,2b}$= 11.7 Hz, $^3J_{2a,3}$= 3.8 Hz, $^3J_{2a,4}$= 1.1 Hz, 1 H, 2-H$_a$), 4.43 (ddd, $^2J_{2b,2a}$= 11.7 Hz, $^3J_{2b,3}$= 4.1 Hz, $^3J_{2b,4}$= 1.0 Hz, 1 H, 2-H$_b$), 5.10 (s, 1 H, 2'-H).
^{13}C-NMR (CDCl$_3$, 151 MHz): δ [ppm] = 28.7 (C-3), 30.4 (C-4), 36.0 (C-5), 37.3 (C-4'), 51.0 (O*C*H$_3$-3'), 55.6 (O*C*H$_3$-1'), 68.5 (C-2), 92.0 (C-2'), 167.9 (C-3'), 171.0 (C-6), 173.1 (C-1').
IR (ATR Film): $\tilde{\nu}$ [cm^{-1}] = 2950 (C=O), 1734, 1707 (C=O), 1619, 1436, 1402, 1378, 1287, 1251, 1220, 1191, 1135, 1081, 1049, 1011, 974, 929, 823, 782, 746, 680.
HRMS (ESI, positive ion): Found: 251.08895 ($C_{11}H_{16}O_5Na$) [(M+Na)$^+$], Calc.: 251.08899 ($C_{11}H_{16}O_5Na$) [(M+Na)$^+$].
R$_f$= 0.37 (PE:EtOAc 80:20)
yellow oil

Methyl (*Z*)-3-methoxy-4-(2-oxotetrahydro-2*H*-pyran-4-yl)but-2-enoate [(*Z*)-13]

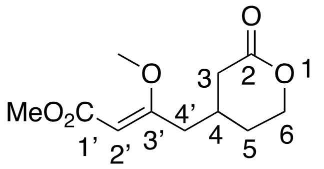

^{1}H-NMR (CDCl$_3$, 600 MHz): δ [ppm] = 1.55 (m, 1 H, 5-H$_a$), 2.01 (ddt, $^2J_{5b,5a}$= 12.3 Hz , $^3J_{5b,6b}$= 3.7 Hz, $^3J_{5b,6b}$= 1.7 Hz, 1 H, 5-H$_b$), 2.21 (m, 3 H, 4'-H, 3-H$_a$), 2.29 (m, 1 H, 4-H), 2.36 (m, 1 H, 4-H), 2.74 (ddd, $^2J_{3b,3a}$= 17.3 Hz, $^3J_{3b,4}$= 5.6 Hz, $^4J_{3b,4'b}$= 1.6 Hz, 1 H, 3-H$_b$), 3.67 (s, 3 H, 3'-OCH$_3$), 3.91 (s, 3 H, 1'-OCH$_3$), 4.26 (td, $^2J_{6a,6b}$= 11.7 Hz, $^3J_{6a,5}$= 3.8 Hz, 1 H, 6-H$_a$), 4.43 (ddd, $^2J_{6b,6a}$= 11.5 Hz, $^3J_{6b,5a}$= 3.7 Hz, $^3J_{6b,4}$= 1.1 Hz, 1 H, 6-H$_b$), 5.00 (s, 1 H, 2'-H).
^{13}C-NMR (CDCl$_3$, 150 MHz): δ [ppm] = 28.4 (C-5), 29.7 (C-4), 36.1 (C-3), 41.3 (C-4'), 51.1 (O*C*H$_3$-3'), 59.4 (O*C*H$_3$-1'), 68.3 (C-6), 97.6 (C-2'), 165.2 (C-3'), 167.7 (C-1'), 170.2 (C-2).
IR (ATR Film): $\tilde{\nu}$ [cm^{-1}] = 2971, 2950 (C=O), 1734 (lactone), 1623 (C=O), 1436, 1402, 1366, 1217 (C-O), 1165, 1142, 1068 (C-O), 974, 952, 918, 827 (RRC=CRH), 521.
HRMS (ESI, positive ion): found: ($C_{11}H_{16}O_5Na$) [(M+Na)$^+$], calc.: ($C_{11}H_{16}O_5Na$) [(M+Na)$^+$].
R$_f$= 0.04 (PE:EE 80:20)
yellow oil

(*R*)-8-Hydroxy-6-methoxy-3-methylisochroman-1-one [(*R*)-3]

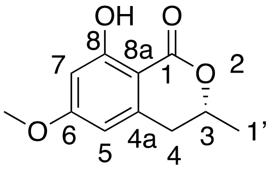

^{1}H-NMR (CDCl$_3$, 600 MHz): δ [ppm] = 1.51 (d, $^{3}J_{1',3}$= 6.3 Hz, 3 H, 1'-H), 2.87 (m, 2 H, 4-H), 3.83 (s, 2 H, OCH$_3$), 4.67 (qt, ($^{3}J_{3,4}$= 8.6 Hz, $^{3}J_{3,1'}$= 6.3 Hz, 1 H, 3-H), 6.25 (m, 1 H, 5-H), 6.37 (d, $^{4}J_{7,5}$= 2.3 Hz 1 H, 7-H), 11.25 (s, 1 H, 8-H$_{OH}$).
^{13}C-NMR (CDCl$_3$, 151 MHz): δ [ppm] = 20.73 (C-1'), 34.9 (C-4), 55.6 (C-O*C*H$_3$), 75.5 (C-3), 99.4 (C-7), 101.6 (C-8a), 106.2 (C-5), 140.9 (C-4a), 164.6 (C-8), 165.8 (C-6), 169.9 (C-1).
IR (ATR Film): $\tilde{\nu}$ [cm^{-1}] = 2978 (OH, Chelat), 2920, 2851, 1644 (C=O), 1627, 1582, 1532, 1494, 1470, 1437, 1422, 1393, 1370 (OH), 1338, 1313, 1248 (C-O), 1202, 1189, 1154, 1117, 1065, 1027, 961, 934, 909, 898, 833, 802, 774, 709, 700, 670.
HRMS (ESI, positive ion): Found: 209.0806 ($C_{11}H_{13}O_4$) [(M+H)$^+$], Calc.: 209.0808 ($C_{11}H_{13}O_4$) [(M+H)$^+$].
$[\alpha]_D^{20}$= -52.0 (c= 0.27, CHCl$_3$)
HPLC (91%*ee;* Chiralpak IC, heptane/*i*PrOH 95:05, Flowrate = 0.5 mL/min, λ = 217 nm): tR [(*S*)] = 82.8 min, tR [(*R*)] = 87.7 min)
R$_f$= 0.47 (PE:EtOAc 80:20)
clear oil

Methyl (*E*)-3-methoxy-4-((2*R*)-2-methyl-6-oxotetrahydro-2*H*-pyran-4-yl)but-2-enoate

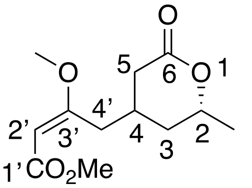

^{1}H-NMR (CDCl$_3$, 600 MHz): δ [ppm] = 1.36 (d, $^{3}J_{Me,2}$= 6.2 Hz, 3 H, 2-CH$_3$), 1.72 (m, 2 H, 3-H), 2.30 (dd, $^{2}J_{5a,4'b}$= 16.3 Hz, $^{3}J_{5a,4}$= 9.4 Hz, 1 H, 5-H$_a$), 2.45 (m, 1 H, 4-H), 2.54 (dd, $^{2}J_{5b,5a}$= 16.3 Hz, $^{3}J_{5b,4}$= 6.0 Hz, 1 H, 5-H$_b$), 2.86 (m, 2 H, 4'-H), 3.64 (s, 3 H, 3'-OCH$_3$), 3.67 (s, 3 H, 1'-CO$_2$CH$_3$), 4.59 (dt, $^{3}J_{2,3}$= 6.8 Hz, $^{3}J_{2,Me}$= 6.2 Hz, 1 H, 2-H), 5.10 (s, 1 H, 2'-H).
^{13}C-NMR (CDCl$_3$, 151 MHz): δ [ppm] = 21.2 (C-2-*C*H$_3$), 27.4 (C-4), 34.6 (C-3), 34.8 (C-5), 37.1 (C-4'), 51.0 (CO$_2$*C*H$_3$-1'), 56.0 (O*C*H$_3$-3'), 73.6 (C-2), 92.1 (C-2'), 167.9 (C-1'), 172.4 (C-6), 173.2 (C-3').
IR (ATR Film): $\tilde{\nu}$ [cm^{-1}] = 2946 (OH, Chelat), 1737 (lactone), 1708 (C=O), 1619, 1436, 1378, 1287, 1249, 1218, 1293, 1135 (C-O-C), 1099, 1079, 1050, 983, 930, 823 (RRC=CRH), 749, 704.
HRMS (ESI, positive ion): Found: 243.1232 ($C_{12}H_{19}O_5$) [(M+H)$^+$], Calc.: 243.1227 ($C_{12}H_{19}O_5$) [(M+H)$^+$].
$[\alpha]_D^{20}$= -35.0 (c= 0.8, CHCl$_3$)
R$_f$= 0.10 (PE:EtOAc 80:20)
clear oil

(*S*)-8-Hydroxy-6-methoxy-3-methylisochroman-1-one [(*S*)-4]

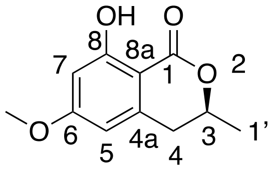

^{1}H-NMR (CDCl$_3$, 600 MHz): δ [ppm] = 1.51 (d, $^3J_{1',3}$= 6.3 Hz, 3 H, 1'-H), 2.87 (m, 2 H, 4-H), 3.83 (s, 2 H, OCH$_3$), 4.67 (qt, $^3J_{3,4}$= 8.6 Hz, $^3J_{3,1'}$= 6.3 Hz, 1 H, 3-H), 6.25 (m, 1 H, 5-H), 6.37 (d, $^4J_{7,5}$= 2.3 Hz 1 H, 7-H), 11.25 (s, 1 H, 8-H$_{OH}$).
^{13}C-NMR (CDCl$_3$, 151 MHz): δ [ppm] = 20.73 (C-1'), 34.9 (C-4), 55.6 (C-O*C*H$_3$), 75.5 (C-3), 99.4 (C-7), 101.6 (C-8a), 106.2 (C-5), 140.9 (C-4a), 164.6 (C-8), 165.8 (C-6), 169.9 (C-1).
IR (ATR Film): $\tilde{\nu}$ [cm^{-1}] = 2979 (OH, chelate), 2919, 2851, 1738, 1659 (C=O), 1628, 1582, 1509, 1455, 1439, 1370 (OH), 1295, 1241 (C-O), 1241, 1195, 1156, 1113, 1095, 1069, 1028, 964, 939, 915, 899, 848, 817, 799, 759, 700, 669.
HRMS (ESI, positive ion): Found: 209.0810 ($C_{11}H_{13}O_4$) [(M+H)$^+$], Calc.: 209.0808 ($C_{11}H_{13}O_4$) [(M+H)$^+$].
$[\alpha]_D^{20}$= +14.5 (c= 0.45, CHCl$_3$)
HPLC (96 %*ee;* Chiralpak IC, heptane/*i*PrOH 95:05, Flowrate = 0.5 mL/min, λ = 217 nm): tR [(*S*)] = 82.8 min, tR [(*R*)] = 87.7 min)
R_f= 0.47 (PE:EtOAc 80:20)
clear oil

Methyl (*E*)-3-methoxy-4-((2*S*)-2-methyl-6-oxotetrahydro-2*H*-pyran-4-yl)but-2-enoate

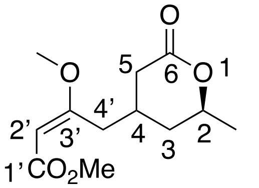

^{1}H-NMR (CDCl$_3$, 600 MHz): δ [ppm] = 1.36 (d, $^3J_{Me,2}$= 6.2 Hz, 3 H, 2-CH$_3$), 1.72 (m, 2 H, 3-H), 2.30 (dd, $^2J_{5a,4'b}$= 16.3 Hz, $^3J_{5a,4}$= 9.4 Hz, 1 H, 5-H$_a$), 2.45 (m, 1 H, 4-H), 2.54 (dd, $^2J_{5b,5a}$= 16.3 Hz, $^3J_{5b,4}$= 6.0 Hz, 1 H, 5-H$_b$), 2.86 (m, 2 H, 4'-H), 3.64 (s, 3 H, 3'-OCH$_3$), 3.67 (s, 3 H, 1'-CO$_2$CH$_3$), 4.59 (dt, $^3J_{2,3}$= 6.8 Hz, $^3J_{2,Me}$= 6.2 Hz, 1 H, 2-H), 5.10 (s, 1 H, 2'-H).
^{13}C-NMR (CDCl$_3$, 151 MHz): δ [ppm] = 21.2 (C-2-*C*H$_3$), 27.4 (C-4), 34.6 (C-3), 34.8 (C-5), 37.1 (C-4'), 51.0 (CO$_2$*C*H$_3$-1'), 56.0 (O*C*H$_3$-3'), 73.6 (C-2), 92.1 (C-2'), 167.9 (C-1'), 172.4 (C-6), 173.2 (C-3').
IR (ATR Film): $\tilde{\nu}$ [cm^{-1}] = 2946 (OH, Chelat), 1737 (lactone), 1708 (C=O), 1619, 1436, 1378, 1287, 1249, 1218, 1293, 1137 (C-O-C), 1099, 1079, 1050, 983, 930, 824 (RRC=CRH), 749, 704.
HRMS (ESI, positive ion): Found: 243.1228 ($C_{12}H_{19}O_5$) [(M+H)$^+$], Calc.: 243.1227 ($C_{12}H_{19}O_5$) [(M+H)$^+$].
$[\alpha]_D^{20}$= +30.5 (c= 1.0, CHCl$_3$)
R_f= 0.10 (PE:EtOAc 80:20)
clear oil

8-Hydroxy-6-methoxy-3-pentylisochroman-1-one (16)

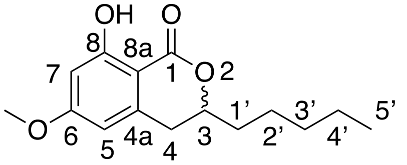

^{1}H-NMR (CDCl$_3$, 600 MHz): δ [ppm] = 0.90 (t, $^{3}J_{5',4'}$= 6.8 Hz, 3 H, 5'-H), 1.33 (m, 4 H, 4'-H, 3'-H), 1.46 (dddd, $^{4}J_{2'a,2'b}$= 13.6 Hz, $^{3}J_{2'a,1'a}$= 10.7 Hz, $^{3}J_{2'a,1'b}$= 8.4 Hz, $^{3}J_{2'a,3'}$= 5.7 Hz, 1 H, 2'-H$_a$), 1.52 (m, 1 H, 2'-H$_b$), 1.70 (dddd, $^{2}J_{1'a,1'b}$= 13.9 Hz, $^{3}J_{1'a,2'a}$= 10.7 Hz, $^{3}J_{1'a,3}$= 5.5 Hz, $^{3}J_{1'a,2'b}$= 2.2 Hz, 1 H, 1'-H$_a$), 1.86 (m, 1 H, 1'-H$_b$), 2.86 (m, 2 H, 4-H), 3.82 (s, 3 H, 6-OCH$_3$), 4.51 (m, 1 H, 3-H), 6.25 (d, $^{3}J_{5,7}$= 2.2 Hz, 1 H, 5-H), 6.37 (d, $^{3}J_{7,5}$= 2.2 Hz, 1 H, 7-H), 11.25 (s, 1 H, 8-OH).
^{13}C-NMR (CDCl$_3$, 151 MHz): δ [ppm] = 14.0 (C-5'), 22.5 (C-4'), 24.5 (C-2'), 31.5 (C-3'), 33.2 (C-4), 34.7 (C-1'), 55.5 (O*C*H$_3$-6), 79.2 (C-3), 99.4 (C-7), 101.9 (C-8a), 106.2 (C-5), 141.1 (C-4a), 164.5 (C-8), 165.7 (C-6), 170.0 (C-1).
IR (ATR Film): ṽ [cm^{-1}] = 3458 (OH), 3017, 2971, 2951 (OH, Chelat), 2869, 1738 (lactone), 1648 (C=O), 1625, 1583, 1518, 1456, 1436, 1371 (OH), 1316, 1255, 1217 (C-O), 1204, 1155, 1129, 055, 1035, 1013, 985, 952, 904, 852, 827, 801, 759, 726, 701, 676.
HRMS (ESI, positive ion): Found: 265.1437 ($C_{15}H_{21}O_4$) [(M+H$^+$)], Calc.: 265.1434 ($C_{15}H_{21}O_4$) [(M+H$^+$)].
clear oil
R_f= 0.54 (PE:EtOAc 80:20)

Methyl (*E*)-3-methoxy-4-(2-oxo-6-pentyltetrahydro-2*H*-pyran-4-yl)but-2-enoate

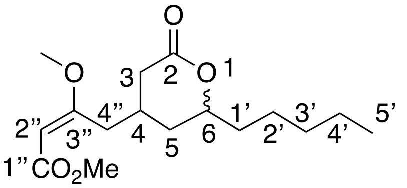

^{1}H-NMR (CDCl$_3$, 600 MHz): δ [ppm] = 0.89 (t, $^{3}J_{5',4'}$= 6.8 Hz, 3 H, 5'-H), 1.31 (m, 5 H, 2'-Hb, 3'-H, 4'-H), 1.51 (m, 2 H, 1'-H$_a$, 2'-H$_b$), 1.72 (m, 3 H, 5-H, 1'-H$_b$), 2.32 (dd, $^{2}J_{3a,3b}$= 16.2 Hz, $^{3}J_{3a,4}$= 9.5 Hz, 1 H, 3-H$_a$), 2.43 (m, 1 H, 4-H), 2.54 (dd, $^{2}J_{3b,3a}$= 16.2 Hz, $^{3}J_{3b,4}$= 6.0 Hz, 1 H, 3-H$_b$), 2.88 (d, $^{3}J_{4'',4}$= 7.5 Hz, 2 H, 4''-H), 3.64 (s, 3 H, 1''-OCH$_3$), 3.68 (s, 3 H, OCH$_3$), 4.40 (tt, $^{3}J_{6,1'}$= 8.9 Hz, $^{3}J_{6,5}$= 4.5 Hz, 1 H, 6-H), 5.11 (s, 1 H, 2''-H).
^{13}C-NMR (CDCl$_3$, 151 MHz): δ [ppm] = 14.0 (C-5'), 22.5 (C-4'), 24.8 (C-2'), 27.5 (C-4), 31.6 (C-3'), 32.9 (C-5), 35.0 (C-3), 35.4 (C-1'), 37.1 (C-4''), 51.0 (O*C*H$_3$-3''), 55.6 (O*C*H$_3$-1''), 77.3 (C-6), 92.1 (C-2''), 167.9 (C-3''), 172.7 (C-2), 173.3 (C-1'').
IR (ATR Film): ṽ [cm^{-1}] = 3457 (OH), 3017, 2971, 2947 (OH, Chelat), 1738 (lactone), 1621 (C=O), 1435, 1372 (OH), 1287, 1229, 1217 (C-O), 1135, 1051, 930, 822, 750.
HRMS (ESI, positive ion): Found: 299.1851 ($C_{16}H_{27}O_5$) [(M+H$^+$)], Calc.: 299.1853 ($C_{16}H_{27}O_5$) [(M+H$^+$)].
clear oil
R_f= 0.19 (PE:EtOAc 80:20)

(*S*)-3-(2-((*tert*-Butyldimethylsilyl)oxy)ethyl)-8-hydroxy-6-methoxyisochroman-1-one [(*S*)-18]

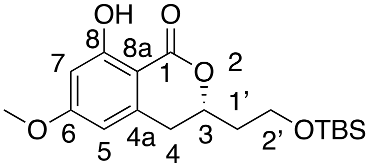

^{1}H-NMR (CDCl$_3$, 600 MHz): δ [ppm] = 0.06 (s, 6 H, Si-(CH$_3$)$_2$-H), 0.88 (s, 9 H, C-(CH$_3$)$_3$-H), 1.90 (ddt, $^2J_{1'a,1'b}$= 13.8 Hz, $^3J_{1'a,2'}$= 8.1 Hz, $^3J_{1'a,3}$= 5.2 Hz, 1 H, 1'-H$_a$), 2.04 (m, 1 H, 1'-Hb), 2.90 (m, 2 H, 4-H), 3.80 (m, 1 H, 2'-H$_a$), 3.82 (s, 3 H, OCH$_3$), 3.87 (ddd, $^2J_{2'a,2'b}$= 10.1 Hz, $^3J_{2'b,1'a}$= 8.1 Hz,, $^3J_{2'a,1'b}$= 4.6 Hz, 1 H, 2'-H$_b$), 4.71 (ddt, $^3J_{3,4}$= 9.6 Hz, $^3J_{3,1'b}$= 7.6 Hz, $^3J_{3,1'a}$= 5.2 Hz, 1 H, 3-H), 6.25 (d, $^4J_{5,7}$= 2.3 Hz, 1 H, 5-H), 6.37 (d, $^4J_{7,5}$= 2.3 Hz 1 H, 7-H), 11.24 (s, 1 H, 8-H$_{OH}$).
^{13}C-NMR (CDCl$_3$, 151 MHz): δ [ppm] = -5.4 (Si-(*C*H$_3$)$_2$), 18.3 (*C*(CH$_3$)$_3$), 25.9 (C(*C*H$_3$)$_3$), 33.4 (C-4), 37.8 (C-1'), 55.6 (O*C*H$_3$), 58.5 (C-2'), 75.3 (C-3), 99.7 (C-7), 101.8 (C-8a), 106.2 (C-5), 141.1 (C-4a), 164.6 (C-8), 165.8 (C-6), 169.8 (C-1).
IR (ATR Film): $\tilde{\nu}$ [cm^{-1}] = 2928 (OH, Chelat), 2856, 1666 (C=O), 1627, 1583, 1509, 1464, 1439, 1373 (OH), 1311, 1247 (C-O), 1204, 1156, 1089, 1095, 1069, 1035, 1006, 941, 908, 833, 799, 775, 701, 662.
HRMS (ESI, positive ion): Found: 353.1786 ($C_{18}H_{29}O_5Si$) [(M+H)$^+$], Calc.: 353.1779 ($C_{18}H_{29}O_5Si$) [(M+H)$^+$].
$[\alpha]_D^{20}$= -43.0 (c= 1.0, CHCl$_3$)
HPLC (94 %*ee;* Chiralpak IC, heptane/*i*PrOH 80:20, Flowrate = 0.5 mL/min, λ = 217 nm, 265 nm): tR [(*S*)] = 14.8 min, tR [(*R*)] = 16.3 min)
clear oil
R$_f$= 0.37 (PE:EtOAc 80:20)

Methyl (*E*)-4-((2*S*)-2-(2-((*tert*-butyldimethylsilyl)oxy)ethyl)-6-oxotetrahydro-2*H*-pyran-4-yl)-3-methoxybut-2-enoate

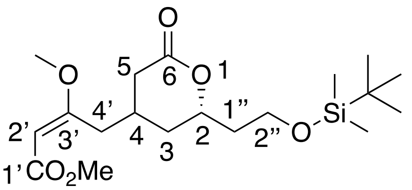

^{1}H-NMR (CDCl$_3$, 600 MHz): δ [ppm] = 0.05 (s, 6 H, Si-(CH$_3$)$_2$), 0.89 (s, 9 H, C-(CH$_3$)$_3$), 1.74 (m, 2 H, 1a''-H, 3-H$_a$), 1.81 (dt, $^4J_{1''b,1''a}$= 15.5 Hz, $^3J_{1''b,2}$= 8.0 Hz, 1 H, 1''-H$_b$), 1.89 (ddt, $^4J_{3b,3a}$= 13.7 Hz, $^3J_{3b,2}$= 9.4 Hz, $^3J_{3b,4}$= 5.2 Hz, 1 H, 3-H$_b$), 2.32 (dd, $^4J_{4'a,4'b}$= 16.2 Hz, $^3J_{4'a,4}$= 9.8 Hz, 1 H, 4'-H$_a$), 2.44 (m, 1 H, 4-H), 2.54 (dd, $^4J_{4'b,4'a}$= 16.2 Hz, $^3J_{4'b,4}$= 5.8 Hz, 1 H, 4'-H$_b$), 2.84 (dd, $^4J_{5a,5b}$= 13.2 Hz, $^3J_{5a,4}$= 6.9 Hz, 1 H, 5-H$_a$), 2.90 (dd, $^4J_{5b,5a}$= 13.2 Hz, $^3J_{5b,4}$= 8.1 Hz, 1 H, 5-H$_b$), 3.63 (s, 3 H, 1'-OCH$_3$), 3.67 (s, 3 H, 3'-OCH$_3$), 3.73 (m, 1 H, 2''-Ha), 3.78 (m, 1 H, 2''-Hb), 4.61 (dddd, $^3J_{2,3b}$= 9.4 Hz, $^3J_{2,1''b}$= 8.6 Hz, $^3J_{2,1''a}$= 8.0 Hz, $^3J_{2,3a}$= 4.3 Hz, 1 H, 2-H), 5.10 (s, 1 H, 2'-H).
^{13}C-NMR (CDCl$_3$, 151 MHz): δ [ppm] = -5.4 (Si-(*C*H$_3$)$_2$), 18.3 (*C*-(CH$_3$)$_3$), 25.9 (C-(*C*H$_3$)$_3$), 27.6 (C-4), 33.2 (C-1''), 35.0 (C-4'), 37.2 (C-5), 38.4 (C-3), 51.0 (O*C*H$_3$-3'), 55.6 (O*C*H$_3$-1'), 58.9 (C-2''), 74.3 (C-2), 92.1 (C-2'), 167.8 (C-3'), 172.4 (C-6), 173.2 (C-1').
IR (ATR Film): $\tilde{\nu}$ [cm^{-1}] = 2952 (OH, Chelat), 2929, 2857, 1738, 1711 (lactone), 1622 (C=O), 1463, 1436, 1378, 1251, 1217, 1192, 1137 (C-O), 1086, 1052, 1007, 931, 833 (RRC=CRH), 775, 663.

HRMS (ESI, positive ion): Found: 387.2201 ($C_{19}H_{35}O_6Si^+$) [(M+H$^+$)], Calc.: 387.2197 ($C_{19}H_{35}O_6Si^+$) [(M+H$^+$)].
clear oil
R_f= 0.19 (PE:EtOAc 80:20)

(*R*)-3-(2-((*tert*-Butyldimethylsilyl)oxy)ethyl)-8-hydroxy-6-methoxyisochroman-1-one [(*R*)-18]

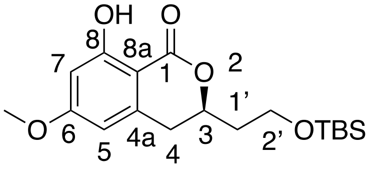

^{1}H-NMR (CDCl$_3$, 600 MHz): δ [ppm] = 0.06 (s, 6 H, Si-(CH_3)$_2$-H), 0.89 (s, 9 H, C-(CH_3)$_2$-H), 1.91 (ddt, $^2J_{1'a,1'b}$= 13.8 Hz, $^3J_{1'a,2'}$= 8.1 Hz, $^3J_{1'a,3}$= 5.2 Hz, 1 H, 1'-H$_a$), 2.05 (m, 1 H, 1'-H$_b$), 2.91 (m, 2 H, 4-H), 3.80 (m, 1 H, 2'-H$_a$), 3.83 (s, 3 H, OCH_3), 3.87 (ddd, $^2J_{2'a,2'b}$= 10.1 Hz, $^3J_{2'b,1'a}$= 8.1 Hz,, $^3J_{2'a,1'b}$= 4.6 Hz, 1 H, 2'-H$_b$), 4.71 (ddt, $^3J_{3,4}$= 9.6 Hz, $^3J_{3,1'b}$= 7.6 Hz, $^3J_{3,1'a}$= 5.2 Hz, 1 H, 3-H), 6.25 (d, $^4J_{5,7}$= 2.3 Hz, 1 H, 5-H), 6.37 (d, $^4J_{7,5}$= 2.3 Hz 1 H, 7-H), 11.24 (s, 1 H, 8$_{OH}$-H).
^{13}C-NMR (CDCl$_3$, 151 MHz): δ [ppm] = -5.4 (Si-(*C*H$_3$)$_2$), 18.3 (*C*(CH$_3$)$_3$), 25.9 (C(*C*H$_3$)$_3$), 33.4 (C-4), 37.8 (C-1'), 55.6 (O*C*H$_3$), 58.5 (C-2'), 75.3 (C-3), 99.7 (C-7), 101.8 (C-8a), 106.2 (C-5), 141.1 (C-4a), 164.6 (C-8), 165.8 (C-6), 169.8 (C-1).
IR (ATR Film): $\tilde{\nu}$ [cm^{-1}] = 2928 (OH, Chelat), 2856, 1667 (C=O), 1628, 1583, 1509, 1464, 1440, 1373 (OH), 1311, 1247 (C-O), 1204, 1156, 1089, 1069, 1035, 1006, 941, 908, 834, 799, 775, 701, 662.
HRMS (ESI, positive ion): Found: 353.1782 ($C_{18}H_{29}O_5Si$) [(M+H)$^+$], Calc.: 353.1779 ($C_{18}H_{29}O_5Si$) [(M+H)$^+$].
$[\alpha]_D^{20}$= +126.0 (c= 1.0, CHCl$_3$)
HPLC (86 %*ee;* Chiralpak IC, heptane/*i*PrOH 80:20, Flowrate = 0.5 mL/min, λ = 217 nm, 265 nm): tR [(*S*)] = 14.8 min, tR [(*R*)] = 16.3 min)
R_f= 0.37 (PE: EtOAc 80:20)
clear oil

Methyl (*E*)-4-((2*R*)-2-(2-((*tert*-butyldimethylsilyl)oxy)ethyl)-6-oxotetrahydro-2*H*-pyran-4-yl)-3-methoxybut-2-enoate

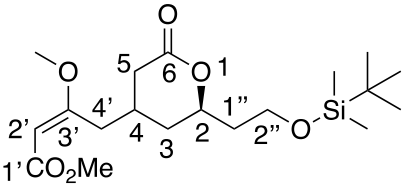

^{1}H-NMR (CDCl$_3$, 600 MHz): δ [ppm] = 0.05 (s, 6 H, Si-(CH$_3$)$_2$), 0.89 (s, 9 H, C-(CH$_3$)$_3$), 1.74 (m, 2 H, 1a''-H, 3-H$_a$), 1.81 (dt, $^4J_{1''b,1''a}$= 15.5 Hz, $^3J_{1''b,2}$= 8.0 Hz, 1 H, 1''-H$_b$), 1.89 (ddt, $^4J_{3b,3a}$= 13.7 Hz, $^3J_{3b,2}$= 9.4 Hz, $^3J_{3b,4}$= 5.2 Hz, 1 H, 3-H$_b$), 2.32 (dd, $^4J_{4'a,4'b}$= 16.2 Hz, $^3J_{4'a,4}$= 9.8 Hz, 1 H, 4'-H$_a$), 2.44 (m, 1 H, 4-H), 2.54 (dd, $^4J_{4'b,4'a}$= 16.2 Hz, $^3J_{4'b,4}$= 5.8 Hz, 1 H, 4'-H$_b$), 2.84 (dd, $^4J_{5a,5b}$= 13.2 Hz, $^3J_{5a,4}$= 6.9 Hz, 1 H, 5-H$_a$), 2.90 (dd, $^4J_{5b,5a}$= 13.2 Hz, $^3J_{5b,4}$= 8.1 Hz, 1 H, 5-H$_b$), 3.63 (s, 3 H, 1'-OCH$_3$), 3.67 (s, 3 H, 3'-

OCH_3), 3.73 (m, 1 H, 2''-H_a), 3.78 (m, 1 H, 2''-Hb), 4.61 (dddd, $^3J_{2,3b}$= 9.4 Hz, $^3J_{2,1''b}$= 8.6 Hz, $^3J_{2,1''a}$= 8.0 Hz, $^3J_{2,3a}$= 4.3 Hz, 1 H, 2-H), 5.10 (s, 1 H, 2'-H).
^{13}C-NMR ($CDCl_3$, 151 MHz): δ [ppm] = -5.4 (Si-(CH_3)$_2$), 18.3 (C-(CH_3)$_3$), 25.9 (C-(CH_3)$_3$), 27.6 (C-4), 33.2 (C-1''), 35.0 (C-4'), 37.2 (C-5), 38.4 (C-3), 51.0 (OCH_3-3'), 55.6 (OCH_3-1'), 58.9 (C-2''), 74.3 (C-2), 92.1 (C-2'), 167.8 (C-3'), 172.4 (C-6), 173.2 (C-1').
IR (ATR Film): $\tilde{\nu}$ [cm^{-1}] = 2929 (OH, Chelat), 2857, 1738, 1711 (lactone), 1622 (C=O), 1463, 1436, 1378, 1287, 1251, 1217, 1192, 1137 (C-O), 1086, 1052, 1007, 931, 833 (RRC=CRH), 775, 733, 663.
HRMS (ESI, positive ion): Found: 387.2202 ($C_{19}H_{35}O_6Si^+$) [(M+H$^+$)], Calc.: 387.2197 ($C_{19}H_{35}O_6Si^+$) [(M+H$^+$)].
clear oil
R_f= 0.19 (PE:EtOAc 80:20)

7-Hydroxy-9-methoxy-6*H*-benzo[*c*]chromen-6-one (21)

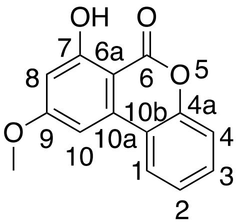

^{1}H-NMR ($CDCl_3$, 600 MHz): δ [ppm] = 3.95 (s, 3 H, 9-OCH_3), 6.60 (d, $^4J_{8,10}$= 2.2 Hz, 1 H, 8-H), 7.08 (d, $^4J_{10,8}$= 2.2 Hz, 1 H, 10-H), 7.36 (m, 2 H, 2-H, 3-H), 7.50 (ddd, $^3J_{1,2}$= 8.5 Hz, $^4J_{1,3}$= 7.3 Hz, $^5J_{1,4}$= 1.5 Hz, 1 H, 1-H), 7.97 (dd, $^3J_{4,3}$= 8.1 Hz, $^5J_{4,1}$= 1.5 Hz, 1 H, 4-H), 11.55 (s, 1 H, 7-OH).
^{13}C-NMR ($CDCl_3$, 151 MHz): δ [ppm] = 55.8 (OCH_3-9), 99.7 (C-10), 100.9 (C-8), 117.8 (C-2/3), 118.2 (C-10b), 123.3 (C-4), 125.0 (C-2/3), 130.8 (C-1), 136.5 (C-10b), 150.8 (C-4a), 164.8 (C-7), 165.2 (C-), 166.9 (C-9).
IR (ATR Film): $\tilde{\nu}$ [cm^{-1}] = 3457 (OH), 3017 (OH, Chelat), 2971, 2948, 2138, 1977, 1739 (lactone), 1659 (C=O), 1626, 1611, 1570, 1515, 1448, 1421, 1367 (OH), 1326, 1285, 1229 (C-O), 1217, 1162, 1107, 1032, 994, 968, 946, 908, 837 (RRC=CRH), 829, 791, 757, 741, 716.
HRMS (ESI, positive ion): Found: 243.0653 ($C_{14}H_{11}O_4^+$) [(M+H$^+$)], Calc.: 243.0652 ($C_{14}H_{11}O_4^+$) [(M+H$^+$)].
white solid
R_f= 0.34 (PE:EtOAc 80:20)
melting point 117 °C

Methyl (*E*)-3-methoxy-4-(2-oxochroman-4-yl)but-2-enoate

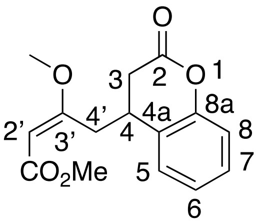

^{1}H-NMR (CDCl$_3$, 600 MHz): δ [ppm] = 2.79 (m, 2 H, 4’-H), 2.99 (dd, $^4J_{3a,3b}$= 13.2 Hz, $^3J_{3a,4}$= 9.0 Hz, 1 H, 3-H$_a$), 3.12 (dd, $^4J_{3b,3a}$= 13.2 Hz, $^3J_{3b,4}$= 6.3 Hz, 1 H, 3-H$_b$), 3.46 (ddt, $^3J_{4,3a}$= 9.0 Hz, $^3J_{4,3b}$= 6.3 Hz, $^3J_{4,4'}$= 5.4 Hz, 1 H, 4-H), 3.60 (s, 3 H, 1’-OCH$_3$), 3.66 (s, 3 H, 3’-OCH$_3$, 5.10 (s, 1 H, 2’-H), 7.05 (m, 1 H, 5-H), 7.10 (dt, $^3J_{4,3a}$= 7.5 Hz, $^3J_{4,3a}$= 1.2 Hz, 1 H, 8-H), 7.26 (m, 2 H, 6-H, 7-H).
^{13}C-NMR (CDCl$_3$, 151 MHz): δ [ppm] = 33.5 (C-4), 34.0 (C-4’), 36.8 (C-3), 51.0 (O*C*H$_3$-3’), 55.5 (O*C*H$_3$-1’), 92.4 (C-2’), 117.0 (C-5), 124.2 (C-8), 125.6 (C-4a), 127.5 (C-6), 128.5 (C-7), 151.4 (C-8a), 167.6 (C-3’), 167.9 (C-2), 172.5 (C-1’).
IR (ATR Film): $\tilde{\nu}$ [cm^{-1}] = 2949 (OH, Chelat), 1770 (lactone), 1707, 1621 (C=O), 1489, 1456, 1436, 1376, 1356, 1283, 1216, 1192, 1132 (C-O), 1110, 1006, 87, 929, 911, 886, 823 (RRC=CRH), 756.
HRMS (ESI, positive ion): Found: 277.1073 ($C_{15}H_{17}O_5^+$) [(M+H$^+$)], Calc.: 277.1071 ($C_{15}H_{17}O_5^+$) [(M+H$^+$)].
clear oil
R$_f$= 0.25 (PE:EtOAc 80:20)

7-Hydroxy-3,9-dimethoxy-6*H*-benzo[*c*]chromen-6-one (23)

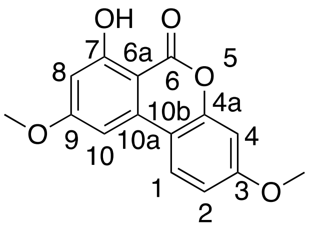

^{1}H-NMR (CDCl$_3$, 600 MHz): δ [ppm] = 3.89 (s, 3 H, OCH$_3$), 3.93 (s, 3 H, OCH$_3$), 6.53 (d, $^4J_{8,10}$= 2.2 Hz, 1 H, 8-H), 6.85 (d, $^4J_{8,10}$= 2.5 Hz, 1 H, 1-H), 6.92 (dd, $^3J_{2,1}$= 8.8 Hz, $^4J_{2,4}$= 2.5 Hz, 1 H, 2-H), 6.94 (d, $^4J_{8,10}$= 2.2 Hz, 1 H, 10-H), 7.84 (d, $^4J_{4,2}$= 8.8 Hz, 1 H, 4-H), 11.48 (s, 1 H, 7$_{OH}$-H).
^{13}C-NMR (CDCl$_3$, 151 MHz): δ [ppm] = 55.7 (C-O*C*H$_3$), 55.8 (C-O*C*H$_3$), 98.7 (C-8), 99.9 (C-10a), 101.5 (C-4), 113.0 (C-10b), 124.4 (C-2), 161.6 (C-7), 164.7 (C-9), 167.0 (C-6).
IR (ATR Film): $\tilde{\nu}$ [cm^{-1}] = 3084, 2990, 2971, 2949 (OH, Chelat), 2853, 1737 (lactonee), 1666, 1607 (C=O), 1572, 1491, 1467, 1456, 1435, 1376, 1352, 1266, 1232 (C-O), 1206, 1180, 1168 (C-O-C), 1146, 1092, 1035, 981, 957, 872, 850, 829, 796, 782, 759, 718, 688.
HRMS (ESI, positive ion): Found: 273.0758 ($C_{15}H_{13}O_5$) [(M+H)$^+$], Calc.: 273.0757 ($C_{15}H_{13}O_5$) [(M+H)$^+$].
R$_f$= 0.24 (PE:e 80:20)
white solid
melting point: 132 °C

Methyl (*E*)-3-methoxy-4-(7-methoxy-2-oxochroman-4-yl)but-2-enoate

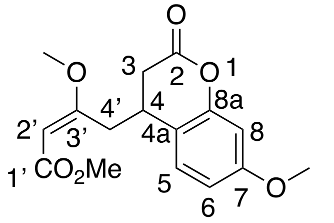

^{1}H-NMR (CDCl$_3$, 600 MHz): δ [ppm] = 2.76 (m, 2 H, 4'-H), 2.95 (ddt, $^{2}J_{3a,3b}$= 13.2 Hz, $^{3}J_{3a,4}$= 8.8 Hz, $^{3}J_{3a,4'}$= 1.1 Hz, 1 H, 3-H$_a$), 3.08 (ddt, $^{2}J_{3b,3a}$= 13.2 Hz, $^{3}J_{3b,4}$= 6.2 Hz, $^{3}J_{3b,4'}$= 1.2 Hz, 1 H, 3-H$_b$), 3.40 (m, 1 H, 4-H), 3.60 (s, 3 H, 1'-OCH$_3$), 3.66 (s, 3 H, 3'-OCH$_3$), 3.79 (s, 3 H, 7-OCH$_3$), 5.09 (s, 1 H, 2'-H), 6.60 (d, $^{4}J_{8,6}$= 2.4 Hz, 1 H, 8-H), 6.66 (d, $^{3}J_{5,6}$= 8.4 Hz, 1 H, 5-H), 7.14 (dd, $^{3}J_{6,5}$= 8.4 Hz, $^{4}J_{6,8}$= 2.4 Hz, 1 H, 6-H).
^{13}C-NMR (CDCl$_3$, 151 MHz): δ [ppm] = 32.9 (C-4), 34.9 (C-4'), 37.1 (C-3), 51.0 (O*C*H$_3$-3'), 55.5 (O*C*H$_3$-1'), 55.5 (O*C*H$_3$-7), 92.3 (C-2'), 102.4 (C-8), 110.5 (C-5), 117.5 (C-8a), 128-0 (C-6), 152.2 (C-4a), 159.8 (C-7), 167.6 (C-3'), 167.9 (C-2), 172.2 (C-1').
IR (ATR Film): ṽ [cm^{-1}] = 2971, 2949 (OH, Chelat), 2841, 1767 (lactone), 1738, 1709 (C=O), 1619, 1588, 1508, 1435, 1374, 1353, 1269, 1231, 1218, 1175, 1132 (C-O-C), 1050, 1033. 972, 929, 890, 820 (RRC=CRH), 730.
HRMS (ESI, positive ion): Found: 307.1180 ($C_{16}H_{19}O_6$) [(M-CH$_3$+H$^+$)], Calc.: 307.1176 ($C_{16}H_{19}O_6$) [(M-CH$_3$+H$^+$)], Found: 233.0812 ($C_{13}H_{13}O_4^{\bullet}$) Calc.: 233.0814 ($C_{13}H_{13}O_4^{\bullet}$).
R_f= 0.20 (PE:EtOAc 80:20)
clear oil

7-Hydroxy-3,9-dimethoxy-1-methyl-6*H*-benzo[*c*]chromen-6-one (24)

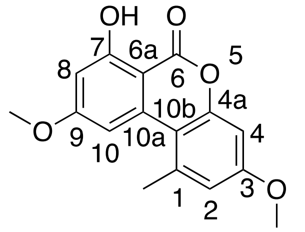

^{1}H-NMR (CDCl$_3$, 600 MHz): δ [ppm] = 2.81 (s, 3 H, 1-CH$_3$), 3.86 (s, 3 H, OCH$_3$), 3.92 (s, 3 H, OCH$_3$), 6.55 (d, $^{4}J_{8,10}$= 2.3 Hz, 1 H, 8-H), 6.76 (m, 2 H, 2-H, 4-H), 7.27 (d, $^{4}J_{10,8}$= 2.3 Hz, 1 H, 10-H), 11.96 (s, 1 H, 7$_{OH}$-H).
^{13}C-NMR (CDCl$_3$, 151 MHz): δ [ppm] = 25.7 (C-*C*H$_3$), 55.6 (C-O*C*H$_3$), 55.7 (C-O*C*H$_3$), 98.9 (C-8), 99.4 (C-10a), 100.0 (C-4), 104.6 (C-10), 110.9 (C-10b), 116.9 (C-2), 138.0 (C-3), 138.2 (C-1), 153.2 (C-10b), 160.0 (C-4a), 165.2 (C-7), 165.5 (C-9), 166.4 (C-6).
IR (ATR Film): ṽ [cm^{-1}] = 3092, 2938 (OH, Chelat), 2847, 1660 (C=O), 1607, 1584, 1458, 1408, 1348 (OH), 1283, 1258, 1233 (C-O), 1199 1174, 1157, 1110, 1055, 1030, 983, 952, 870, 848, 830, 799, 779, 690, 642, 579, 515.
HRMS (ESI, positive ion): Found: 287.0913 ($C_{16}H_{15}O_5$) [(M+H)$^+$], Calc.: 287.0914 ($C_{16}H_{15}O_5$) [(M+H)$^+$].
R_f= 0.42 (PE:EE 80:20)
melting point: 177 °C; colourless solid

Methyl (*E*)-3-methoxy-4-(7-methoxy-5-methyl-2-oxochroman-4-yl)but-2-enoate

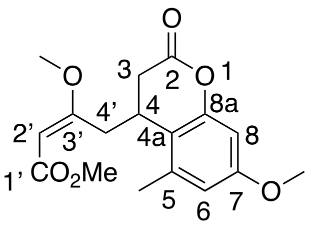

^{1}H-NMR ($CDCl_3$, 600 MHz): δ [ppm] = 2.37 (s, 3 H, 5-CH_3), 2.67 (dd, $^2J_{3a,3b}$= 16.3 Hz, $^3J_{3a,4}$= 6.3 Hz, 1 H, 3-H_a), 2.82 (dd, $^2J_{3b,3a}$= 16.3 Hz, $^3J_{3b,4}$= 1.7 Hz, 1 H, 3-H_b), 2.98 (m, 2 H, 4'-H), 3.51 (tdd, $^3J_{4,4'}$= 8.2 Hz, $^3J_{4,3a}$= 6.2 Hz, $^3J_{4,3b}$= 1.7 Hz, 1 H, 4-H), 3.62 (s, 3 H, 1'-OCH_3), 3.65 (s, 3 H, 3'-OCH_3), 3.77 (s, 3 H, 7-OCH_3), 5.08 (s, 1 H, 2'-H), 6.46 (d, $^4J_{8,6}$= 2.5 Hz, 1 H, 8-H), 6.53 (d, $^3J_{6,8}$= 2.5 Hz, 1 H, 6-H).
^{13}C-NMR ($CDCl_3$, 151 MHz): δ [ppm] = 18.8 (*C*H_3-5), 30.4 (C-4), 34.0 (C-3), 35.5 (C-4'), 51.0 (O*C*H_3-3'), 55.4 (O*C*H_3-1'), 55.4 (O*C*H_3-7), 92.6 (C-2'), 100.3 (C-8), 112.4 (C-6), 116.4 (C-5), 137.3 (C-4a), 152.5 (C-8a), 159.2 (C-7), 167.5 (C-3'), 167.9 (C-2), 172.0 (C-1').
IR (ATR Film): $\tilde{\nu}$ [cm^{-1}] = 2953 (OH, Chelat), 2853, 1771 (lactone), 1697 (C=O), 1619, 1579, 1424, 1278, 1178, 1132 (C-O-C), 1043, 822 (RRC=CRH).
HRMS (ESI, positive ion): Found: 191.0703 ($C_{11}H_{11}O_3^•$) [$(M-C_7H_{11}O_3^•)^•$], Calc.: 191.0708 ($C_{11}H_{11}O_3^•$) [$(M-C_7H_{11}O_3^•)^•$], Found: 247.0965 ($C_{14}H_{15}O_4^•$) [$(M-CH_3-C_3H_5O_2^•)^•$], Calc.: 247.0970 ($C_{14}H_{15}O_4^•$) [$(M-CH_3-C_3H_5O_2^•)^•$], Found: 289.1072 ($C_{16}H_{17}O_5^•$) [$(M-CH_3O^•-CH_3)^•$], Calc.: 289.1076 ($C_{16}H_{17}O_5^•$) [$(M-CH_3O^•-CH_3)^•$], Found: 321.1333 ($C_{17}H_{21}O_6^+$) [$(M-CH_3+H^+)$], Calc.: 321.1333 ($C_{17}H_{21}O_6^+$) [$(M-CH_3+H^+)$], Found: 338.1600 ($C_{17}H_{24}NO_6^+$) [$(M-CH_3+NH_4^+)$], Calc.: 338.1598 ($C_{17}H_{24}NO_6^+$) [$(M-CH_3+NH_4^+)$], Found: 353.1594 ($C_{18}H_{25}O_7^+$) [$(M-CH_3+MeOH+H^+)$], Calc.: 353.1595 ($C_{18}H_{25}O_7^+$) [$(M-CH_3+MeOH+H^+)$], Found: 375.1415 ($C_{18}H_{25}NaO_7^+$) [$(M-CH_3+MeOH+Na^+)$], Calc.: 375.1414 ($C_{18}H_{25}NaO_7^+$) [$(M-CH_3+MeOH+Na^+)$].

R_f= 0.20 (PE:EtOAc 80:20)

7-Hydroxy-1,9-dimethoxy-3-methyl-6*H*-benzo[*c*]chromen-6-one (27)

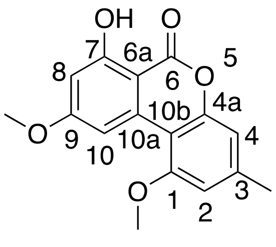

^{1}H-NMR ($CDCl_3$, 600 MHz): δ [ppm] = 2.43 (s, 3 H, 3-CH_3), 3.91 (s, 3 H, OCH_3), 4.02 (s, 3 H, OCH_3), 6.55 (d, $^4J_{8,10}$= 2.3 Hz, 1 H, 8-H), 6.67 (s, 1 H, 2/4-H), 6.81 (s, 1 H, 2/4-H), 8.02 (d, $^4J_{10,8}$= 2.3 Hz, 1 H, 10-H), 11.84 (s, 1 H, 7-OH).

^{13}C-NMR ($CDCl_3$, 151 MHz): δ [ppm] = 21.8 (*C*H_3-3), 55.6 (O*C*H_3-9), 56.0 (O*C*H_3-1), 99.8 (C-8), 105.6 (C-10), 106.0 (C-6a), 106.4 (C-10b), 108.4 (C-2), 110.6 (C-4), 113.3 (C-10a), 141.2 (C-3), 152.0 (C-4a), 155.8 (C-9), 158.3 (C-1), 164.6 (C-7), 166.7 (C-6).
IR (ATR Film): $\tilde{\nu}$ [cm^{-1}] = 3010, 2936 (OH, Chelat), 2838, 1725 (lactone), 1653, 1615 (C=O), 1461 (OH), 1294, 1262, 1222 (C-O), 1201, 1099, 1041, 985, 855, 821, 753, 686, 641, 616, 585, 521.
HRMS (ESI, positive ion): Found: 287.0912 ($C_{16}H_{15}O_5$) [$(M+H)^+$], Calc.: 287.0914 ($C_{16}H_{15}O_5$) [$(M+H)^+$].
R_f= 0.35 (PE: EtOAc 80:20)
white solid
melting point: 188 °C

Methyl (*E*)-3-methoxy-4-(5-methoxy-7-methyl-2-oxochroman-4-yl)but-2-enoate

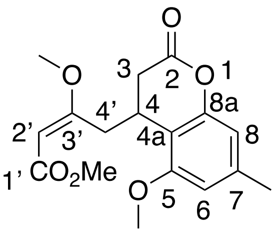

^{1}H-NMR ($CDCl_3$, 600 MHz): δ [ppm] = 2.32 (s, 3 H, 7-CH_3), 2.66 (dd, $^2J_{3a,3b}$= 16.4 Hz, $^3J_{3a,4}$= 7.1 Hz, 1 H, 3-H_a), 2.83 (m, 2 H, 3-Hb, 4'-H_a), 3.23 (dd, $^2J_{4'b4'a}$= 13.6 Hz, $^3J_{4'b,4}$= 7.7 Hz, 1 H, 4'-H_b), 3.56 (s, 3 H, 1'-CH_3), 3.60 (s, 3 H, 3'-CH_3), 3.70 (m, 1 H, 4-H), 3.83 (s, 3 H, 5-OCH_3), 5.01 (s, 1 H, 2'-H), 6.44 (s, 1 H, 6-H), 6.48 (s, 1 H, 8-H).
^{13}C-NMR ($CDCl_3$, 151 MHz): δ [ppm] = 21.7 (*C*H_3-7), 27.8 (C-4), 33.8 (C-3), 35.2 (C-4'), 50.9 (O*C*H_3-3'), 55.2 (O*C*H_3-1'), 55.7 (O*C*H_3-5), 92.6 (C-2'), 107.1 (C-6), 109.8 (C-8), 110.9 (C-4a), 139.0 (C-7), 152.2 (C-8a), 156.5 (C-5), 167.5 (C-3'), 168.0 (C-2), 172.1 (C-1').
IR (ATR Film): $\tilde{\nu}$ [cm^{-1}] = 2953 (OH, Chelat), 2844, 1767 (lactone), 1715, 1697 (C=O), 1615, 1588, 1433, 1160, 1133 (C-O-C), 1102, 1050, 923, 819 (RRC=CRH), 587.
HRMS (ESI, positive ion): Found: 191.0703 ($C_{11}H_{11}O_3^{\bullet}$) [$(M-C_7H_{11}O_3^{\bullet})^{\bullet}$], Calc.: 191.0708 ($C_{11}H_{11}O_3^{\bullet}$) [$(M-C_7H_{11}O_3^{\bullet})^{\bullet}$], Found: 247.0965 ($C_{14}H_{15}O_4^{\bullet}$) [$(M-CH_3-C_3H_5O_2^{\bullet})^{\bullet}$], Calc.: 247.0970 ($C_{14}H_{15}O_4^{\bullet}$) [$(M-CH_3-C_3H_5O_2^{\bullet})^{\bullet}$], Found: 289.1072 ($C_{16}H_{17}O_5^{\bullet}$) [$(M-CH_3O^{\bullet}-CH_3)^{\bullet}$], Calc.: 289.1076 ($C_{16}H_{17}O_5^{\bullet}$) [$(M-CH_3O^{\bullet}-CH_3)^{\bullet}$], Found: 321.1333 ($C_{17}H_{21}O_6^+$) [$(M-CH_3+H^+)$], Calc.: 321.1333 ($C_{17}H_{21}O_6^+$) [$(M-CH_3+H^+)$], Found: 338.1600 ($C_{17}H_{24}NO_6^+$) [$(M-CH_3+NH_4^+)$], Calc.: 338.1598 ($C_{17}H_{24}NO_6^+$) [$(M-CH_3+NH_4^+)$], Found: 370.1859 ($C_{18}H_{28}NO_7^+$) [$(M-CH_3+MeOH+NH_4^+)$], Calc.: 370.1860 ($C_{18}H_{25}NaO_7^+$) [$(M-CH_3+MeOH+NH_4^+)$].
clear oil
R_f= 0.22 (PE:EtOAc 80:20)

4. NMR Kinetics

To slow down the reaction and measuring the proton NMR kinetic, 1 mol% Tf_2CH_2 and 2 mol% $AlMe_3$ and dilution in toluene-d8 have been used.

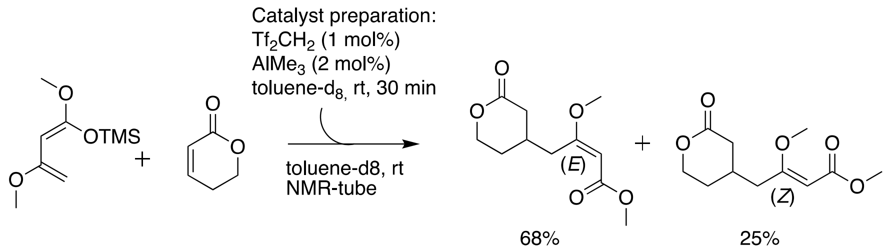

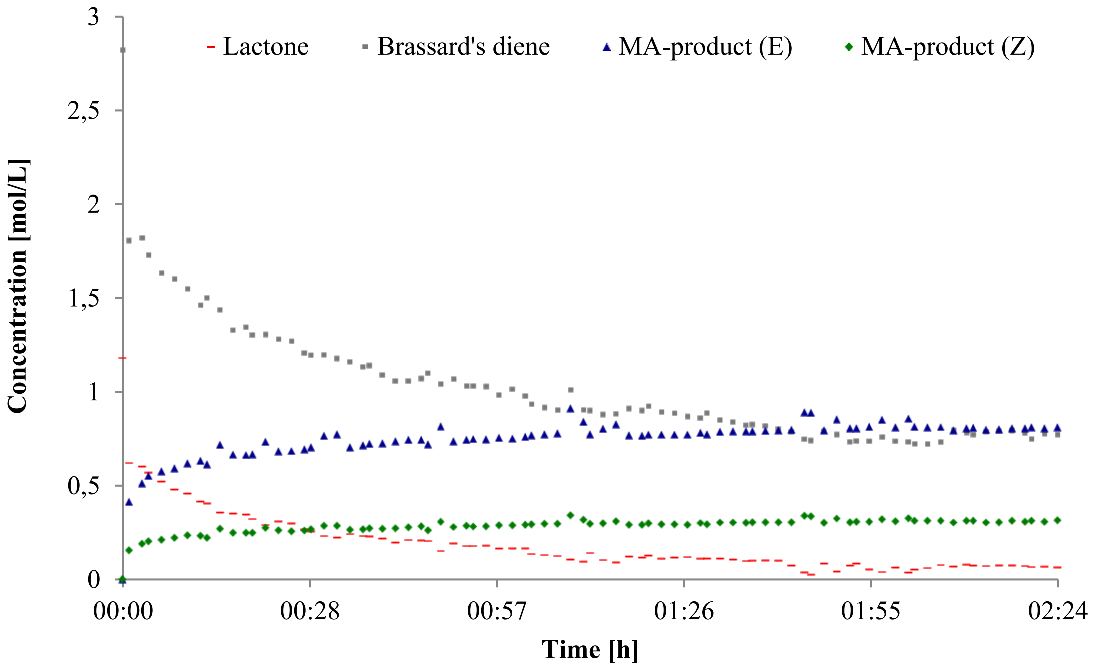

FIGURE 1. NMR kinetics of Brassard's diene and 5,6-dihydro-2*H*-pyran-2-one in toluene-d8.

5. NMR Spectra

8-Hydroxy-6-methoxy-3,4,4a,5-tetrahydro-1*H*-isochromen-1-one (12)

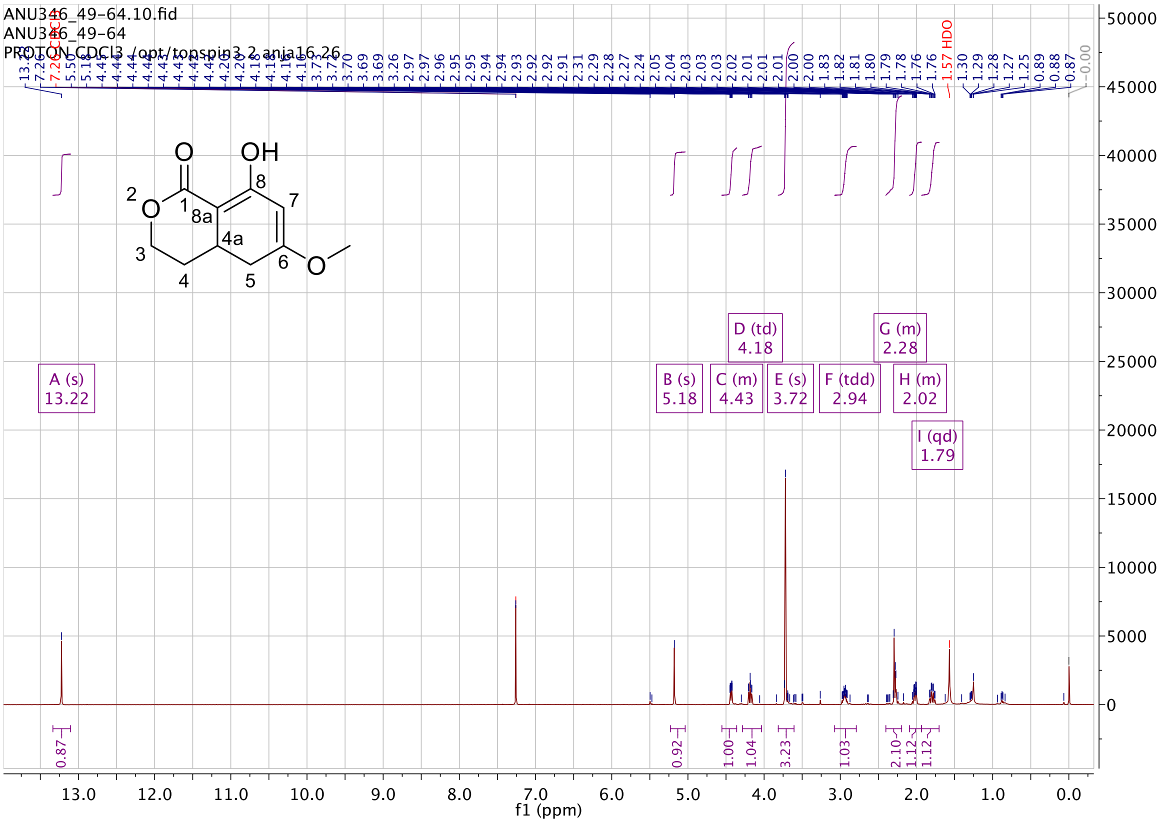

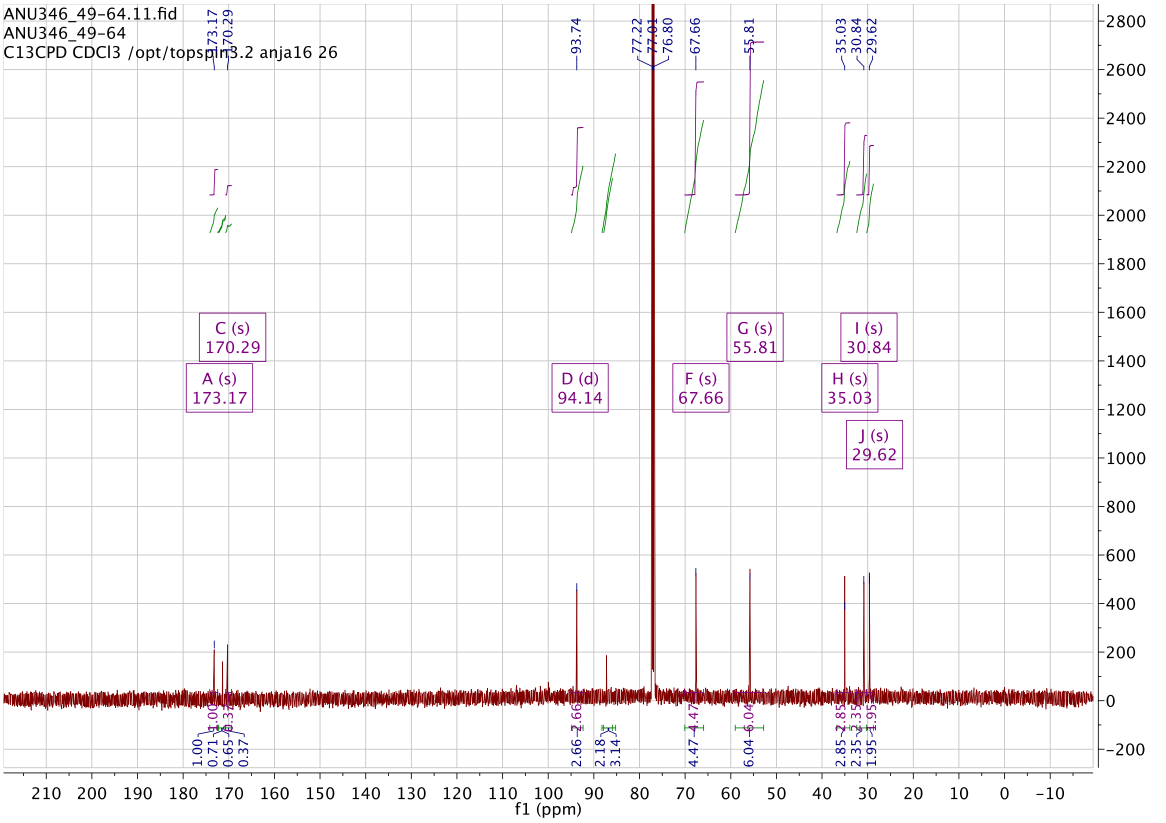

8-Hydroxy-6-methoxyisochroman-1-one (14)

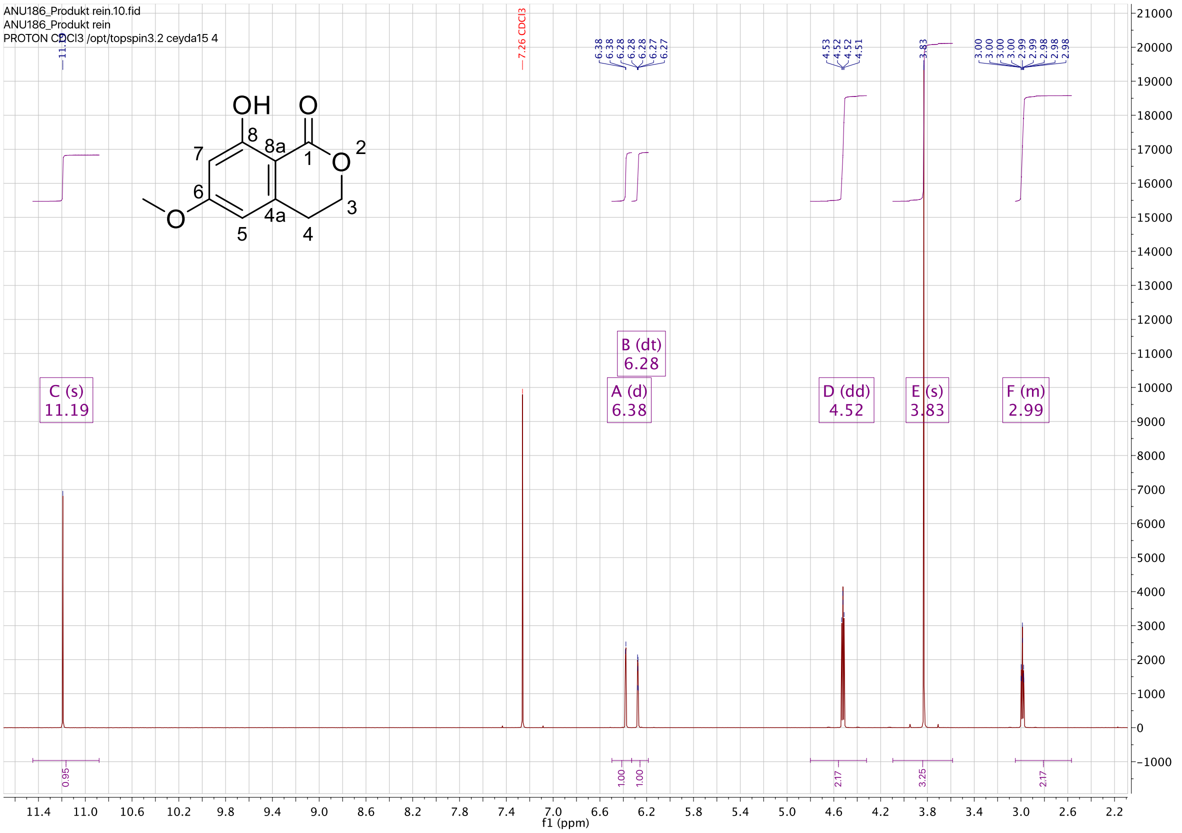

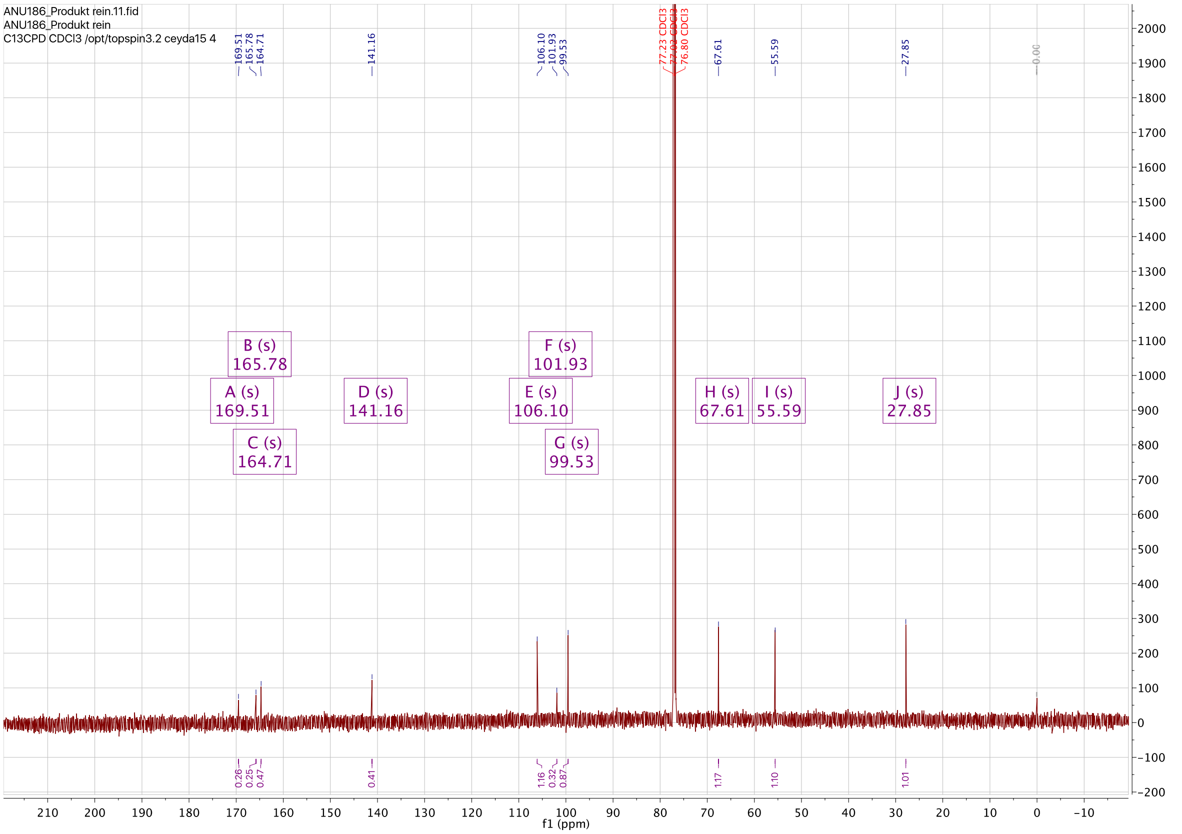

Methyl (*E*)-3-methoxy-4-(2-oxotetrahydro-2*H*-pyran-4-yl)but-2-enoate [(*E*)-13]

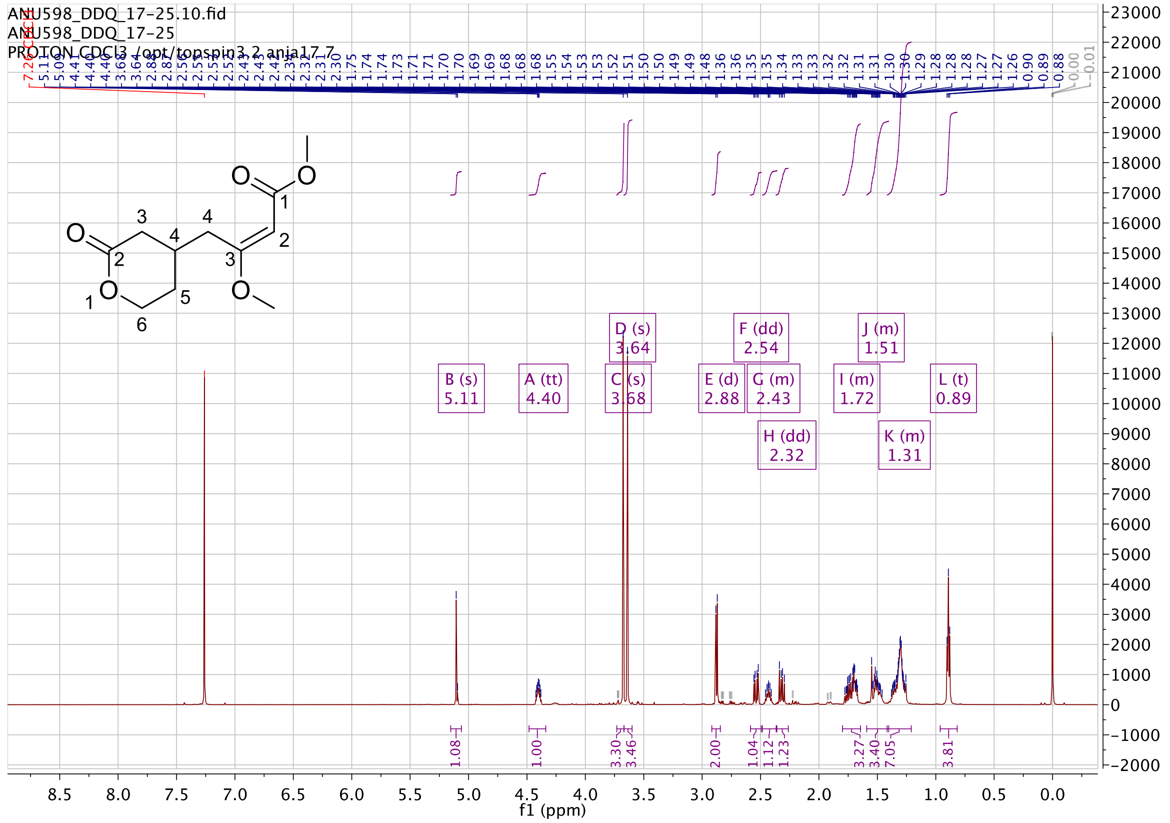

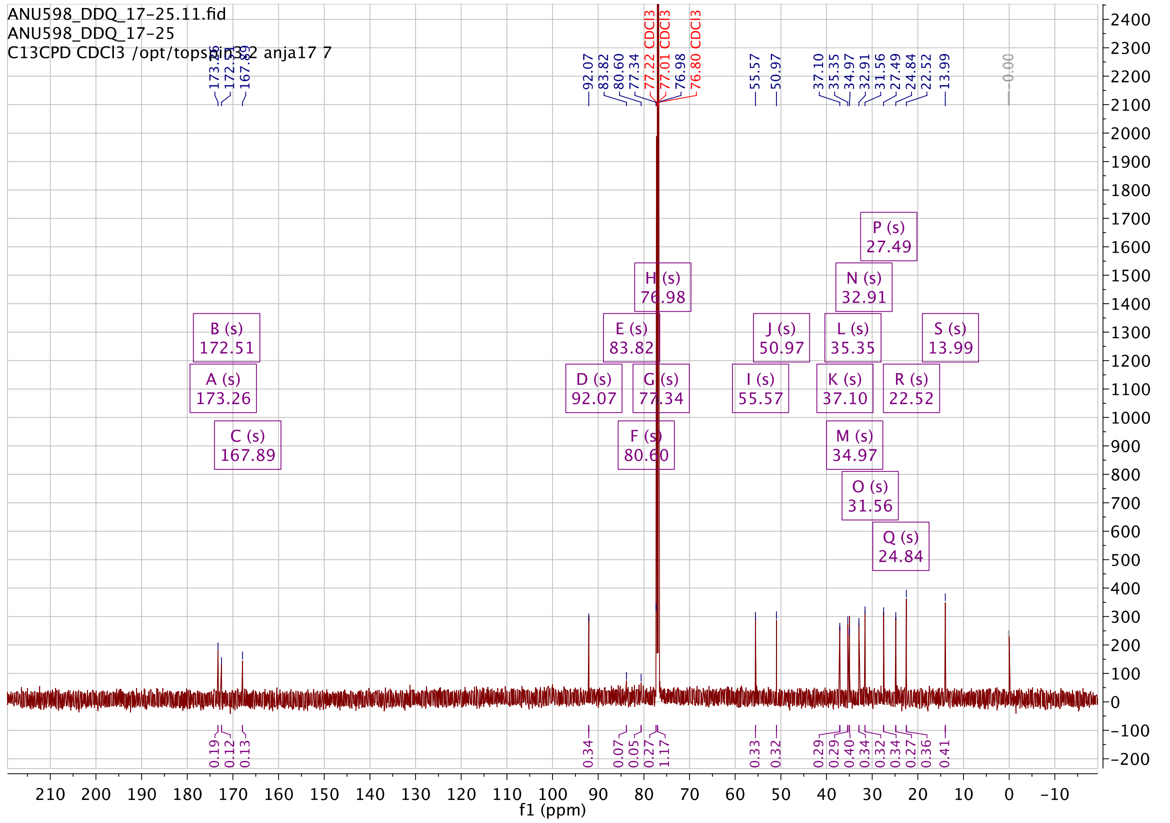

Methyl (*Z*)-3-methoxy-4-(2-oxotetrahydro-2*H*-pyran-4-yl)but-2-enoate [(*Z*)-13]

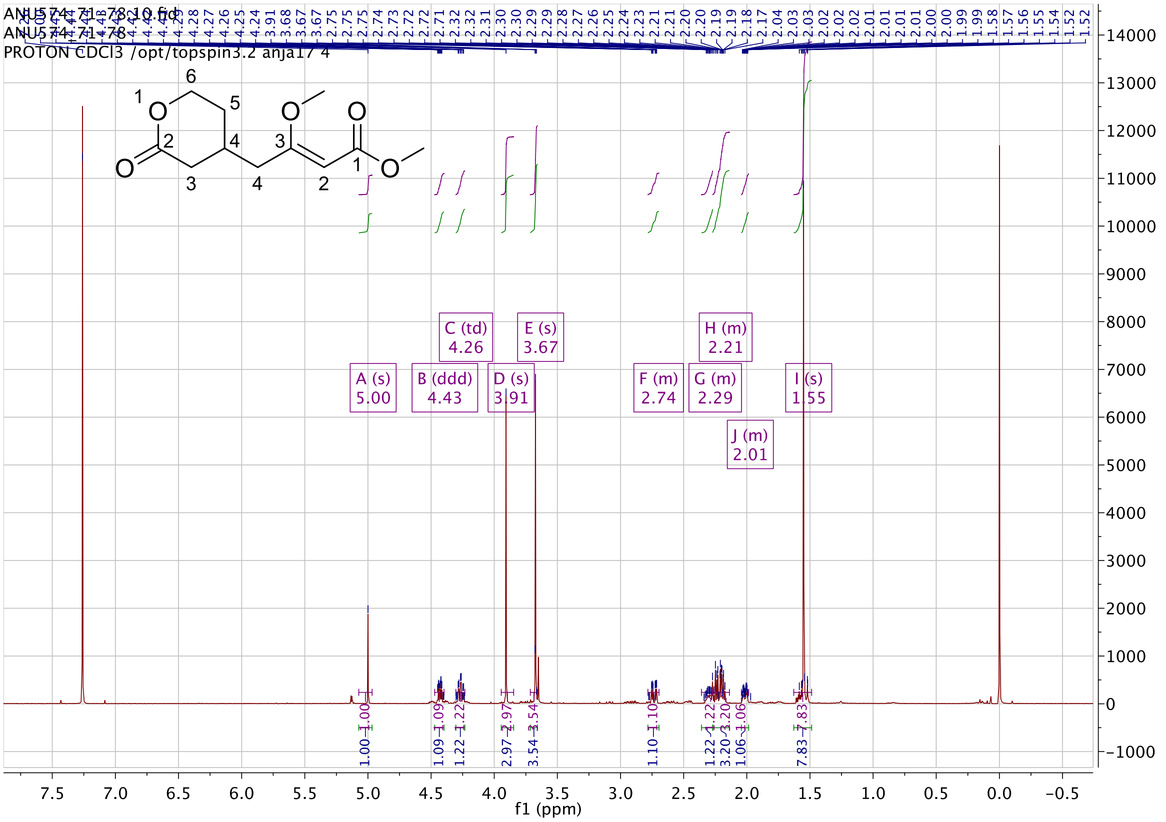

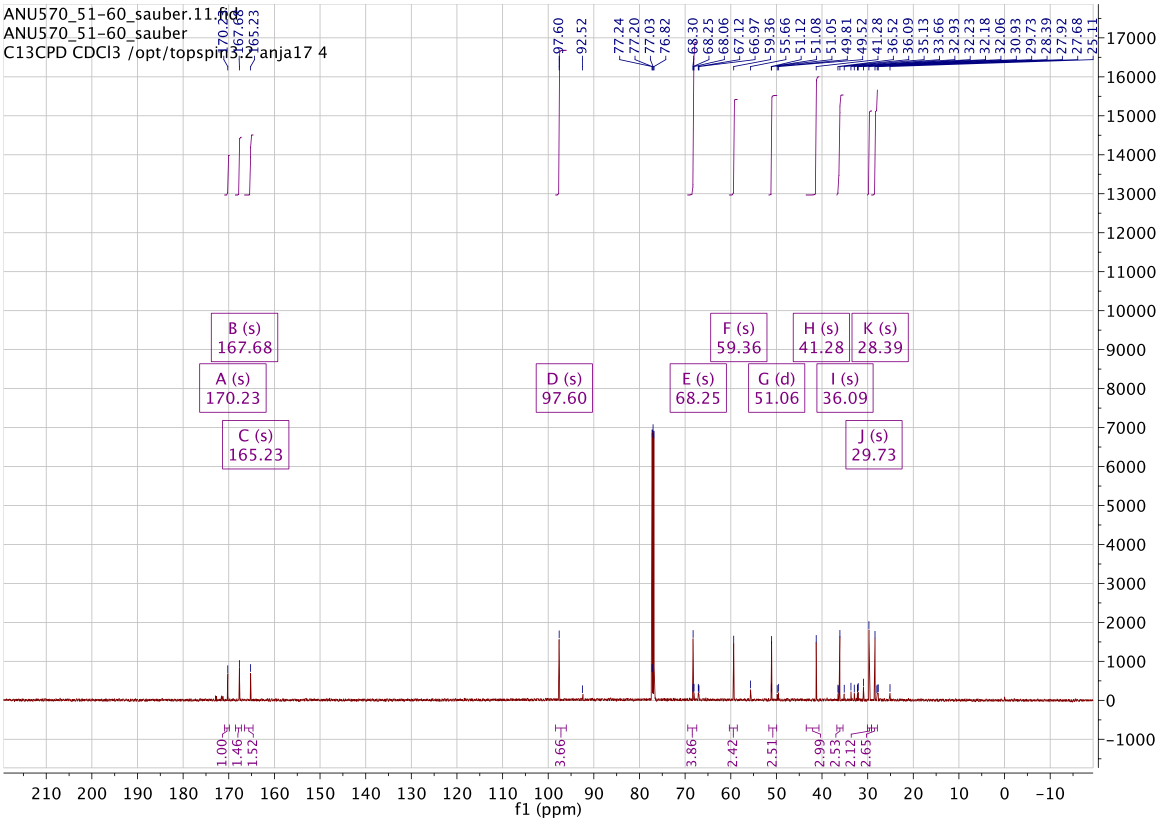

(*R*)-8-Hydroxy-6-methoxy-3-methylisochroman-1-one [(*R*)-3]

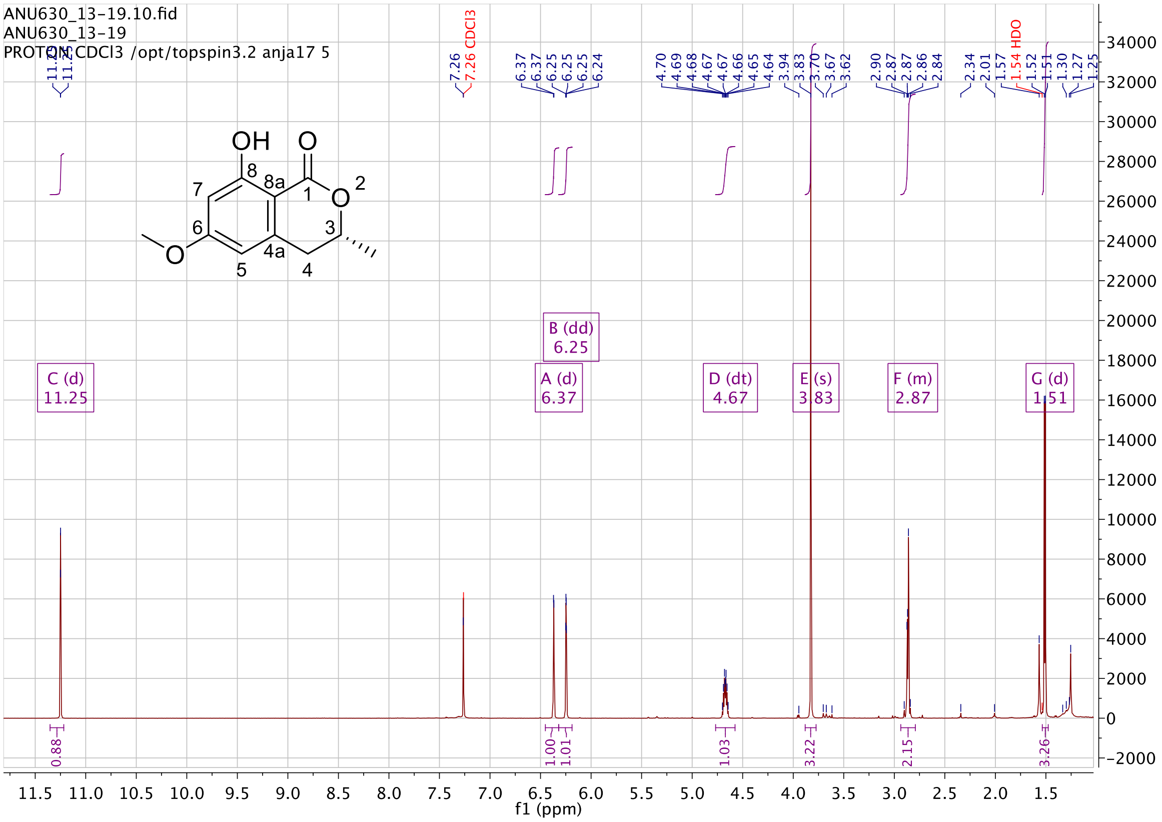

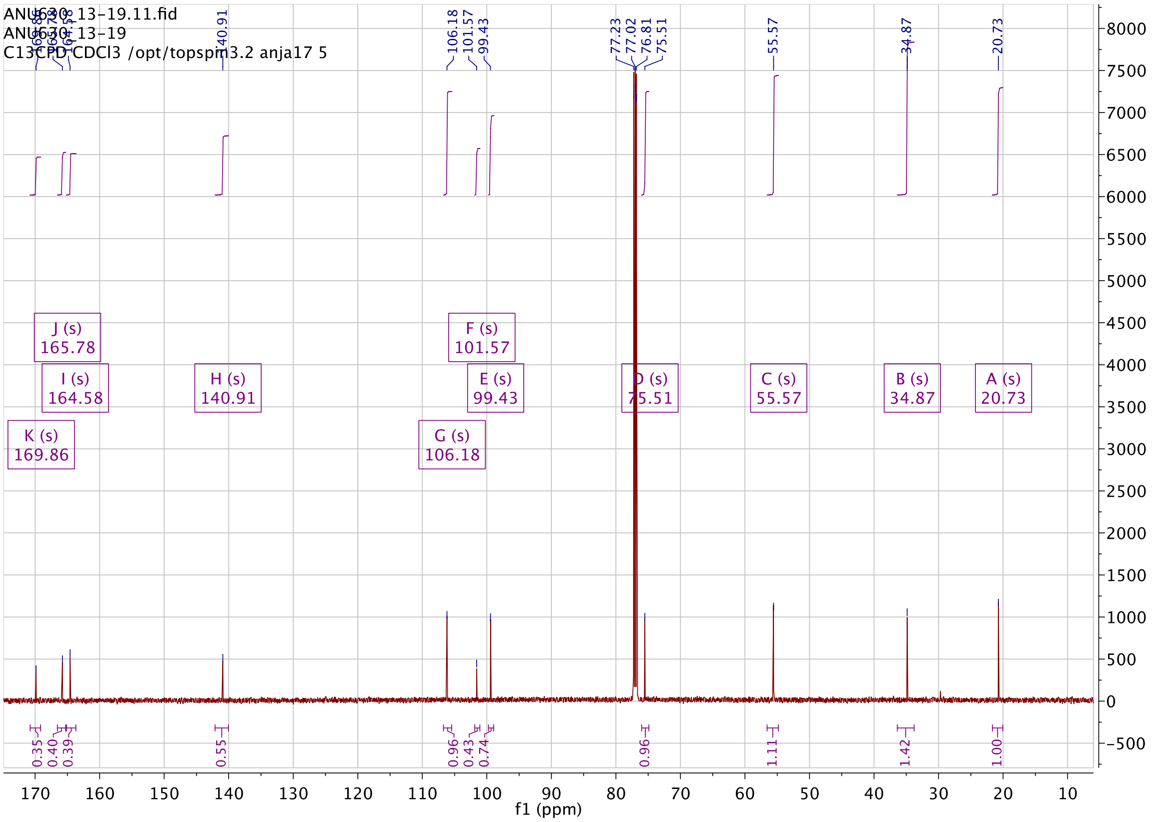

Methyl (*E*)-3-methoxy-4-((2*R*)-2-methyl-6-oxotetrahydro-2*H*-pyran-4-yl)but-2-enoate

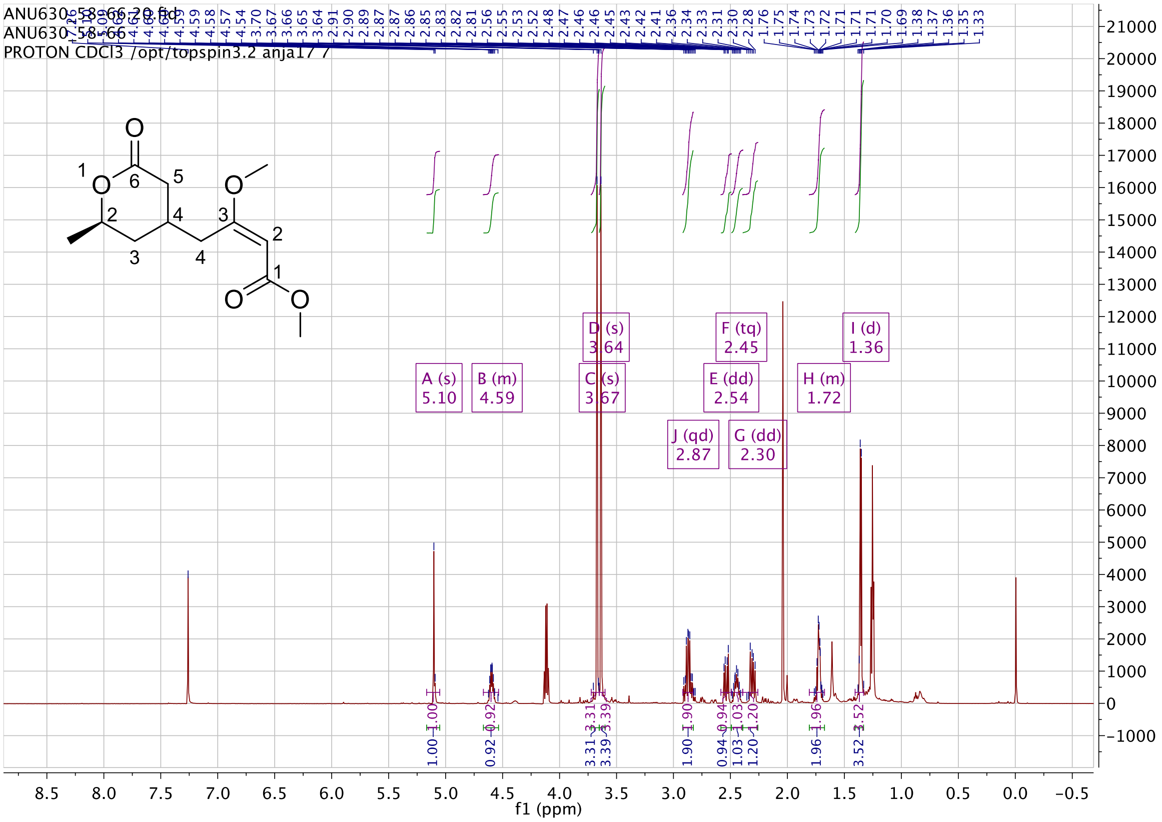

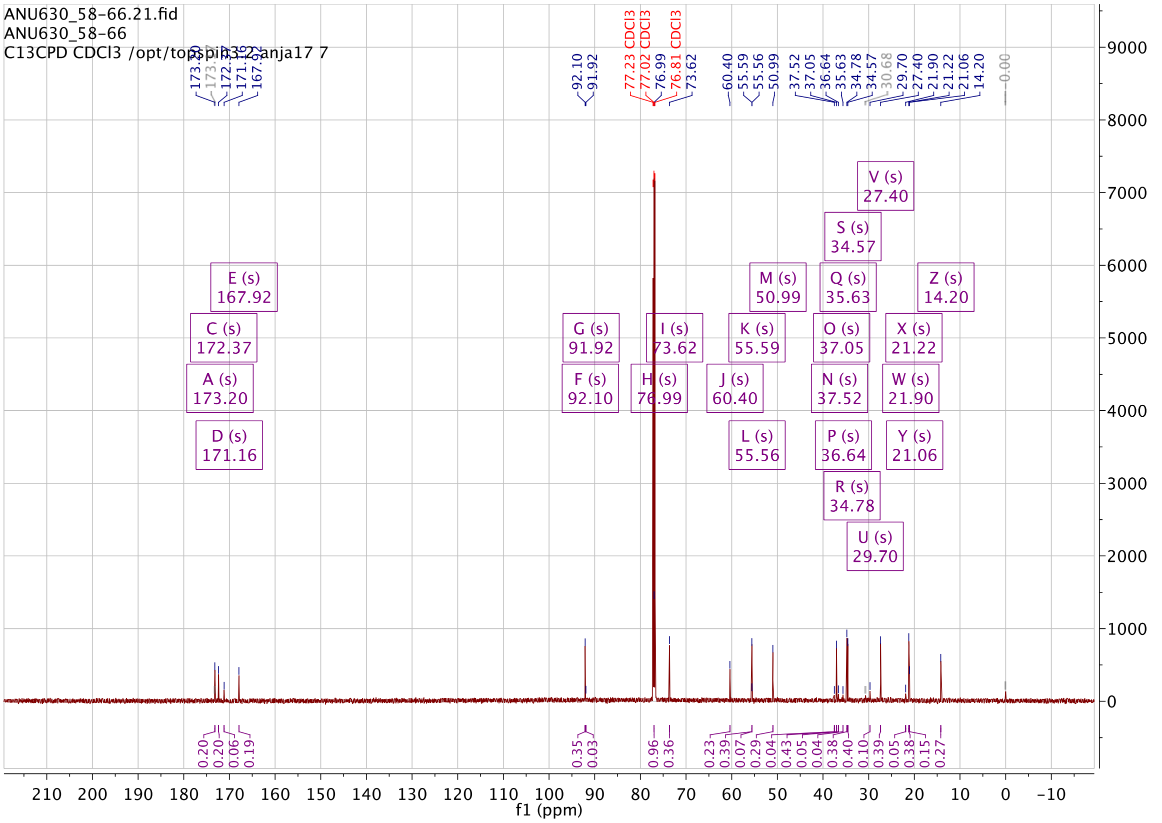

(*S*)-8-Hydroxy-6-methoxy-3-methylisochroman-1-one [(*S*)-4]

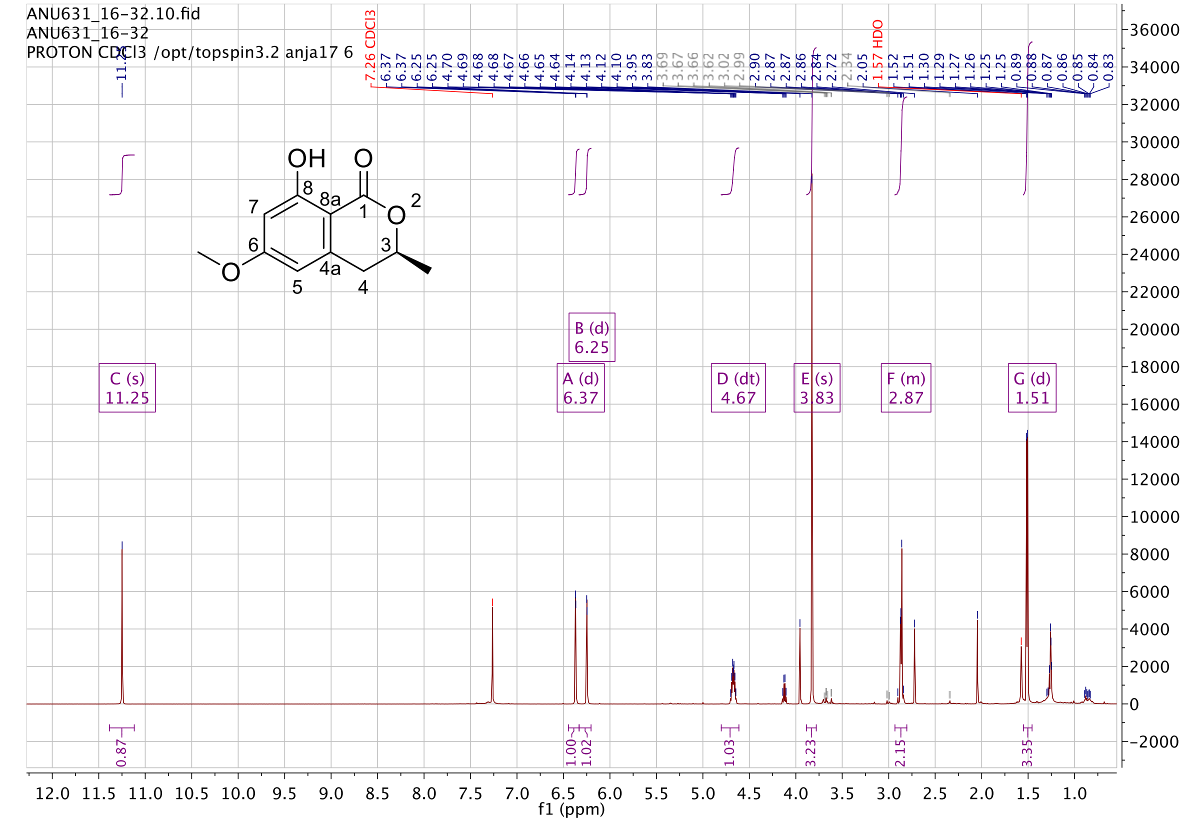

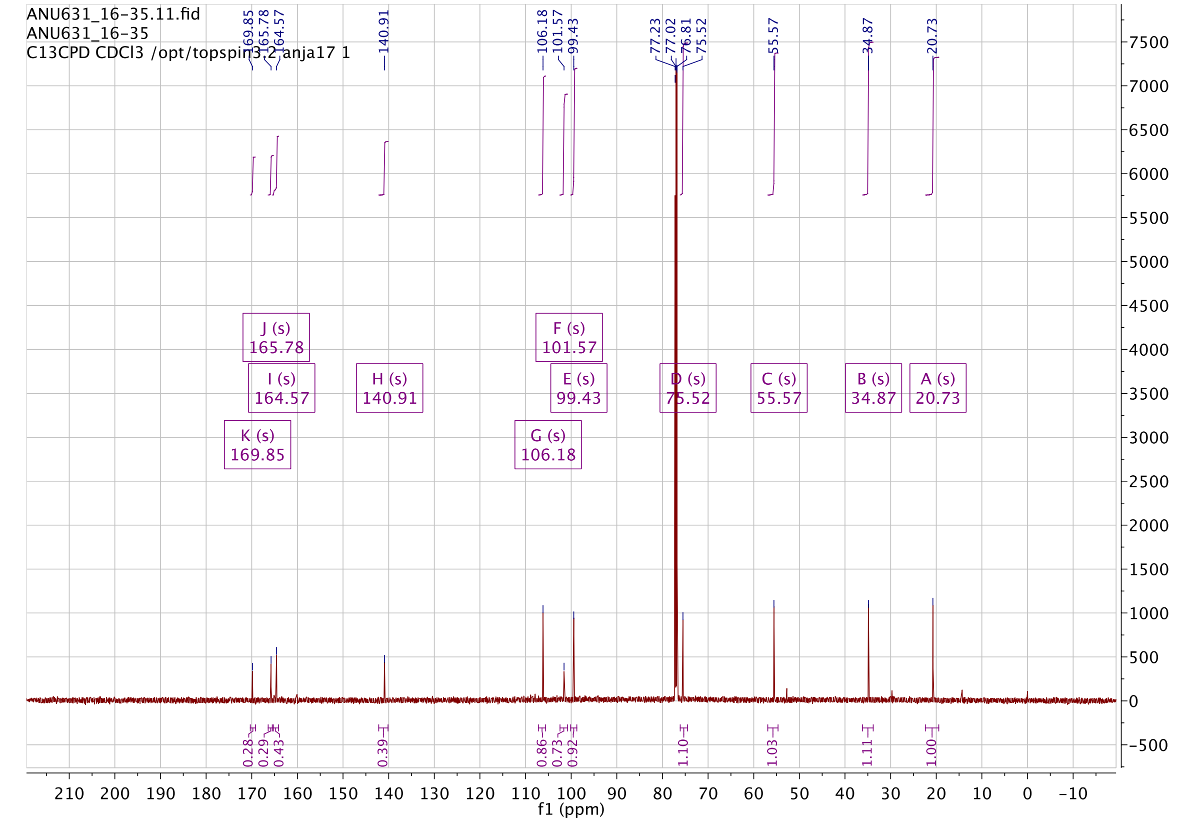

Methyl (*E*)-3-methoxy-4-((2*S*)-2-methyl-6-oxotetrahydro-2*H*-pyran-4-yl)but-2-enoate

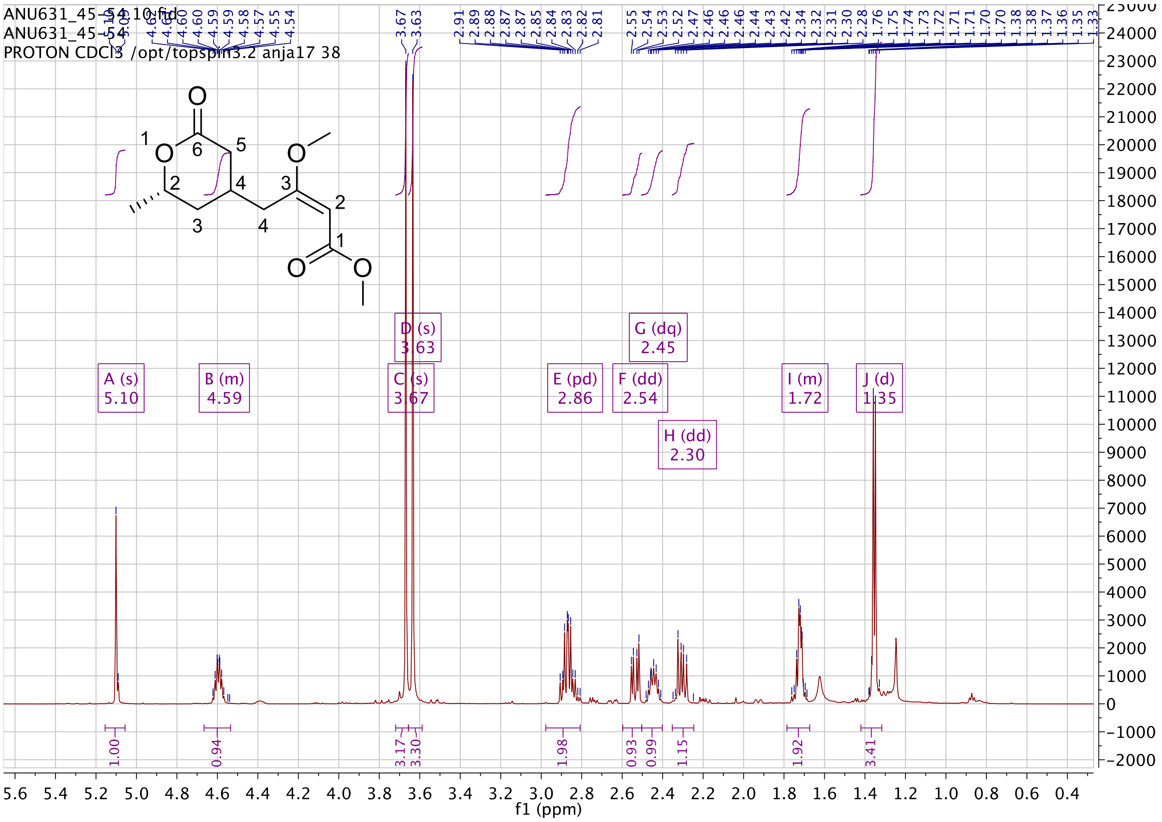

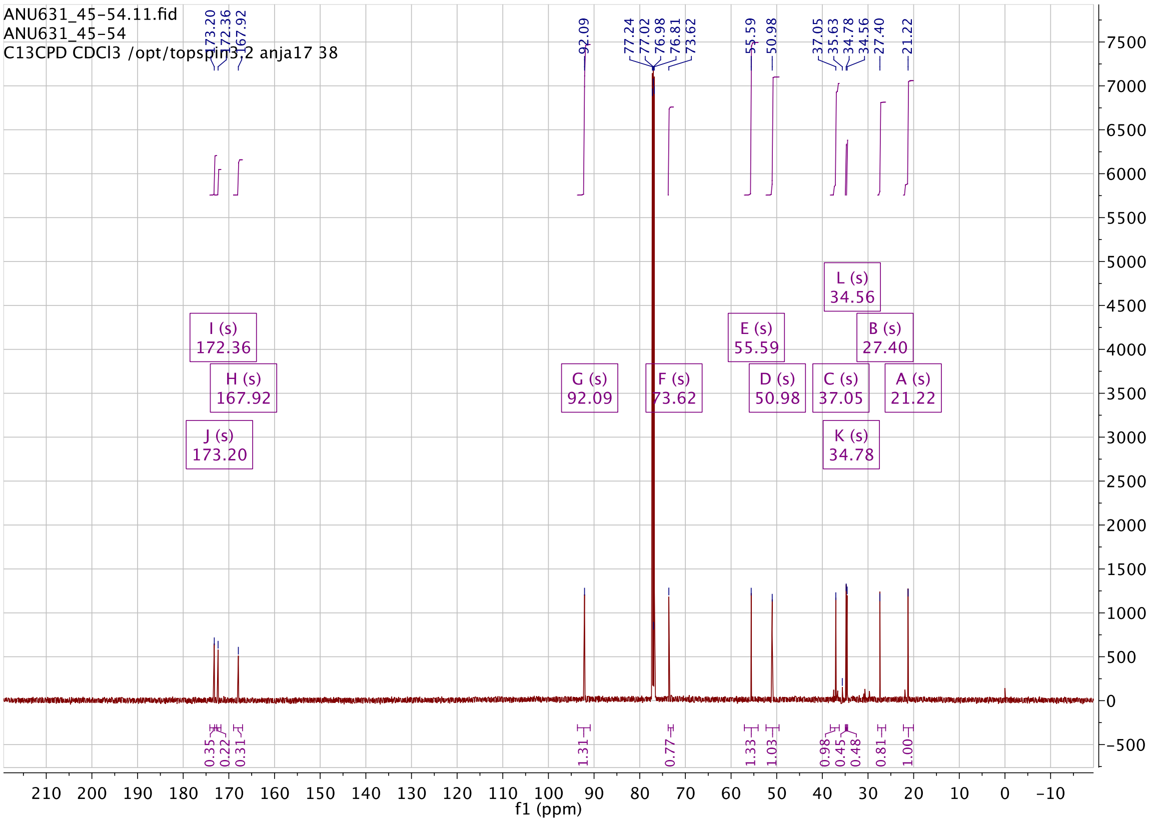

8-Hydroxy-6-methoxy-3-pentylisochroman-1-one (16)

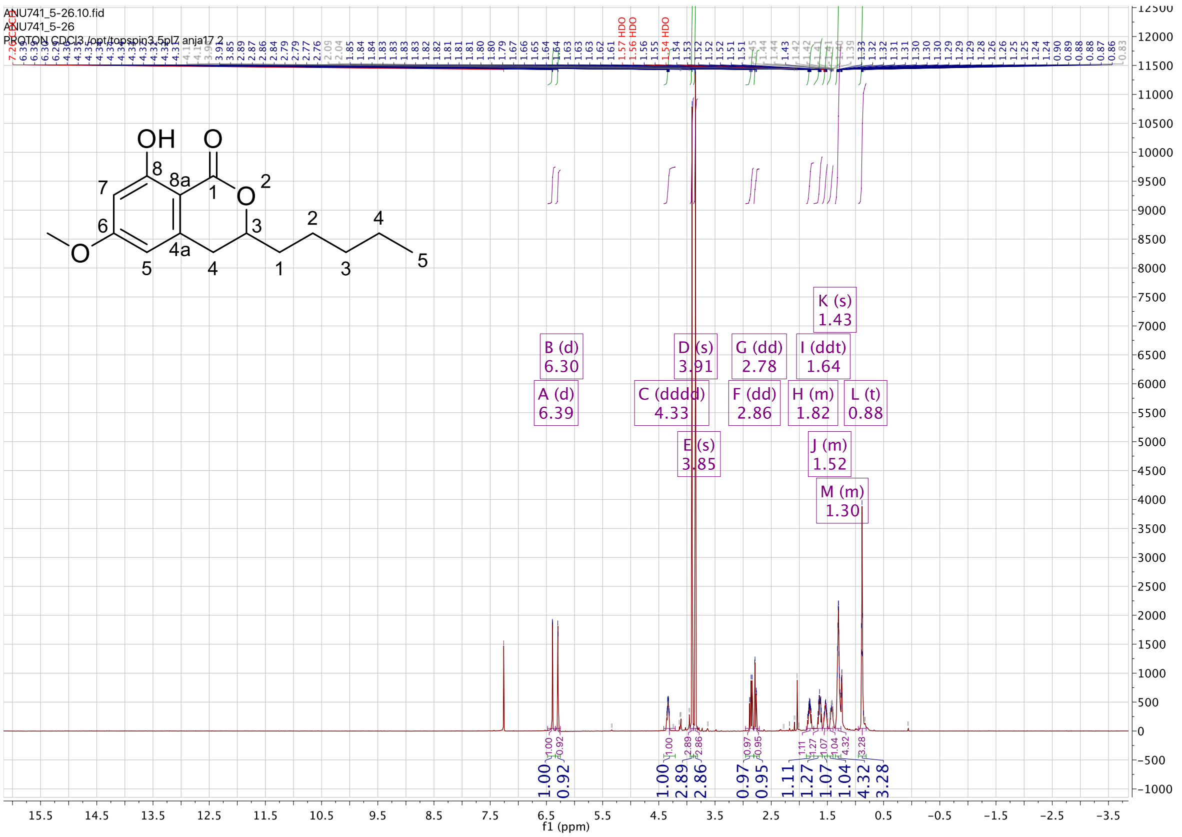

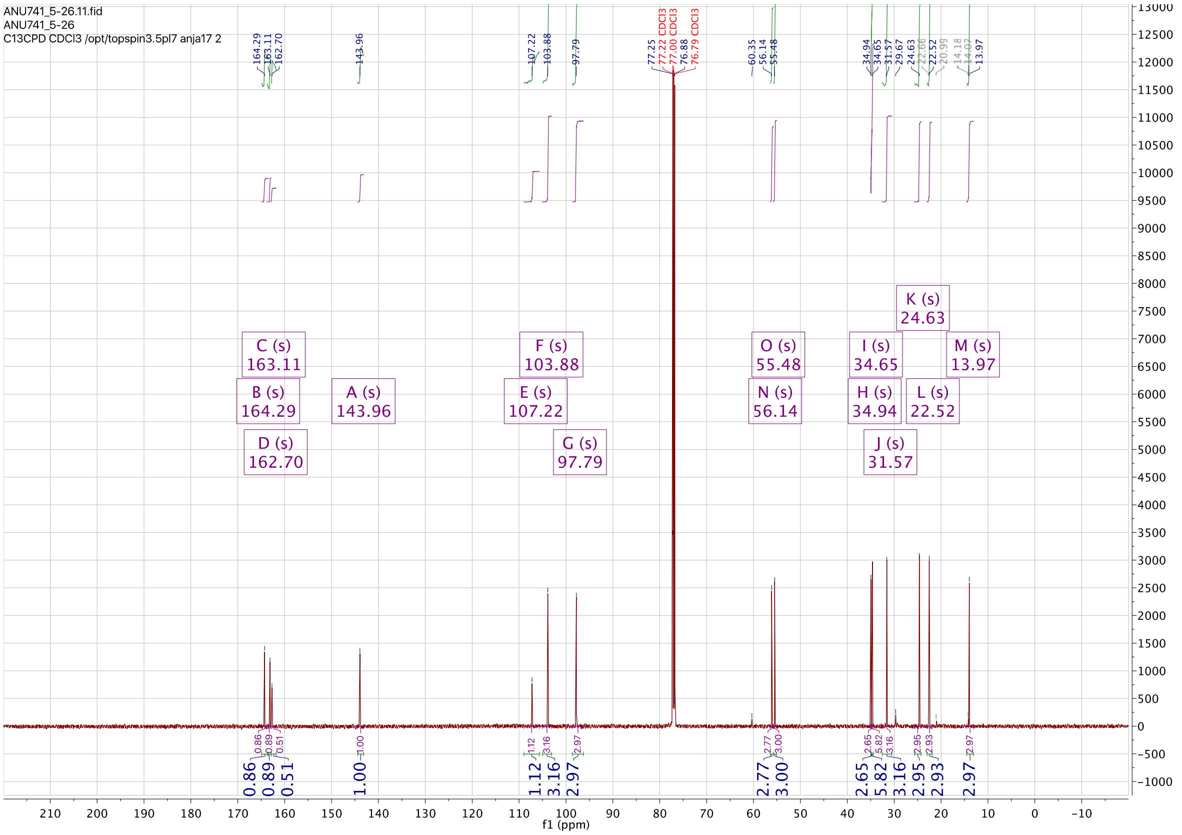

Methyl (*E*)-3-methoxy-4-(2-oxo-6-pentyltetrahydro-2*H*-pyran-4-yl)but-2-enoate

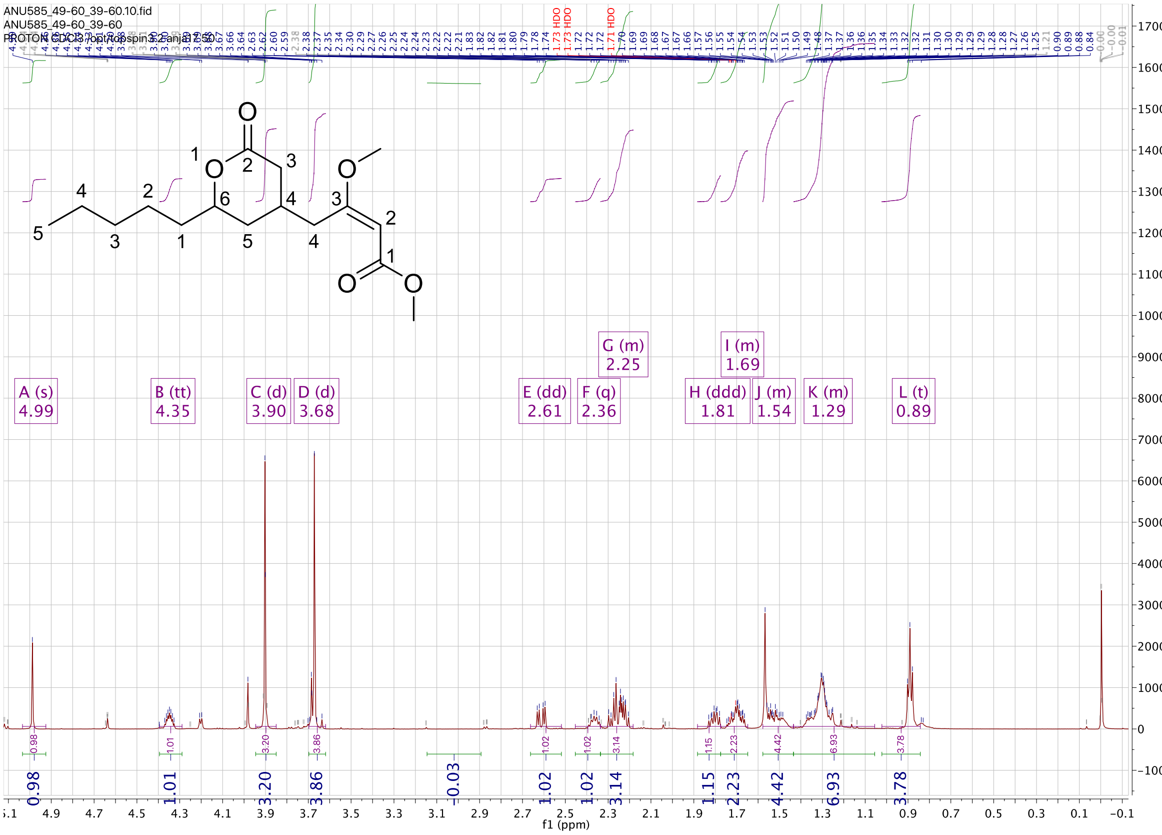

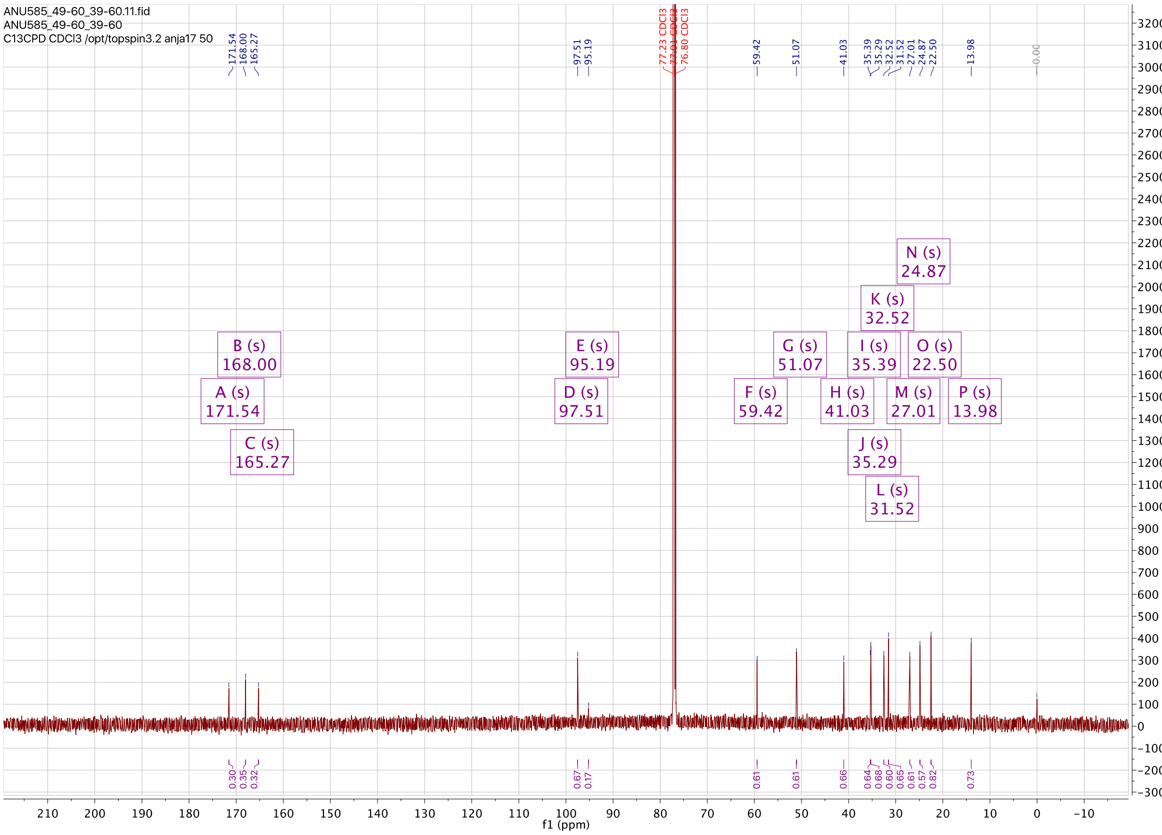

(*S*)-3-(2-((*tert*-Butyldimethylsilyl)oxy)ethyl)-8-hydroxy-6-methoxyisochroman-1-one [(*S*)-18]

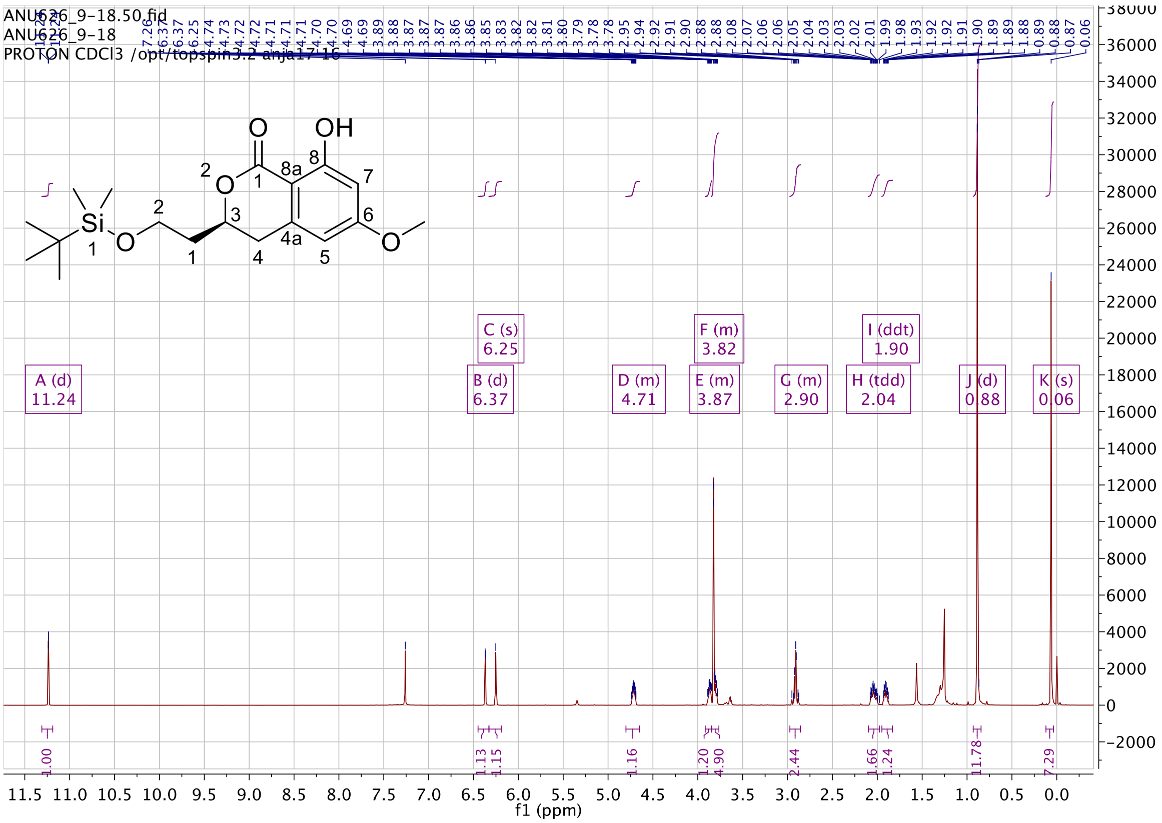

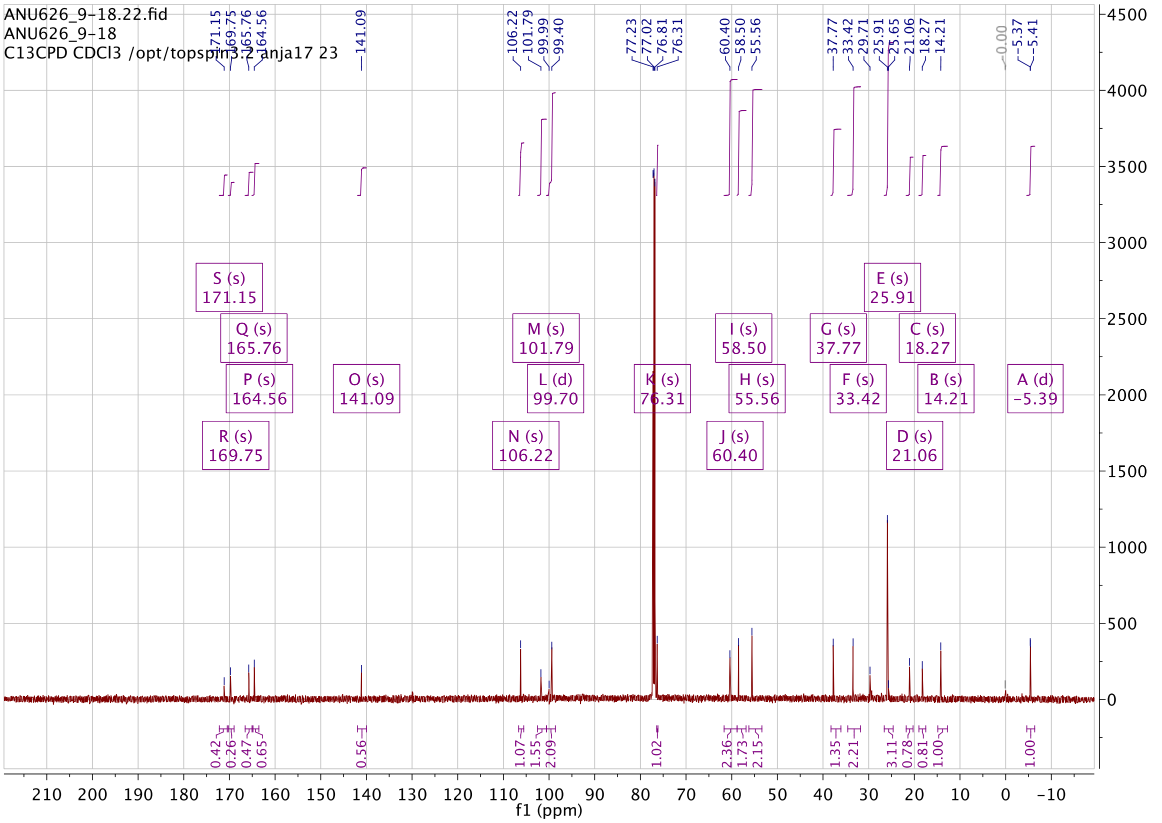

Methyl (*E*)-4-((2*S*)-2-(2-((*tert*-butyldimethylsilyl)oxy)ethyl)-6-oxotetrahydro-2*H*-pyran-4-yl)-3-methoxybut-2-enoate

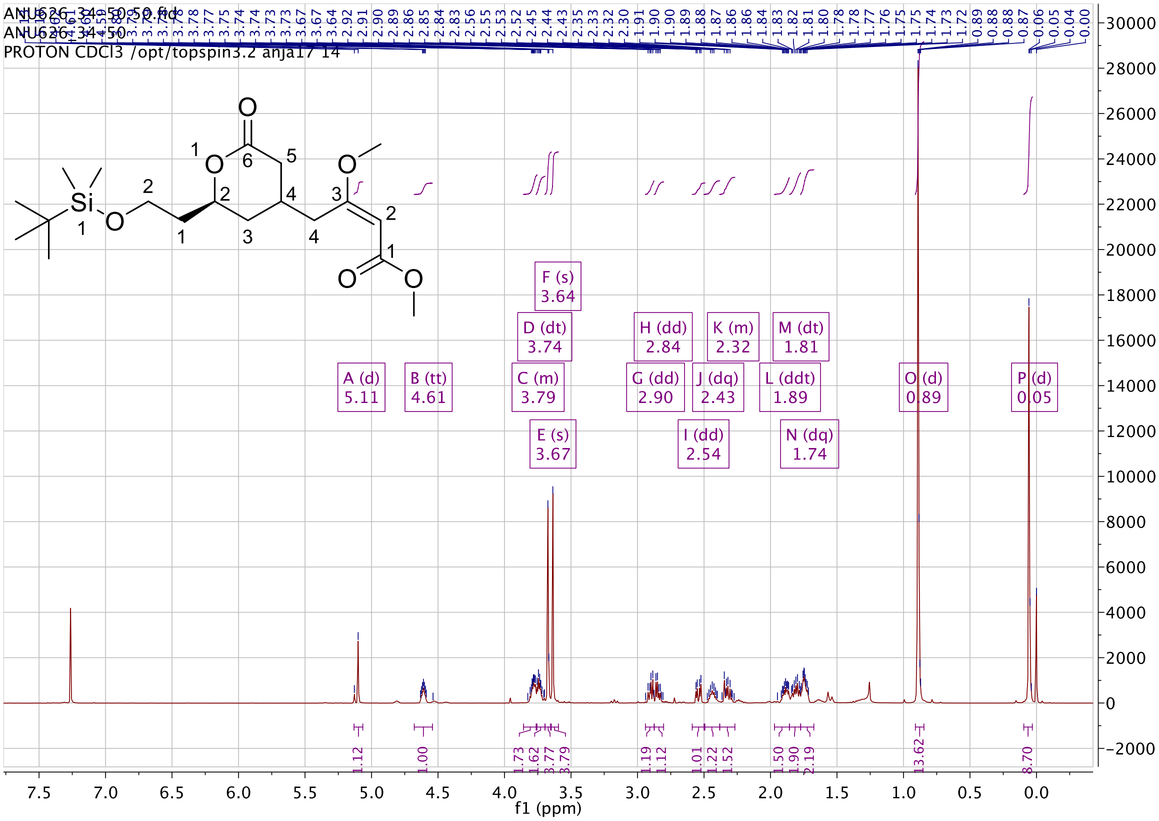

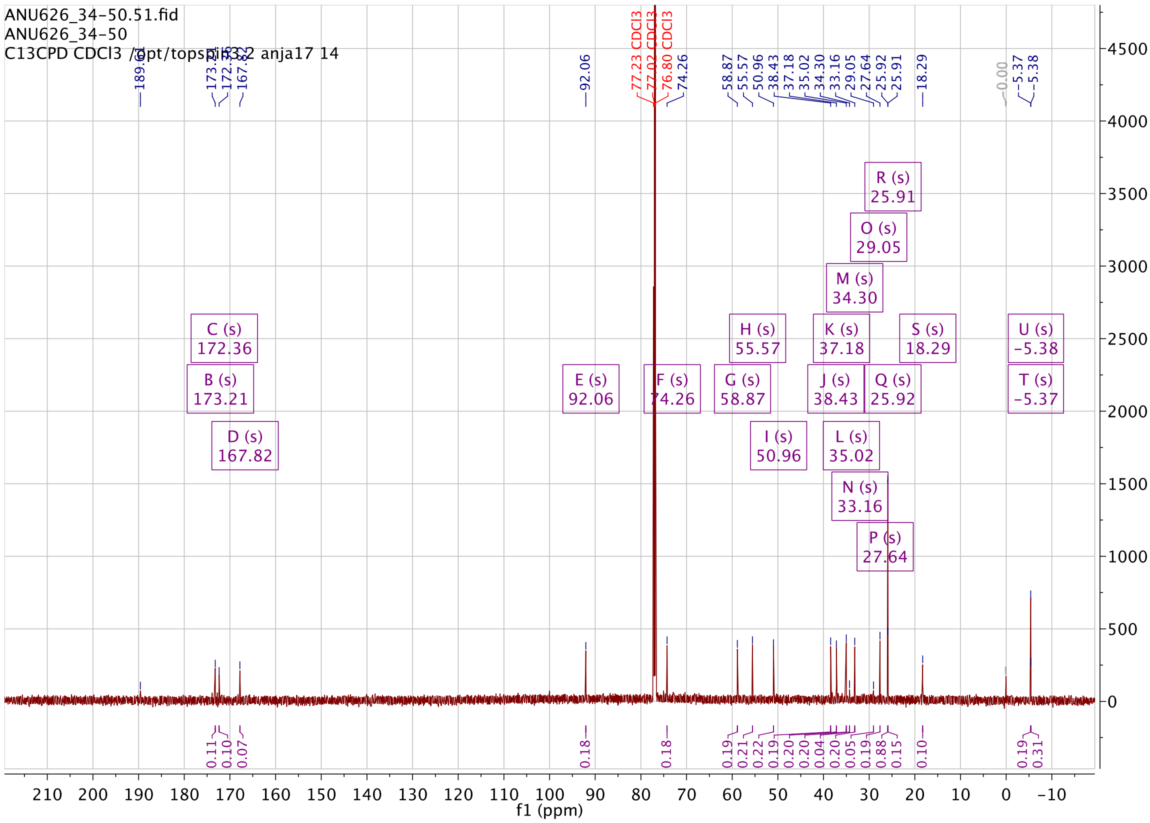

(*R*)-3-(2-((*tert*-butyldimethylsilyl)oxy)ethyl)-8-hydroxy-6-methoxyisochroman-1-one [(*R*)-18]

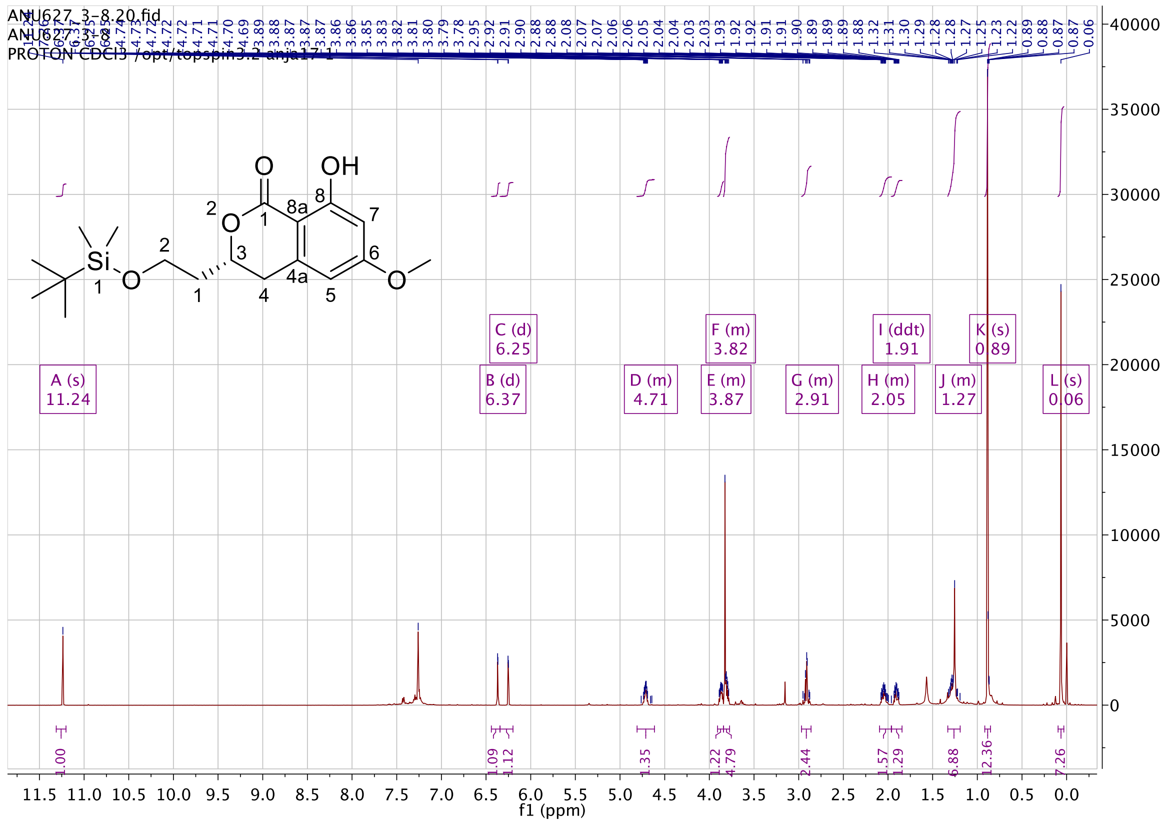

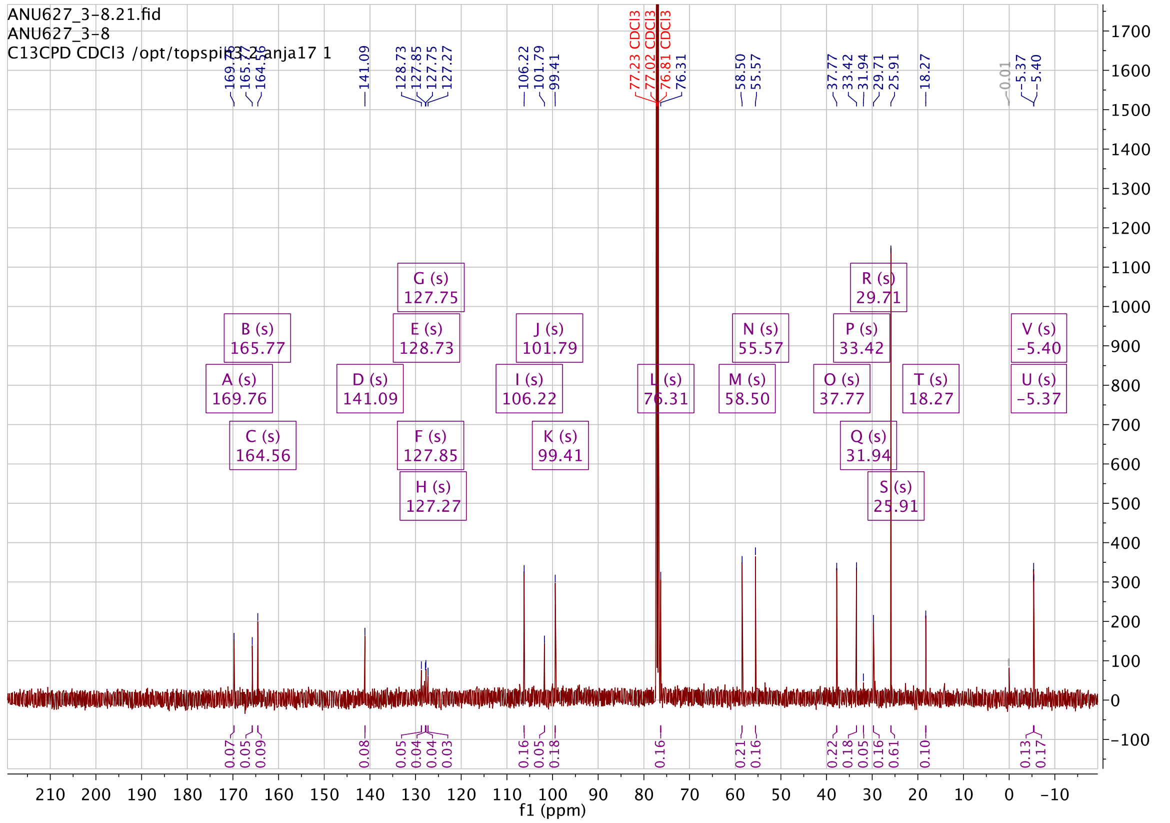

Methyl (*E*)-4-((2*R*)-2-(2-((*tert*-butyldimethylsilyl)oxy)ethyl)-6-oxotetrahydro-2*H*-pyran-4-yl)-3-methoxybut-2-enoate

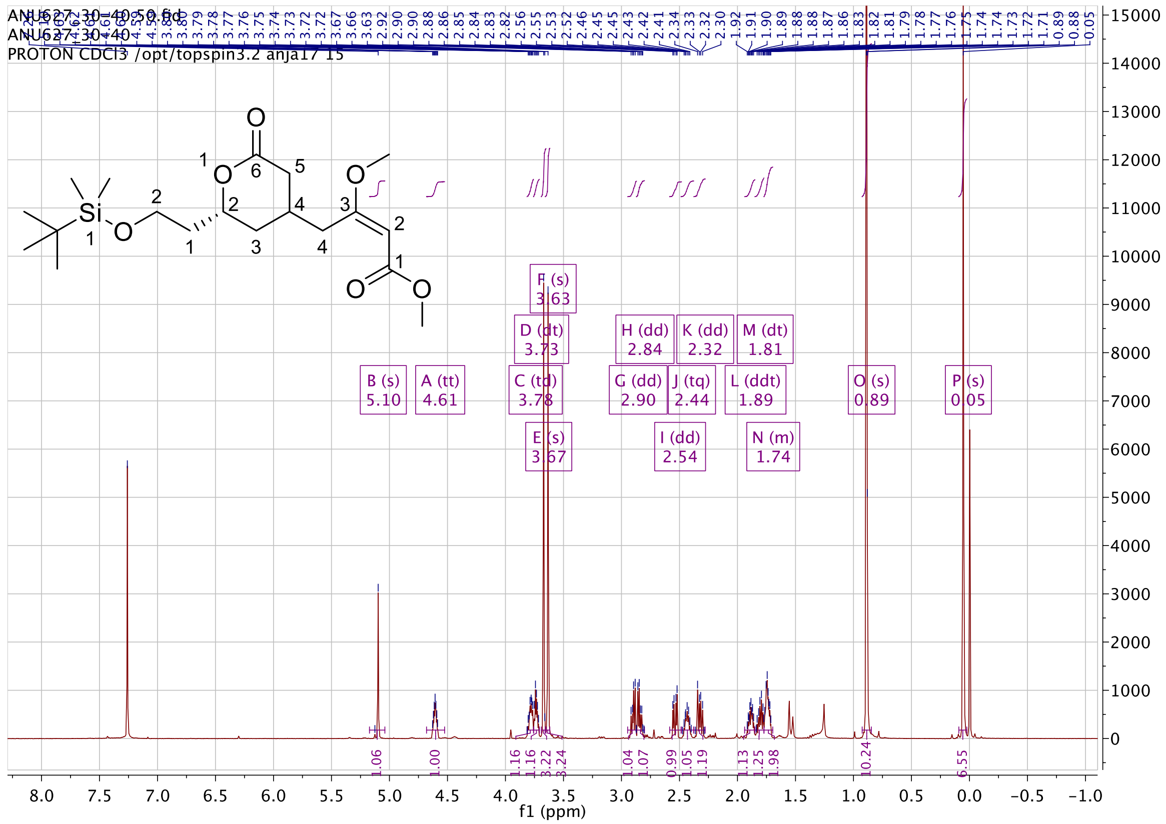

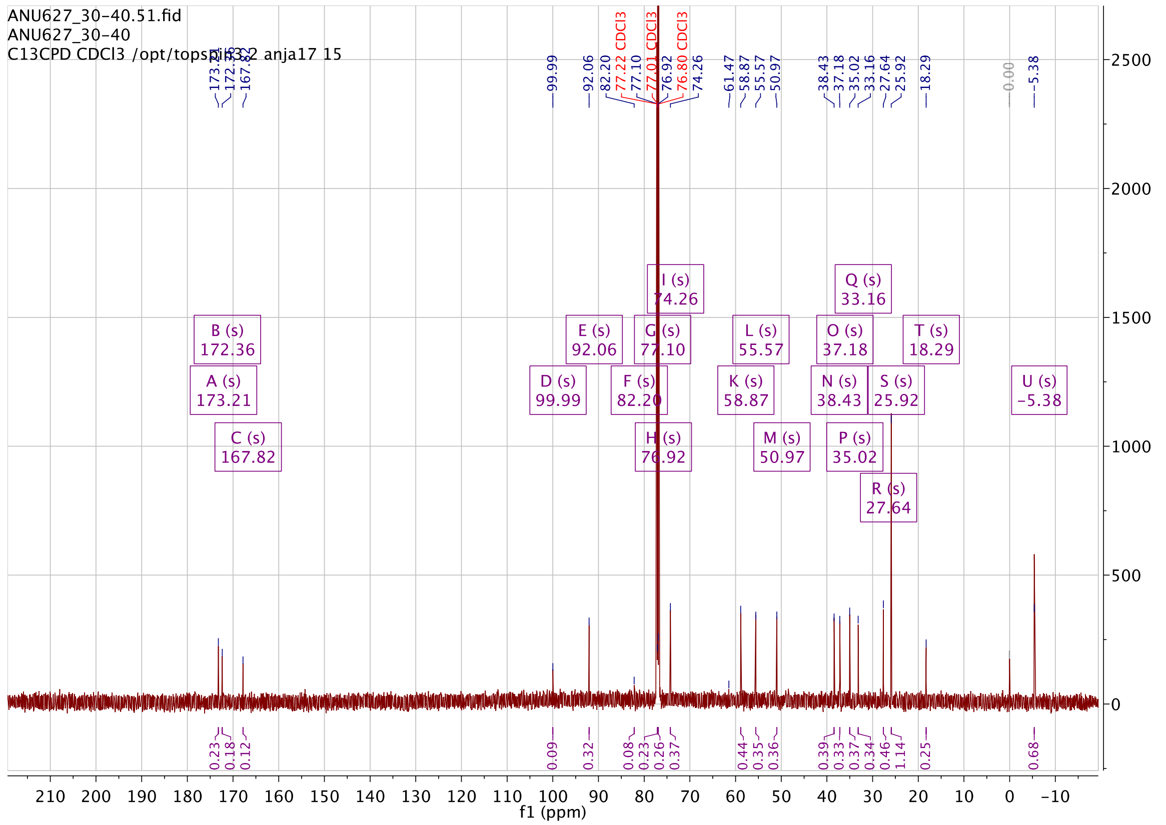

7-Hydroxy-9-methoxy-6*H*-benzo[*c*]chromen-6-one (21)

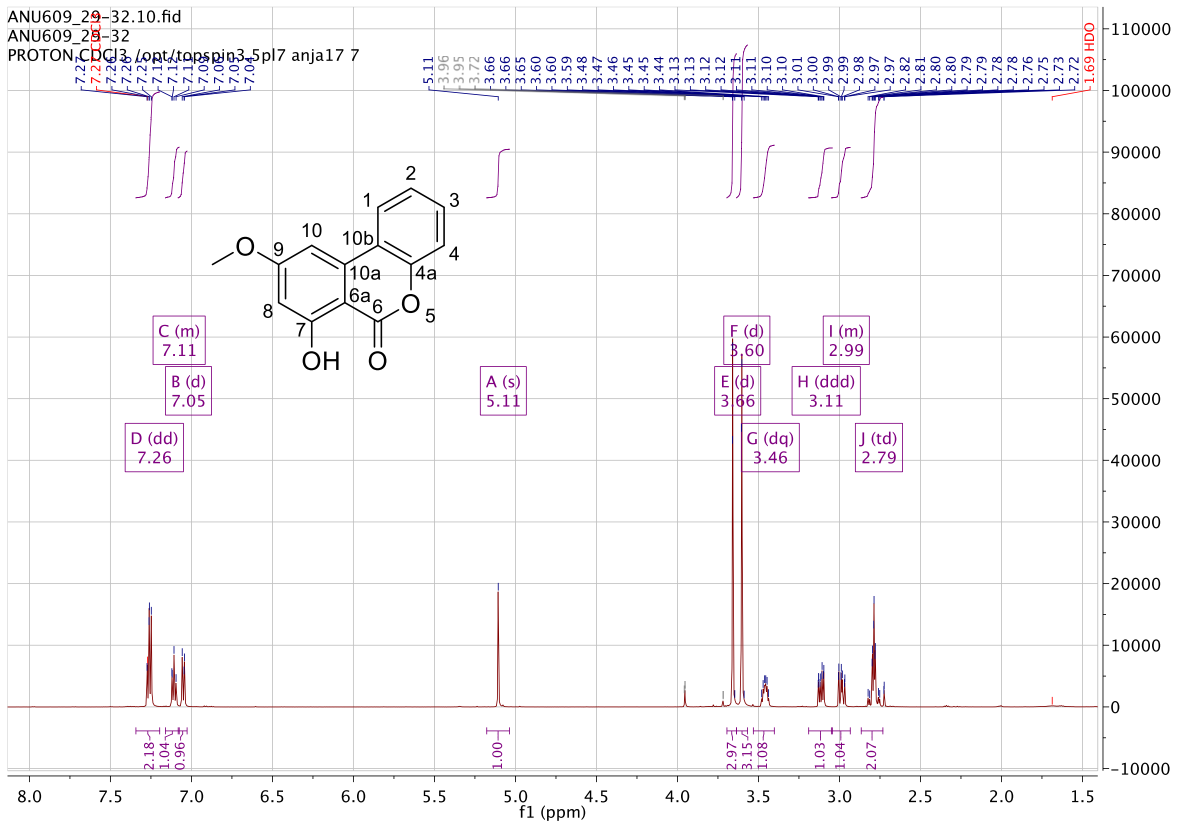

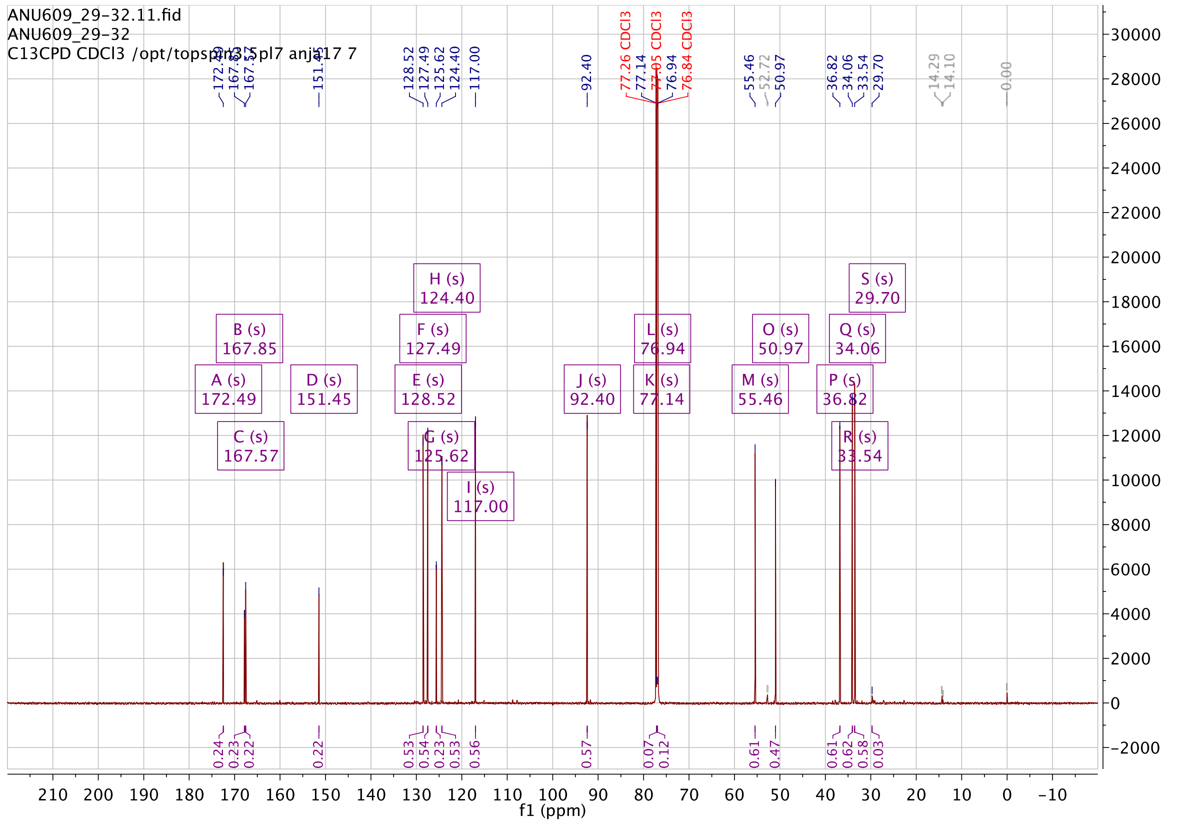

Methyl (*E*)-3-methoxy-4-(2-oxochroman-4-yl)but-2-enoate

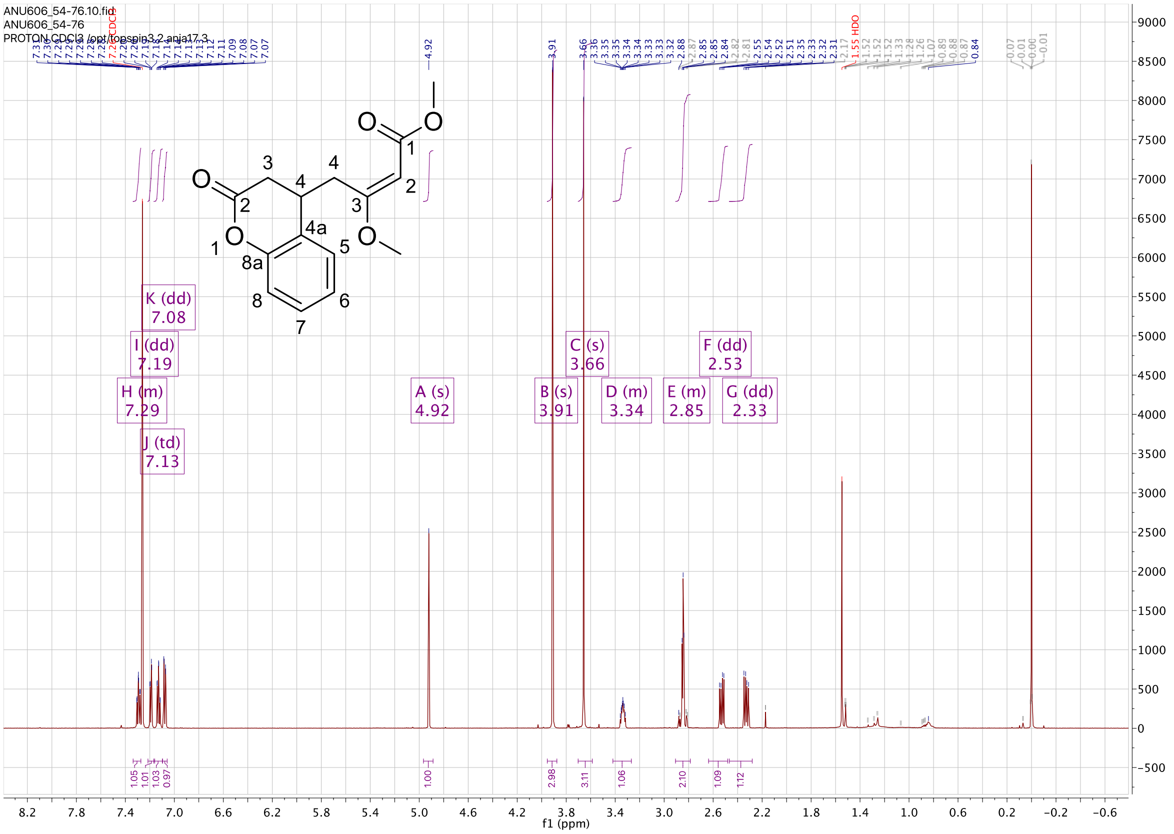

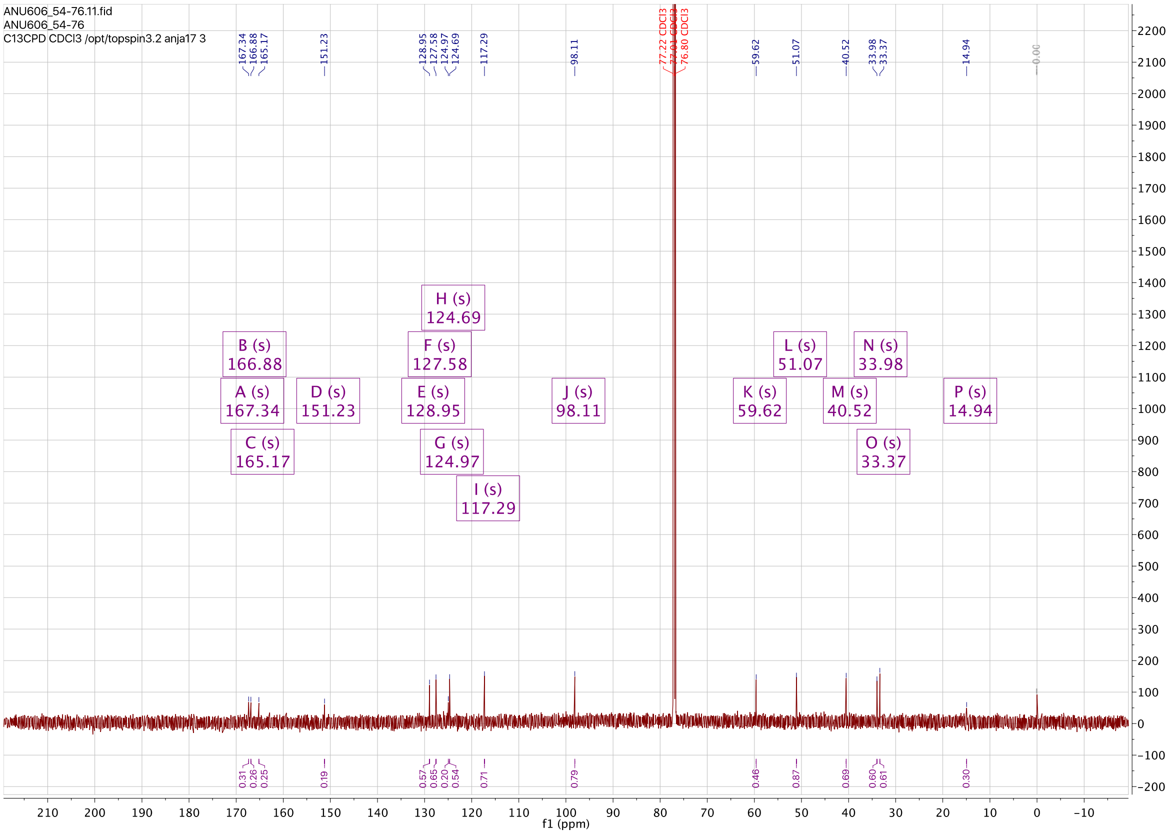

7-Hydroxy-3,9-dimethoxy-6H-benzo[c]chromen-6-one (23)

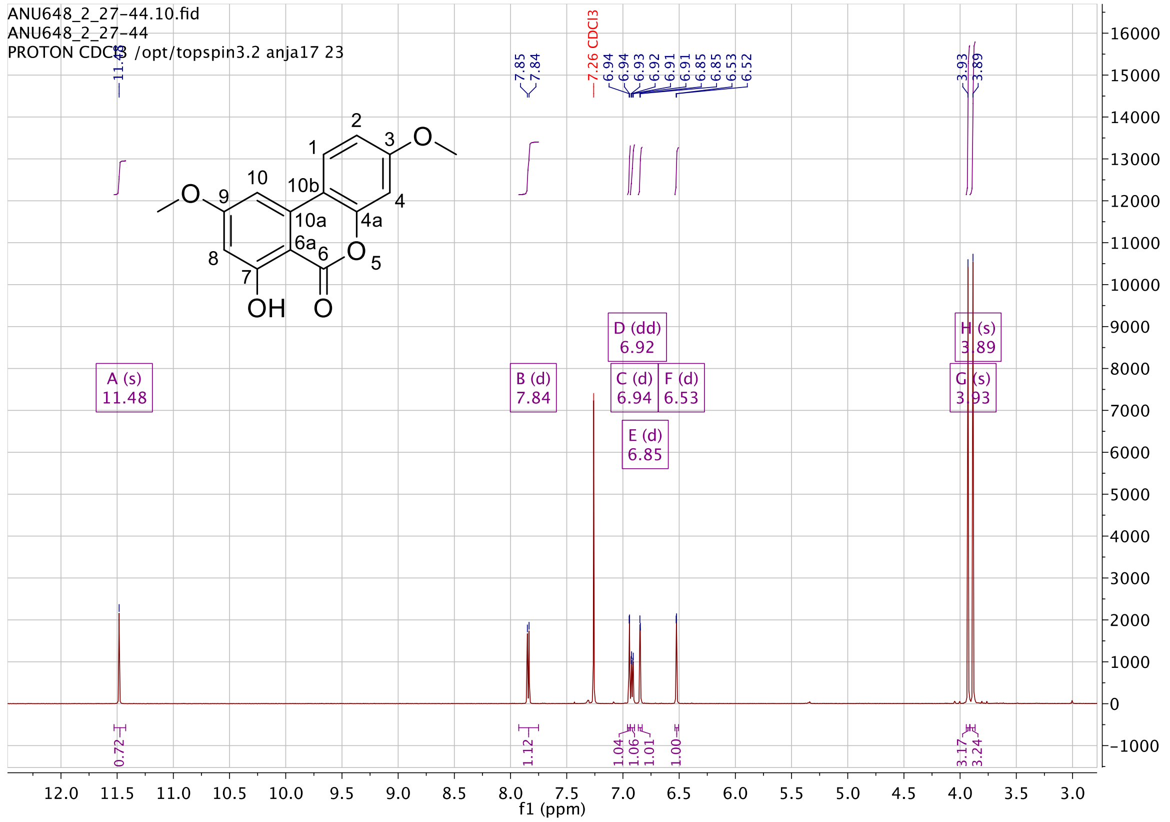

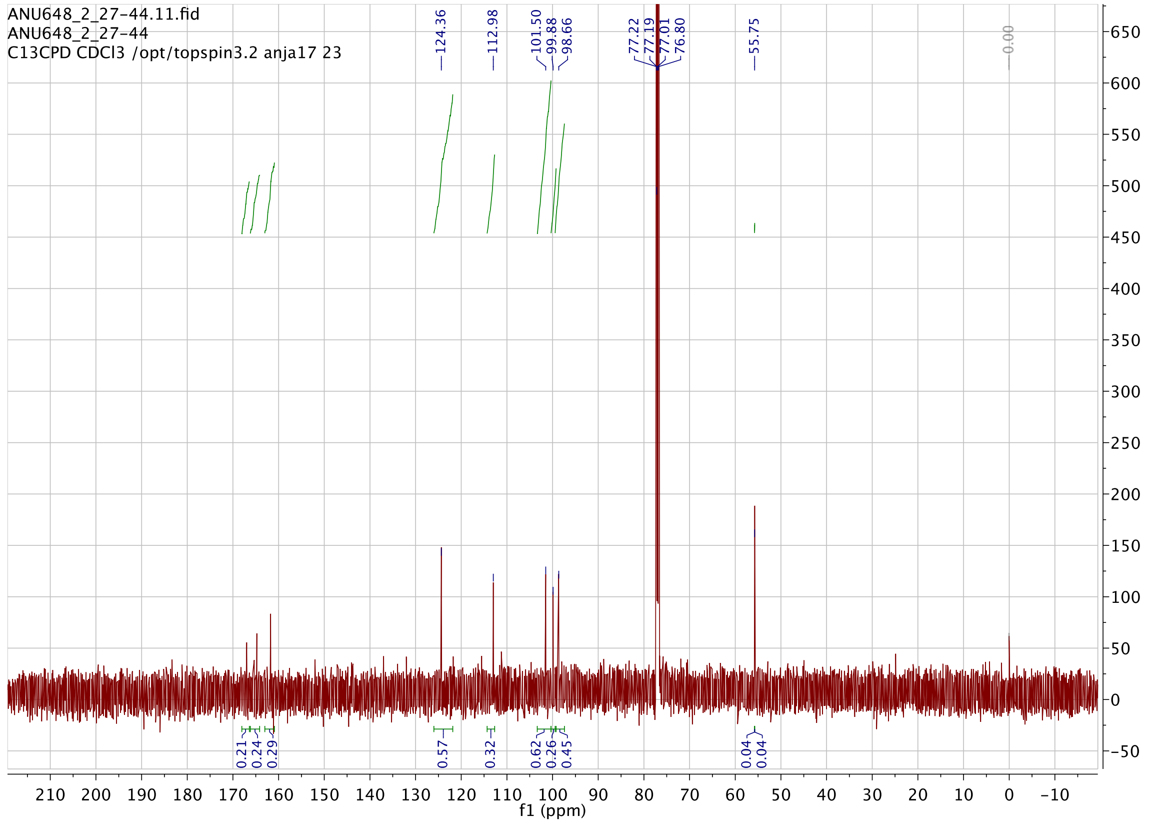

Methyl (*E*)-3-methoxy-4-(7-methoxy-2-oxochroman-4-yl)but-2-enoate

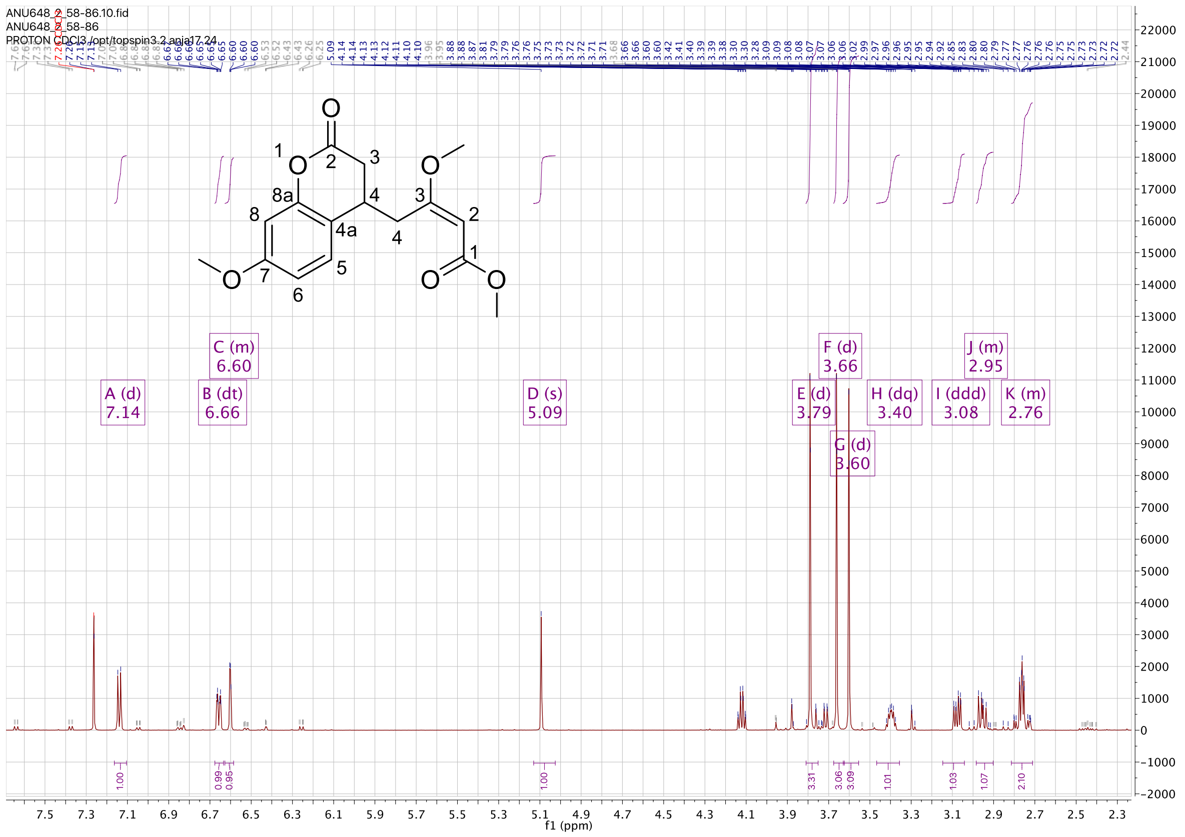

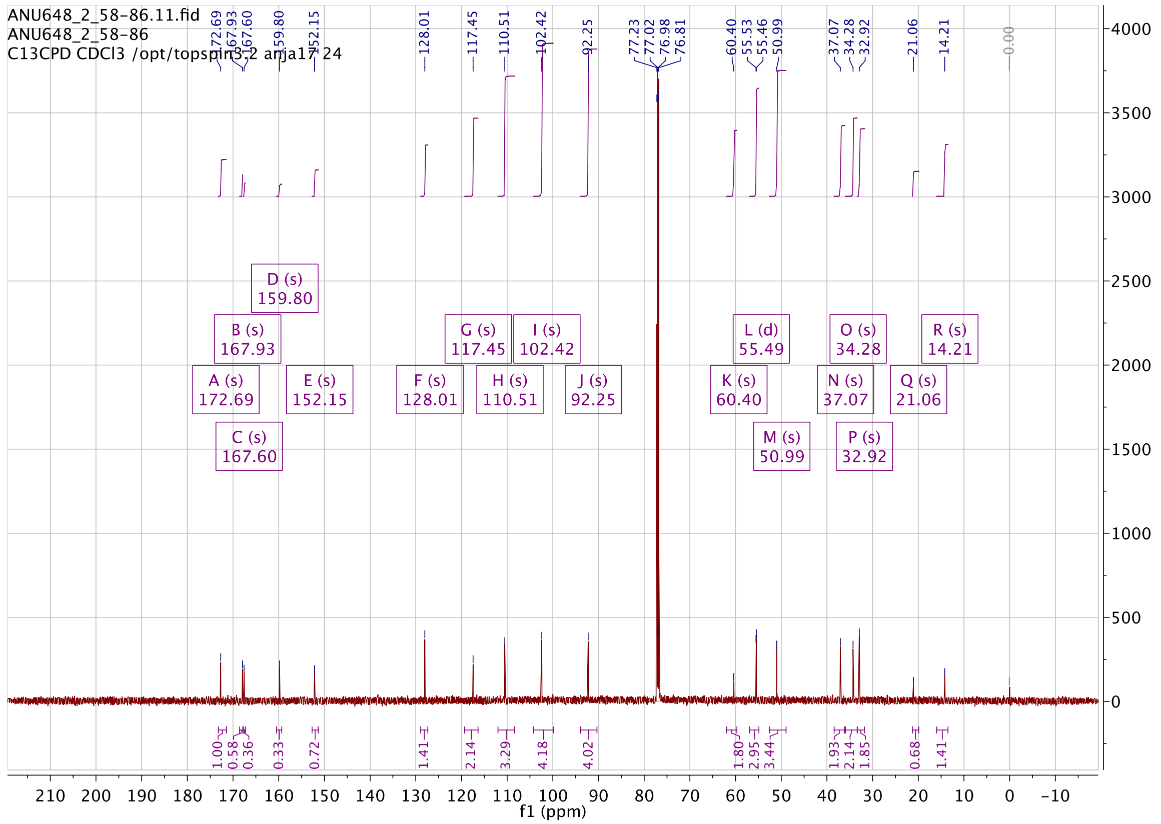

7-Hydroxy-3,9-dimethoxy-1-methyl-6*H*-benzo[*c*]chromen-6-one (24)

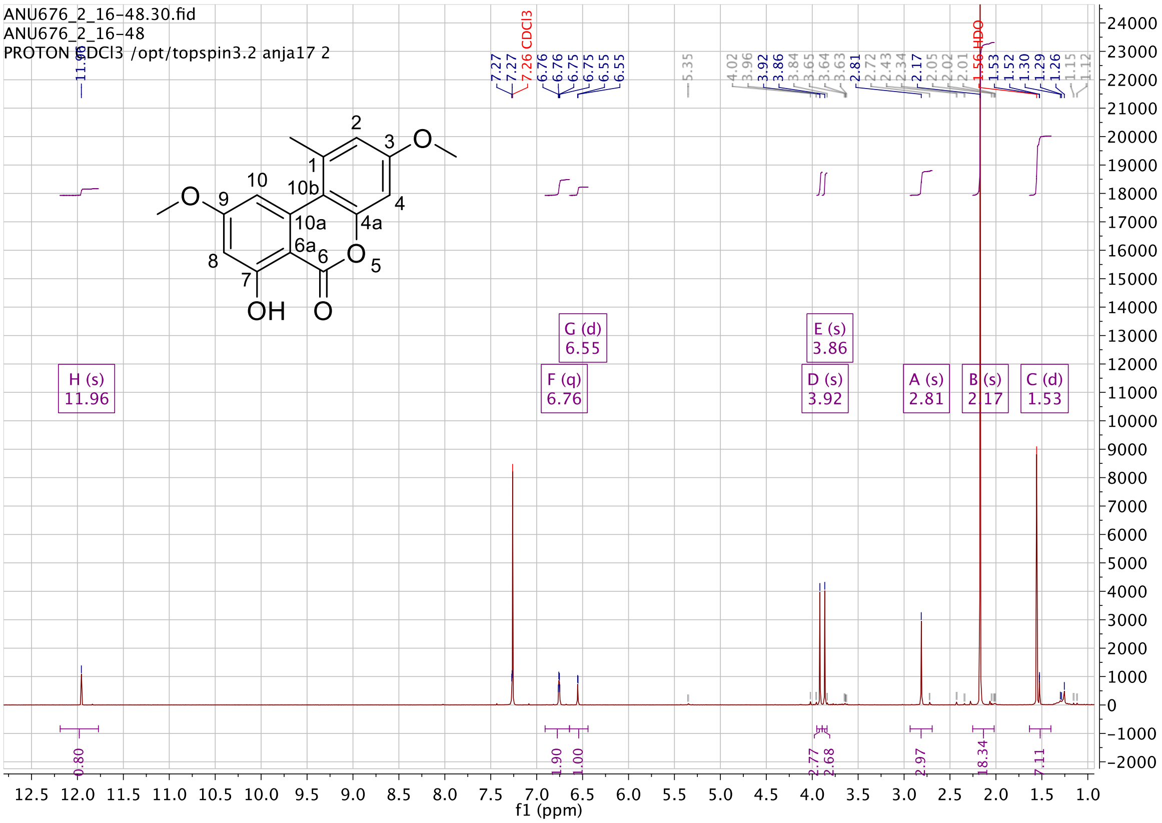

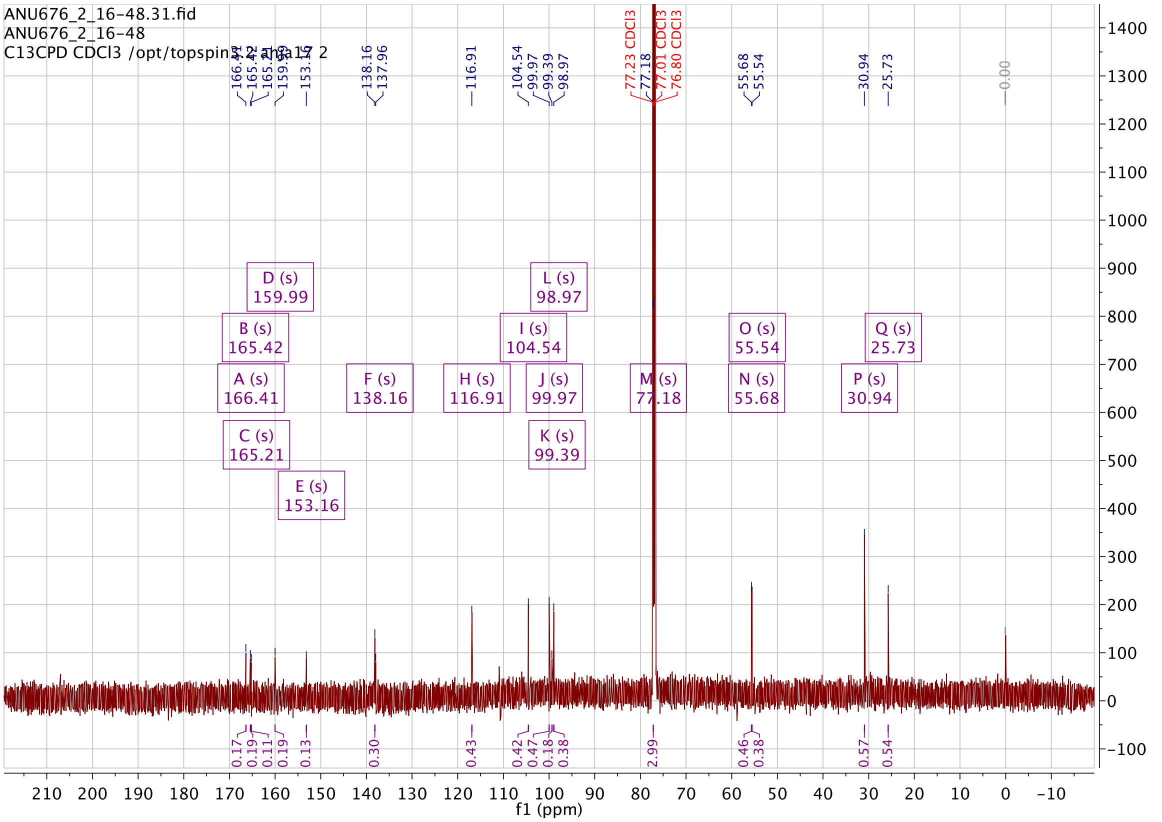

Methyl (*E*)-3-methoxy-4-(7-methoxy-5-methyl-2-oxochroman-4-yl)but-2-enoate

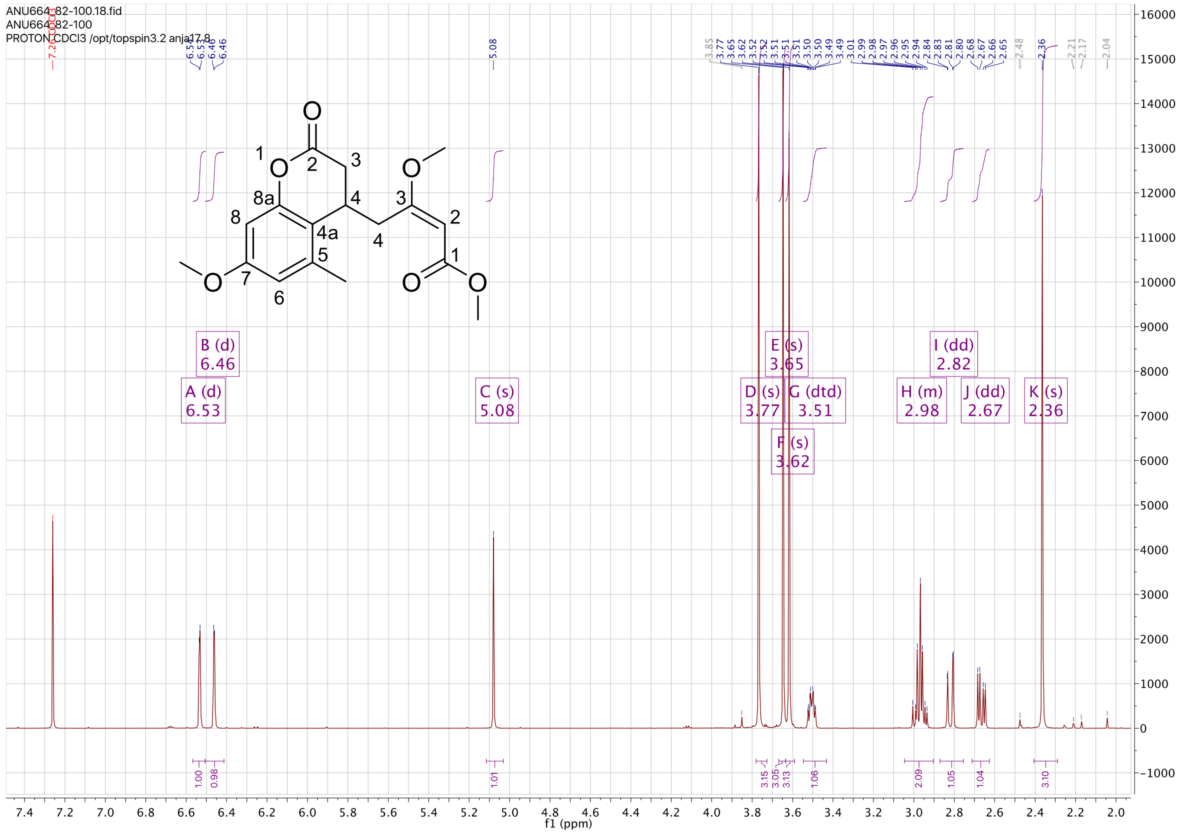

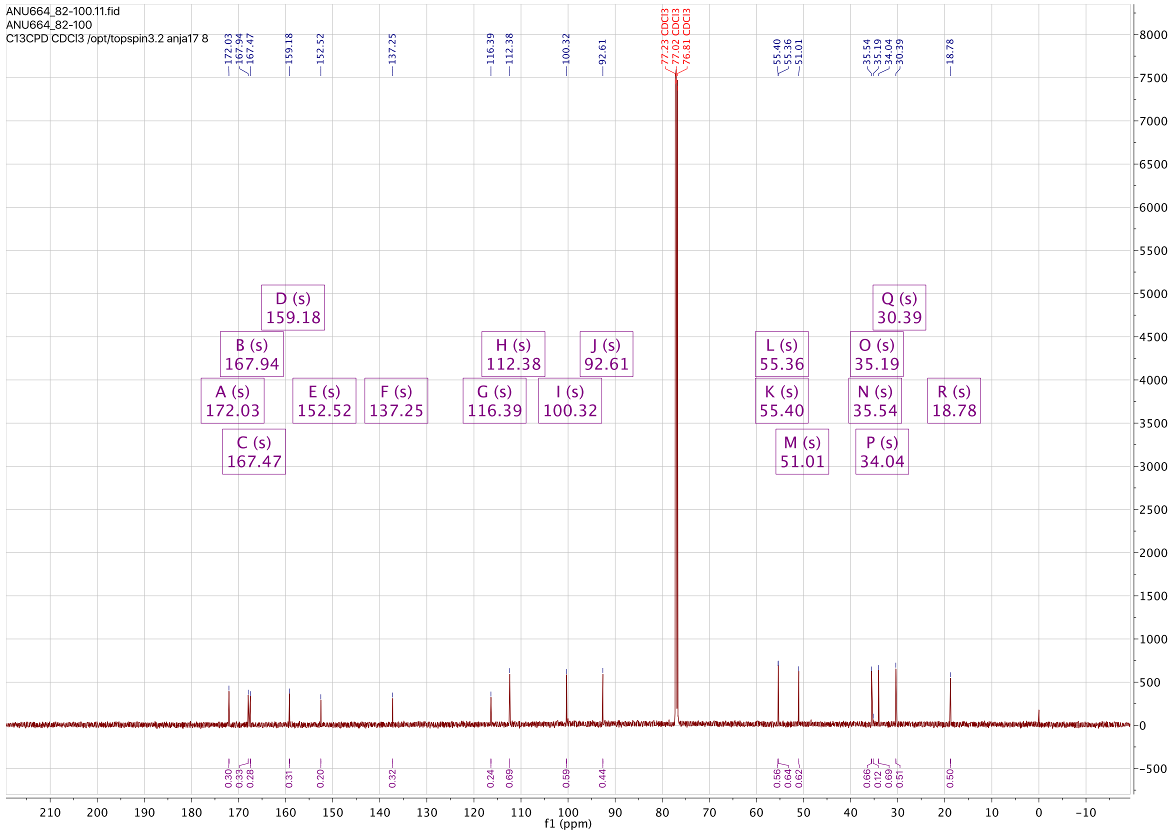

7-Hydroxy-1,9-dimethoxy-3-methyl-6*H*-benzo[*c*]chromen-6-one (27)

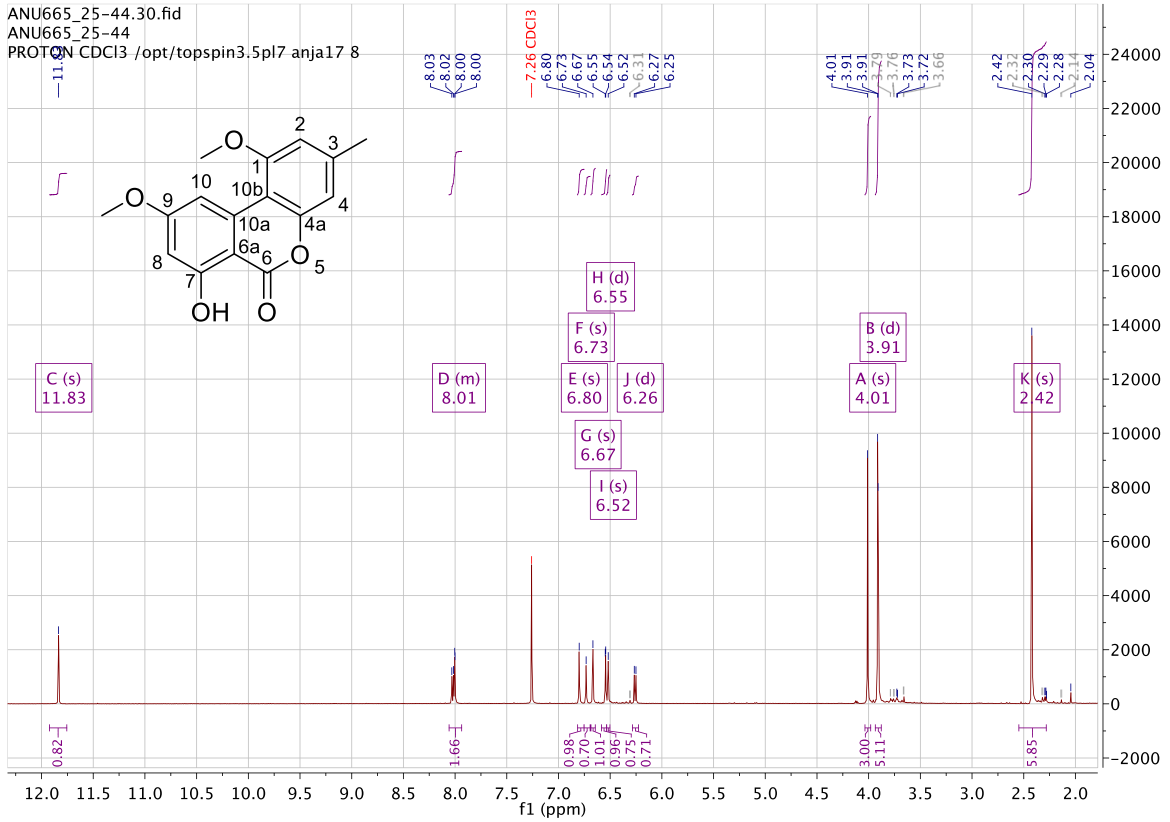

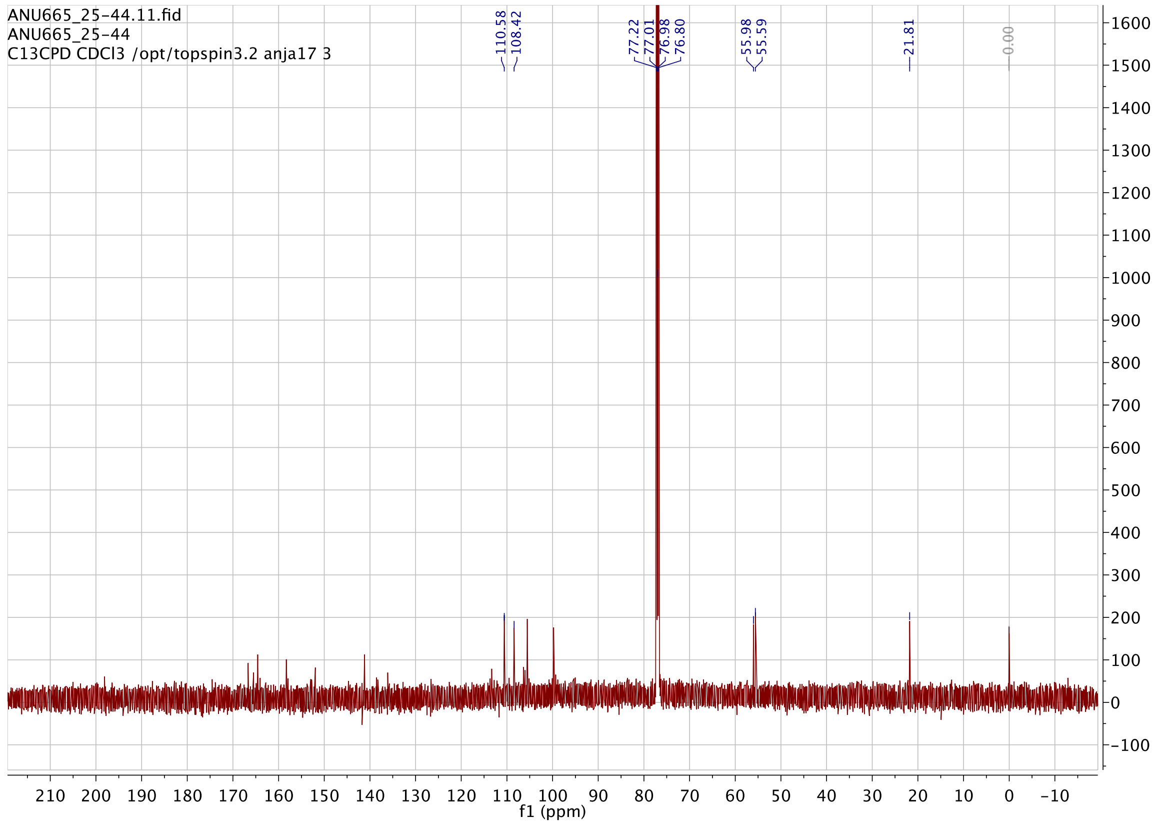

Methyl (*E*)-3-methoxy-4-(5-methoxy-7-methyl-2-oxochroman-4-yl)but-2-enoate

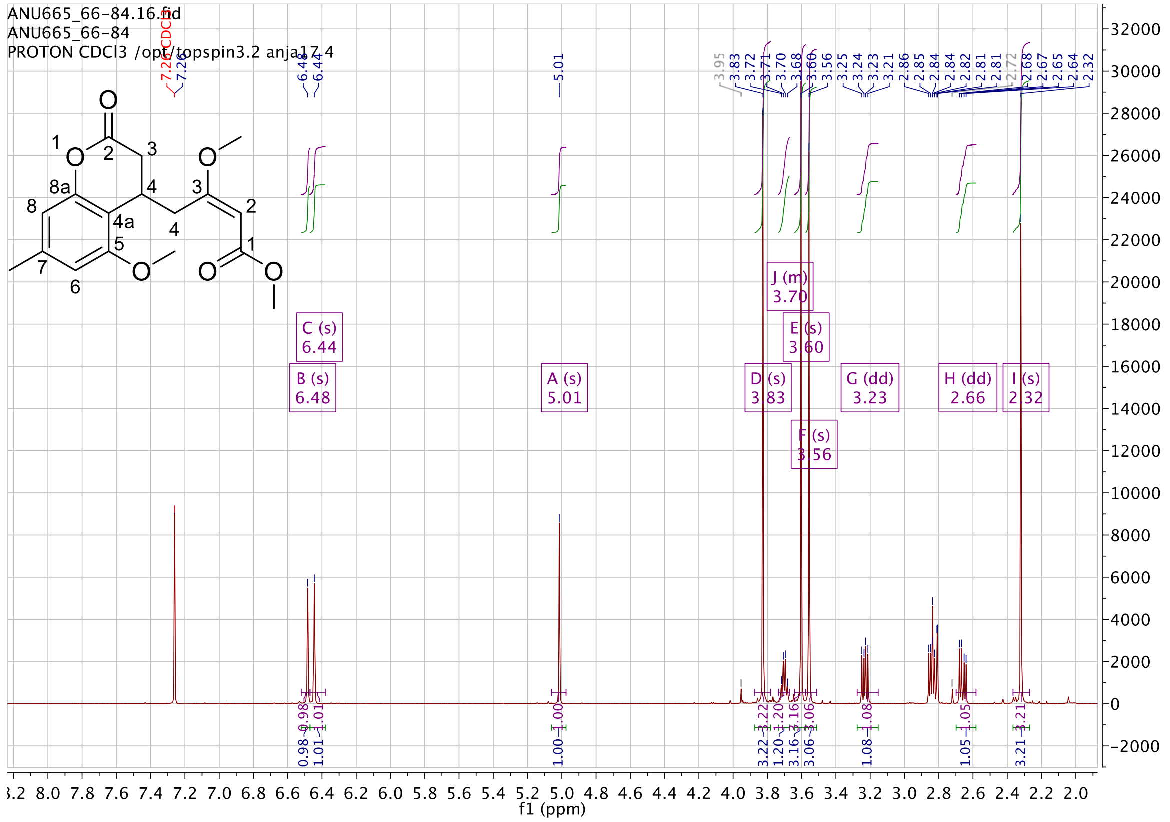

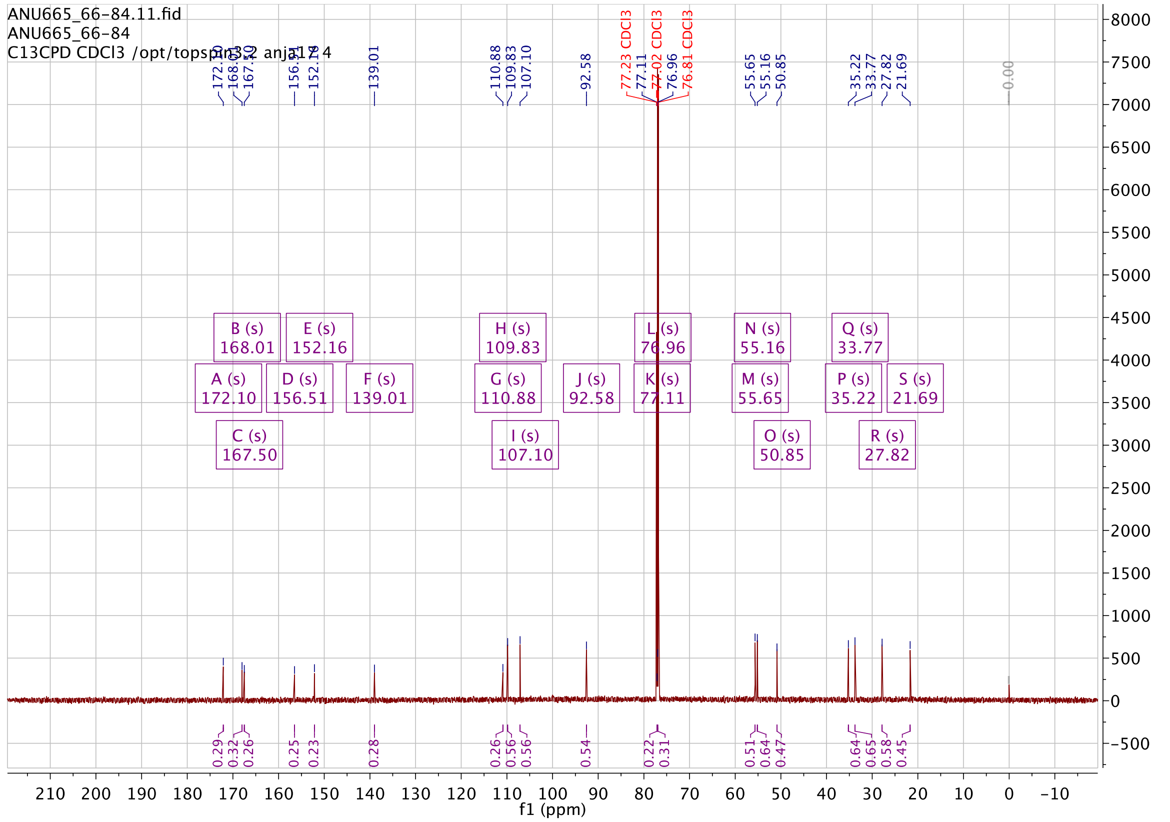

6. Computational Details

For the computational investigations, the conformational space for each structure was explored using the OPLS-2005 force field[4] and a modified Monte Carlo search algorithm implemented in MacroModel.[5] An energy cut-off of 20 kcal mol^{-1} was employed for the conformational analysis, and structures with heavy-atom root-mean-square deviations (RMSD) up to 1.0 Å after the initial force field optimizations were considered to be the same conformer. The remaining structures were subsequently optimized with the meta-GGA M06-L functional[6] with Grimme's dispersion correction D3 (zero-damping),[7] the double-ζ basis set 6-31+G(d,p) and the integral equation formalism polarizable continuum model (IEFPCM)[8] for toluene. Vibrational analysis verified that each structure was a minimum or transition state. Thermal corrections were obtained from unscaled harmonic vibrational frequencies at the same level of theory for a standard state of 1 mol L^{-1} and 298.15 K. Entropic contributions to the reported free energies were derived from partition functions evaluated with the quasiharmonic approximation by Cramer, Truhlar, and coworkers.[9] Electronic energies were subsequently obtained from single point calculations of the M06-L-D3 geometries employing the meta-hybrid M06-2X functional,[10] Grimme's dispersion-correction D3 (zero-damping),[7] the large quadruple-ζ basis set def2-QZVP,[11] and the integral equation formalism polarizable continuum model (IEFPCM) for toluene,[8] a level expected to give accurate energies. An ultrafine grid was used throughout this study for numerical integration of the density. All density functional theory calculations were performed with Gaussian 09.[12]

7. Functional Test for the $AlMe_3$-Catalysed Reaction

Table S4: Influence of the Functional used for the Single-Point Calculations for the $AlMe_3$-Catalysed Reaction

Method	**7–$AlMe_3$**	**TS3a**	**(*E*)-13–$AlMe_3$**	**TS3b**	***exo*-I1-$AlMe_3$**	***exo*-I1**
M06-2X-D3/def2-QZVP/IEFPCM//<br>M06-L-D3/6-31+G(d,p)/IEFPCM	–11.3	+3.5	–8.4	–6.9	–30.3	–18.3
ωB97X-D/def2-QZVP/IEFPCM//<br>M06-L-D3/6-31+G(d,p)/IEFPCM	–8.7	+6.8	–5.1	–4.1	–27.4	–18.5
B3LYP-D3/def2-QZVP/IEFPCM//<br>M06-L-D3/6-31+G(d,p)/IEFPCM	–8.7	+4.3	–1.4	–0.9	–17.3	–8.5
DSD-BLYP-D3BJ/def2-TZVPPD/IEFPCM//<br>M06-L-D3/6-31+G(d,p)/IEFPCM	–8.6	+6.8	–3.0	–3.2	–25.4	–16.8

8. Interaction of $AlMe_3$ with the Reactants

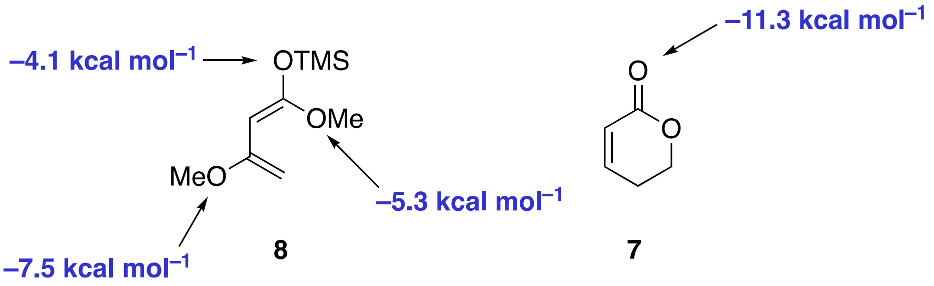

Scheme S1: Calculated interaction free energies of $AlMe_3$ with the reactants **7** and **8**.

9. Calculated Energies and Geometries for the Uncatalysed Reaction

9.1 Lactone 7

SCF energy:	–344.604729 hartree
Zero-point correction:	+0.103783 hartree
Enthalpy correction:	+0.110495 hartree
Free energy correction:	+0.074218 hartree
Truhlar's Delta G correction:	+0.074218 hartree
Grimme's Delta G correction:	+0.074248 hartree

Cartesian Coordinates

C	-0.32348	1.24313	0.19515
C	1.00338	1.27661	0.00226
C	1.75242	0.01747	-0.27633
C	0.98640	-1.15395	0.29458
O	-0.39848	-1.15565	-0.11329
H	1.01538	-1.12843	1.39154
H	1.37822	-2.11596	-0.03803
C	-1.09528	0.00562	0.00348
O	-2.30445	-0.01926	-0.12292
H	2.75244	0.04847	0.16842
H	1.90574	-0.09544	-1.35880
H	-0.90946	2.13519	0.38607
H	1.54052	2.22213	0.02556

9.2 Brassard's diene (8)

SCF energy:	–869.019931 hartree
Zero-point correction:	+0.256884 hartree
Enthalpy correction:	+0.276264 hartree
Free energy correction:	+0.208768 hartree
Truhlar's Delta G correction:	+0.212971 hartree
Grimme's Delta G correction:	+0.212369 hartree

Cartesian Coordinates

C	2.42385	-0.04358	-0.00026
C	0.97810	-0.13068	0.01390
C	0.07277	0.88128	0.01703
H	0.58421	-1.13962	0.02611
C	3.20996	1.05643	-0.02281
H	2.75927	2.03706	-0.03245
H	4.28963	0.99200	-0.03192
O	0.47234	2.16801	0.01014
O	-1.25403	0.73111	0.02908
O	2.92717	-1.32840	0.01224
C	-0.53388	3.18040	0.00774
H	-1.16637	3.10900	-0.88065
H	0.01244	4.12254	0.00079
H	-1.16090	3.11887	0.90066
Si	-2.18798	-0.70752	-0.00515
C	-1.82862	-1.62970	-1.58549

H	-2.51140	-2.47884	-1.69237
H	-0.80883	-2.02144	-1.62282
H	-1.96783	-0.98362	-2.45724
C	-3.92123	-0.03012	0.02318
H	-4.10801	0.61752	-0.83816
H	-4.10288	0.55708	0.92789
H	-4.66029	-0.83658	-0.00208
C	-1.82001	-1.71971	1.51762
H	-0.82308	-2.16742	1.50164
H	-2.54461	-2.53521	1.61122
H	-1.89459	-1.10856	2.42192
C	4.33212	-1.46389	0.00116
H	4.53545	-2.53491	0.01357
H	4.78678	-0.99356	0.88263
H	4.77051	-1.01727	-0.90066

9.3 Transition State TS1*endo*

SCF energy:	–1213.607733 hartree
Zero-point correction:	+0.363424 hartree
Enthalpy correction:	+0.388054 hartree
Free energy correction:	+0.311997 hartree
Truhlar's Delta G correction:	+0.314321 hartree
Grimme's Delta G correction:	+0.314630 hartree
Imaginary Frequency:	437.0 *i*cm^{-1}

Cartesian Coordinates

C	1.73705	1.30224	0.90096
C	0.34413	1.19748	0.70303
C	-0.50842	0.28281	1.30434
C	1.34239	-1.90654	0.27272
C	2.60387	-1.31051	0.35036
C	2.54363	0.37180	1.56147
H	3.59499	0.63602	1.64651
H	2.11982	-0.14815	2.40839
H	-0.12007	1.79809	-0.06753
O	2.44614	2.24837	0.21440
C	1.75448	3.19054	-0.59046
H	1.00127	3.73512	-0.00989
H	1.27205	2.71270	-1.45356
H	2.51221	3.88636	-0.94836
O	-1.72670	0.03710	0.90082
O	-0.16547	-0.32675	2.43933
C	0.51966	-1.88699	-0.88745
H	0.92734	-2.43091	1.12688
O	1.03354	-1.31558	-2.04561
C	2.11759	-0.38866	-1.90342
C	3.20061	-0.86052	-0.95591
H	1.70090	0.56904	-1.56212
H	2.50502	-0.23901	-2.91468
H	3.92899	-0.05376	-0.81975
H	3.74343	-1.70583	-1.40143
H	3.30993	-1.73227	1.06399
O	-0.60265	-2.39183	-0.99016
C	-0.99126	-1.42272	2.86254

H	-1.96417	-1.06289	3.20286
H	-1.12946	-2.13394	2.04353
H	-0.44993	-1.88429	3.68668
C	-1.62683	0.74882	-2.00662
H	-0.84816	-0.00834	-2.13666
H	-2.27645	0.69566	-2.88754
H	-1.16743	1.74212	-2.00773
C	-3.73164	-1.08813	-0.67118
H	-4.28205	-1.29226	0.25175
H	-4.45935	-0.98091	-1.48094
H	-3.09349	-1.94848	-0.89169
C	-3.61361	1.94997	0.01081
H	-4.21942	1.77064	0.90339
H	-2.93195	2.77813	0.23099
H	-4.28592	2.28365	-0.78587
Si	-2.67263	0.42325	-0.50390

9.4 Transition State TS1*exo*

SCF energy:	–1213.608262 hartree
Zero-point correction:	+0.363510 hartree
Enthalpy correction:	+0.388163 hartree
Free energy correction:	+0.312025 hartree
Truhlar's Delta G correction:	+0.313968 hartree
Grimme's Delta G correction:	+0.314488 hartree
Imaginary Frequency:	393.0 $i$cm^{-1}

Cartesian Coordinates

C	-1.30657	1.88342	0.47491
C	0.03740	1.48157	0.56527
C	0.52717	0.27140	1.03162
C	-1.20649	-0.61535	-1.34827
C	-2.47226	-0.14893	-0.94996
C	-2.41697	1.04096	0.65324
H	-2.29948	0.27753	1.41091
H	-3.40464	1.49392	0.64633
H	0.76964	2.15200	0.12819
O	-1.41591	3.11426	-0.09805
C	-2.71386	3.66492	-0.27245
H	-3.24724	3.73322	0.68216
H	-3.30887	3.07704	-0.97934
H	-2.55837	4.66405	-0.67718
O	-0.22201	-0.53576	1.78289
C	0.35157	-1.80280	2.16624
H	0.68509	-2.35404	1.28546
H	-0.45862	-2.33532	2.66192
H	1.18226	-1.64649	2.85715
O	1.76807	-0.11871	0.88191
Si	2.97867	0.32571	-0.28265
C	3.74092	1.91306	0.33259
H	3.03393	2.74785	0.31554
H	4.59192	2.19691	-0.29478
H	4.10797	1.80795	1.35759
C	4.13240	-1.12357	-0.16084
H	3.61212	-2.04192	-0.44850

H	4.50912	-1.25187	0.85780
H	4.99448	-1.00494	-0.82374
C	2.20498	0.48188	-1.96164
H	2.98887	0.61207	-2.71521
H	1.52161	1.33173	-2.04290
H	1.65230	-0.43525	-2.19286
C	-0.68523	-1.88635	-0.98703
H	-0.54446	0.01198	-1.93378
O	-1.48856	-2.75015	-0.25250
C	-2.67112	-2.23217	0.37090
C	-3.41749	-1.22727	-0.47802
H	-2.38324	-1.77701	1.33010
H	-3.28768	-3.10711	0.59435
H	-4.25355	-0.82249	0.10394
H	-3.85179	-1.72376	-1.35567
H	-2.92303	0.60840	-1.59288
O	0.42637	-2.33198	-1.29387

9.5 Cycloadduct *endo*-I1

SCF energy:	–1213.684561 hartree
Zero-point correction:	+0.367883 hartree
Enthalpy correction:	+0.391916 hartree
Free energy correction:	+0.316634 hartree
Truhlar's Delta G correction:	+0.319422 hartree
Grimme's Delta G correction:	+0.319495 hartree

Cartesian Coordinates

C	-2.64681	-0.32203	-0.17641
C	-1.49860	-0.96420	0.09567
C	-0.23615	-0.26462	0.50216
C	-0.48274	1.22790	0.87094
C	-1.48754	1.90683	-0.06340
C	-2.81579	1.15853	-0.07011
H	-3.44671	1.50125	-0.89854
H	-3.38079	1.38098	0.84582
H	-1.40126	-2.04087	-0.00813
O	-3.80701	-0.91546	-0.56203
C	-3.80159	-2.33271	-0.64610
H	-3.55386	-2.78199	0.32310
H	-3.08060	-2.67958	-1.39560
H	-4.80838	-2.62369	-0.94207
O	0.69942	-0.32215	-0.53947
O	0.38870	-0.96604	1.58231
C	0.85266	1.93189	0.90559
H	-0.87510	1.25171	1.89366
O	1.27133	2.58322	-0.19977
C	0.41097	2.88346	-1.32832
C	-0.85083	2.05523	-1.43867
H	1.05256	2.77253	-2.20583
H	0.16238	3.94720	-1.22636
H	-0.63735	1.07173	-1.86690
H	-1.53123	2.57412	-2.12353
H	-1.67537	2.91690	0.32914
O	1.58786	1.89870	1.87109

C	-0.32909	-1.04348	2.79695
H	0.12764	-1.84650	3.37826
H	-0.25450	-0.11172	3.37320
H	-1.38805	-1.28367	2.62774
C	1.71315	-3.07685	-0.47983
H	1.31955	-3.32286	0.51053
H	0.96749	-3.37359	-1.22470
H	2.60582	-3.68903	-0.64728
C	2.71808	-0.81369	-2.32313
H	2.90691	0.26309	-2.39069
H	1.97859	-1.07054	-3.08779
H	3.65139	-1.32832	-2.57223
C	3.38186	-0.73593	0.66027
H	4.34311	-1.22317	0.46198
H	3.54281	0.34611	0.63352
H	3.06591	-0.99264	1.67406
Si	2.11579	-1.25327	-0.60801

9.6 Cycloadduct *exo*-I1

SCF energy:	–1213.682863 hartree
Zero-point correction:	+0.367793 hartree
Enthalpy correction:	+0.391979 hartree
Free energy correction:	+0.314998 hartree
Truhlar's Delta G correction:	+0.319219 hartree
Grimme's Delta G correction:	+0.318617 hartree

Cartesian Coordinates

C	-0.16001	2.16529	-0.40607
C	0.32513	1.25905	0.46251
C	-0.12354	-0.17461	0.49018
C	-0.99404	-0.53241	-0.73458
C	-1.96373	0.58161	-1.13281
C	-1.19708	1.86259	-1.43918
H	-1.87837	2.71801	-1.51578
H	-0.70182	1.78635	-2.41752
H	1.08630	1.52570	1.18849
O	0.24180	3.45971	-0.49217
C	1.27250	3.87300	0.39193
H	0.96038	3.76757	1.43779
H	1.46322	4.92143	0.16847
H	2.18593	3.28751	0.22961
O	-0.94768	-0.46843	1.61794
C	-0.38227	-0.14090	2.87399
H	-0.36316	0.94577	3.03771
H	0.63342	-0.54317	2.96972
H	-1.02028	-0.60082	3.62971
O	0.97727	-1.04696	0.58004
Si	2.46615	-0.98338	-0.20763
C	2.28433	-0.49158	-2.00668
H	1.80928	0.49009	-2.11463
H	1.70011	-1.22025	-2.57742
H	3.27156	-0.42547	-2.47679
C	3.60530	0.22983	0.65909
H	3.36868	1.26873	0.40725

H	3.53925	0.12616	1.74740
H	4.64892	0.05600	0.37567
C	3.10861	-2.72345	-0.04037
H	4.09152	-2.83681	-0.50850
H	2.42395	-3.43387	-0.51255
H	3.20487	-3.00903	1.01116
C	-1.73349	-1.83173	-0.52026
H	-0.29968	-0.71570	-1.56027
O	-3.01486	-1.75582	-0.10657
C	-3.61353	-0.54871	0.42899
C	-3.01166	0.77574	-0.04317
H	-3.55845	-0.63838	1.51711
H	-4.66073	-0.64160	0.13199
H	-2.54808	1.28851	0.80624
H	-3.80804	1.42797	-0.41683
H	-2.47207	0.25758	-2.05047
O	-1.26928	-2.92066	-0.78702

10. Calculated Energies and Geometries for the Brønsted-Acid Catalysed Reaction

10.1 Bis(trifluoromethanesulfonyl)methane Tf_2CH_2

SCF energy:	–1811.962753 hartree
Zero-point correction:	+0.076760 hartree
Enthalpy correction:	+0.092740 hartree
Free energy correction:	+0.032961 hartree
Truhlar's Delta G correction:	+0.035493 hartree
Grimme's Delta G correction:	+0.035307 hartree

Cartesian Coordinates

C	0.00005	0.00033	1.28739
S	1.27225	-0.90167	0.36249
C	2.36740	0.46386	-0.37531
F	3.60904	-0.00560	-0.40767
F	2.31847	1.54262	0.41296
F	1.97596	0.77567	-1.59751
S	-1.27237	0.90182	0.36234
H	0.47622	0.75052	1.92360
H	-0.47594	-0.74941	1.92423
C	-2.36721	-0.46399	-0.37530
F	-3.60897	0.00520	-0.40769
O	-0.64713	1.60719	-0.74477
O	-2.09384	1.55705	1.36908
O	0.64682	-1.60692	-0.74460
O	2.09355	-1.55703	1.36928
F	-2.31808	-1.54264	0.41317
F	-1.97585	-0.77602	-1.59744

10.2 Bis(trifluoromethanesulfonyl)methane Anion

SCF energy:	–1811.508182 hartree
Zero-point correction:	+0.064015 hartree
Enthalpy correction:	+0.079623 hartree
Free energy correction:	+0.021380 hartree
Truhlar's Delta G correction:	+0.023191 hartree
Grimme's Delta G correction:	+0.023207 hartree

Cartesian Coordinates

C	2.63184	-0.39024	-0.11399
S	1.24092	0.83835	0.15522
F	3.01254	-0.94207	1.05259
F	3.70142	0.23228	-0.64276
F	2.27344	-1.37788	-0.94369
O	1.85568	1.76329	1.11724
O	0.92416	1.26283	-1.20984
C	-0.00006	-0.00008	0.91609
S	-1.24101	-0.83848	0.15522
O	-0.92419	-1.26292	-1.20983
O	-1.85596	-1.76342	1.11712

C	-2.63176	0.39029	-0.11401
F	-3.01268	0.94188	1.05263
F	-3.70124	-0.23193	-0.64319
F	-2.27305	1.37814	-0.94337
H	-0.00009	0.00022	1.99714

10.3 Triflic Acid

SCF energy:	–962.181074 hartree
Zero-point correction:	+0.038627 hartree
Enthalpy correction:	+0.047377 hartree
Free energy correction:	+0.005648 hartree
Truhlar's Delta G correction:	+0.006185 hartree
Grimme's Delta G correction:	+0.006147 hartree

Cartesian Coordinates

C	-0.99847	0.00602	-0.00281
S	0.85926	-0.14162	0.07599
F	-1.36908	1.19410	0.47931
F	-1.53380	-0.96011	0.73830
F	-1.41391	-0.11047	-1.25951
O	1.21721	-1.33515	-0.64927
O	1.25135	1.11162	-0.86166
O	1.24317	0.12618	1.44667
H	1.39981	1.89698	-0.30784

10.4 Triflate Anion

SCF energy:	–961.740701 hartree
Zero-point correction:	+0.026995 hartree
Enthalpy correction:	+0.035102 hartree
Free energy correction:	–0.005287 hartree
Truhlar's Delta G correction:	–0.005015 hartree
Grimme's Delta G correction:	–0.004975 hartree

Cartesian Coordinates

C	0.94040	0.00010	0.00017
S	-0.92258	-0.00018	0.00010
F	1.43920	1.23425	0.22298
F	1.43829	-0.42357	-1.18054
F	1.43921	-0.81031	0.95716
O	-1.23805	-1.41815	-0.25253
O	-1.23921	0.49034	1.35390
O	-1.23916	0.92768	-1.10124

10.5 HCl

SCF energy:	–460.815849 hartree
Zero-point correction:	+0.006879 hartree
Enthalpy correction:	+0.010183 hartree
Free energy correction:	–0.011000 hartree

Truhlar's Delta G correction: −0.011000 hartree
Grimme's Delta G correction: −0.011000 hartree

Cartesian Coordinates

Cl	0.00000	0.00000	0.07111
H	0.00000	0.00000	-1.20887

10.6 Chloride Anion

SCF energy: −460.344493 hartree
Zero-point correction: +0.000000 hartree
Enthalpy correction: +0.002360 hartree
Free energy correction: −0.015023 hartree
Truhlar's Delta G correction: −0.015023 hartree
Grimme's Delta G correction: −0.015023 hartree

Cartesian Coordinates

Cl	0.00000	0.00000	0.00000

10.7 Oxonium Ion

SCF energy: −76.781190 hartree
Zero-point correction: +0.035108 hartree
Enthalpy correction: +0.038940 hartree
Free energy correction: +0.015982 hartree
Truhlar's Delta G correction: +0.015982 hartree
Grimme's Delta G correction: +0.015982 hartree

Cartesian Coordinates

O	-0.00002	0.00007	-0.08116
H	0.08297	-0.92717	0.21629
H	-0.84482	0.39155	0.21649
H	0.76199	0.53507	0.21646

10.8 Water

SCF energy: −76.439397 hartree
Zero-point correction: +0.021636 hartree
Enthalpy correction: +0.025416 hartree
Free energy correction: +0.003339 hartree
Truhlar's Delta G correction: +0.003339 hartree
Grimme's Delta G correction: +0.003339 hartree

Cartesian Coordinates

O	0.00000	0.11774	0.00000
H	0.76018	-0.47096	0.00000
H	-0.76018	-0.47096	0.00000

10.9 Protonated Lactone 7-H$^+$

SCF energy:	–344.988611 hartree
Zero-point correction:	+0.116909 hartree
Enthalpy correction:	+0.123790 hartree
Free energy correction:	+0.087299 hartree
Truhlar's Delta G correction:	+0.087299 hartree
Grimme's Delta G correction:	+0.087304 hartree

Cartesian Coordinates

C	1.07226	1.25847	0.00942
C	-0.26606	1.27246	0.19106
C	-0.97190	0.04295	0.01788
O	-0.40545	-1.11294	-0.07674
C	1.03564	-1.19729	0.26242
C	1.79652	-0.00802	-0.26517
H	1.34552	-2.14599	-0.16866
H	1.06570	-1.25960	1.35329
H	1.98200	-0.07688	-1.34644
H	2.78747	0.00466	0.19756
H	1.62515	2.19343	0.00983
H	-0.84689	2.17195	0.34792
O	-2.26018	0.07755	-0.10633
H	-2.63271	-0.81586	-0.22265

10.10 Transition State TS2a leading to *E*-Intermediate

SCF energy:	–1214.010023 hartree
Zero-point correction:	+0.375727 hartree
Enthalpy correction:	+0.401110 hartree
Free energy correction:	+0.322234 hartree
Truhlar's Delta G correction:	+0.325954 hartree
Grimme's Delta G correction:	+0.325807 hartree
Imaginary Frequency:	215.2 *i*cm^{-1}

Cartesian Coordinates

C	1.48304	1.36736	-0.45578
C	0.09589	1.08712	-0.60204
C	-0.93915	1.55850	0.18498
O	-2.16750	1.12193	0.11652
H	-0.18174	0.50418	-1.47384
O	2.15217	1.14477	-1.60872
C	2.15011	1.60132	0.73476
C	3.54917	1.43781	-1.64788
H	3.72640	2.49830	-1.44598
H	3.87207	1.19785	-2.65903
H	4.10915	0.83359	-0.92641
C	2.55666	-0.64601	1.17389
H	1.56839	1.78293	1.62917
H	3.16935	1.96563	0.74665
C	2.61479	-1.37869	-0.01333
C	1.53006	-2.16835	-0.37693

O	0.58668	-2.55025	0.45847
C	0.89928	-2.32179	1.86591
C	1.37568	-0.90420	2.06790
H	3.47153	-0.24913	1.59601
H	0.54974	-0.22149	1.82017
H	1.62240	-0.73653	3.11891
H	1.65758	-3.06083	2.14579
H	-0.02822	-2.54332	2.39289
O	1.32492	-2.69201	-1.57212
O	-0.85199	2.54193	1.08593
C	-0.09900	3.73378	0.79924
H	-0.76691	4.56946	1.00833
H	0.21666	3.75309	-0.24563
H	0.77496	3.79076	1.44969
Si	-2.95435	-0.36517	-0.32547
C	-2.80660	-1.43886	1.18460
H	-1.75638	-1.61698	1.42990
H	-3.28612	-0.97801	2.05202
H	-3.27157	-2.41551	1.02085
C	-4.68165	0.21778	-0.65750
H	-4.71668	0.90461	-1.50746
H	-5.33985	-0.62542	-0.88640
H	-5.09604	0.73743	0.21024
C	-2.16434	-1.13733	-1.82393
H	-1.18985	-1.58428	-1.61060
H	-2.81263	-1.94498	-2.17999
H	-2.05382	-0.42997	-2.65106
H	1.94216	-2.31237	-2.21407
H	3.44254	-1.28806	-0.70743

10.11 Transition State leading to *Z*-Intermediate

SCF energy:	–1214.031459 hartree
Zero-point correction:	+0.376829 hartree
Enthalpy correction:	+0.401853 hartree
Free energy correction:	+0.324135 hartree
Truhlar's Delta G correction:	+0.327468 hartree
Grimme's Delta G correction:	+0.327427 hartree
Imaginary Frequency:	219.7 *i*cm^{-1}

Cartesian Coordinates

C	-1.49550	1.14750	1.27466
C	-0.61281	0.08638	1.62659
C	0.65310	-0.13392	1.12786
H	-1.03541	-0.67027	2.27253
O	-0.94033	2.21032	0.65812
C	-1.77132	3.33859	0.38855
H	-1.10884	4.09862	-0.02114
H	-2.23856	3.70585	1.30699
H	-2.54917	3.09830	-0.34434
O	1.40587	-1.19096	1.45965
O	1.21799	0.64049	0.24306
C	0.92900	-2.09646	2.46359
H	0.01871	-2.59950	2.12423
H	0.74362	-1.56459	3.39990

H	1.72472	-2.82549	2.59942
C	-2.86173	0.99838	1.44407
H	-3.55548	1.80920	1.25694
H	-3.20598	0.21913	2.11271
C	-2.55933	-1.60557	-0.06980
C	-1.40564	-1.90459	-0.77917
H	-2.86335	-2.29514	0.70823
O	-0.89552	-1.17806	-1.75419
C	-1.38802	0.19448	-1.88680
C	-2.87256	0.29598	-1.60976
H	-1.13600	0.46828	-2.91096
H	-0.78786	0.79839	-1.19919
H	-3.44849	-0.12433	-2.44656
H	-3.15126	1.35197	-1.56036
O	-0.76015	-3.03822	-0.56482
H	0.00183	-3.09516	-1.16301
Si	2.78664	0.47643	-0.51085
C	2.76810	1.97736	-1.60315
C	2.78271	-1.11096	-1.48518
C	4.08195	0.52514	0.81859
H	2.66962	2.89371	-1.01479
H	3.68588	2.05633	-2.19231
H	1.92782	1.93768	-2.30345
H	2.68090	-1.98328	-0.83385
H	3.72138	-1.22079	-2.03694
H	1.97130	-1.12333	-2.21940
H	3.95727	1.39808	1.46524
H	5.08056	0.58615	0.37553
H	4.04993	-0.36992	1.44432
C	-3.26485	-0.44866	-0.37239
H	-4.28746	-0.35025	-0.02836

10.12 Intermediate (*E*)-13-TMS^{+}

SCF energy:	−1214.070679 hartree
Zero-point correction:	+0.380329 hartree
Enthalpy correction:	+0.405334 hartree
Free energy correction:	+0.328322 hartree
Truhlar's Delta G correction:	+0.330636 hartree
Grimme's Delta G correction:	+0.331087 hartree

Cartesian Coordinates

C	-1.32975	1.81371	0.39206
C	0.01592	1.54849	0.28638
C	0.76445	0.70828	1.14512
O	1.85919	0.12864	0.79773
H	0.57557	1.95227	-0.54844
O	-1.93617	2.68313	-0.40989
C	-2.31562	1.06850	1.22051
C	-1.16389	3.43311	-1.35712
H	-0.36725	3.98502	-0.85142
H	-1.86385	4.12346	-1.82059
H	-0.74312	2.76669	-2.11741
C	-2.61856	-0.34700	0.63670
H	-3.23979	1.65136	1.23521

H	-1.96767	0.96287	2.24917
C	-1.52920	-1.34064	0.90398
C	-1.10030	-2.20498	-0.04198
O	-1.60068	-2.31845	-1.27989
C	-2.90057	-1.70711	-1.43642
C	-2.90665	-0.30903	-0.86609
H	-3.53496	-0.65647	1.16072
H	-2.13223	0.26558	-1.39109
H	-3.86297	0.18041	-1.07531
H	-3.63646	-2.35247	-0.94028
H	-3.08721	-1.72303	-2.51030
O	-0.09094	-3.09412	0.08366
O	0.39756	0.58784	2.39906
C	1.17746	-0.27287	3.25872
H	2.18284	0.13143	3.37365
H	1.23157	-1.28022	2.84234
H	0.64852	-0.27338	4.20787
Si	2.64216	-0.04149	-0.80016
C	3.88128	-1.35625	-0.40479
H	4.55527	-1.04998	0.39920
H	4.49346	-1.58940	-1.28034
H	3.38299	-2.28129	-0.10027
C	1.35400	-0.58031	-2.02010
H	0.53361	0.13315	-2.13872
H	0.92352	-1.54924	-1.75479
H	1.82787	-0.69455	-3.00066
C	3.37867	1.62704	-1.15294
H	2.63081	2.39123	-1.38467
H	4.04131	1.55930	-2.02121
H	3.97971	1.98598	-0.31330
H	0.29527	-3.00496	0.96326
H	-1.13221	-1.43493	1.90999

10.13 Intermediate (*Z*)-13-TMS$^+$

SCF energy:	–1214.070026 hartree
Zero-point correction:	+0.379135 hartree
Enthalpy correction:	+0.404893 hartree
Free energy correction:	+0.323420 hartree
Truhlar's Delta G correction:	+0.328820 hartree
Grimme's Delta G correction:	+0.328038 hartree

Cartesian Coordinates

C	-0.44095	0.34531	-1.11884
C	0.43779	1.23982	-0.52712
C	1.73719	0.95122	-0.07788
H	0.06861	2.24792	-0.39776
O	-0.05890	-0.89964	-1.30274
C	-0.93002	-1.86667	-1.92980
H	-1.87382	-1.94386	-1.38629
H	-0.38237	-2.80422	-1.87163
H	-1.09826	-1.59784	-2.97436
O	2.51173	1.85507	0.49814
O	2.26765	-0.21186	-0.19617
C	2.03789	3.19995	0.70687

H	1.82339	3.67819	-0.25069
H	2.85624	3.71364	1.20444
H	1.15470	3.19377	1.34831
C	-1.81655	0.79959	-1.46872
H	-2.27025	0.14067	-2.21250
H	-1.75058	1.79759	-1.91422
C	-2.76037	-0.41201	0.55130
C	-3.91644	-0.97463	0.95016
H	-1.83807	-0.91295	0.82717
O	-5.14525	-0.43533	0.79852
C	-5.13003	0.96940	0.46636
C	-4.15180	1.24681	-0.65089
H	-4.87044	1.52772	1.37509
H	-6.15786	1.20474	0.19013
H	-4.21254	2.30011	-0.94212
H	-4.44175	0.65312	-1.52685
O	-3.97246	-2.17977	1.54306
H	-4.86320	-2.30027	1.89982
Si	3.88941	-0.74939	0.29644
C	5.10202	0.26943	-0.67162
C	3.80343	-2.52038	-0.23911
C	3.99104	-0.52240	2.13564
H	5.10578	1.31405	-0.35193
H	4.88687	0.23789	-1.74334
H	6.11435	-0.12194	-0.53265
H	4.74061	-3.04041	-0.02227
H	3.62247	-2.59937	-1.31447
H	3.00145	-3.05340	0.27860
H	3.14430	-0.99176	2.64408
H	4.01826	0.53307	2.41599
H	4.90129	-0.99093	2.52186
C	-2.73402	0.87355	-0.21823
H	-2.32823	1.68419	0.41032

10.14 Transition State TS2b

SCF energy:	–1214.055683 hartree
Zero-point correction:	+0.379530 hartree
Enthalpy correction:	+0.403304 hartree
Free energy correction:	+0.329327 hartree
Truhlar's Delta G correction:	+0.331868 hartree
Grimme's Delta G correction:	+0.331815 hartree
Imaginary Frequency:	327.2 $i$cm^{-1}

Cartesian Coordinates

C	-0.52378	1.66867	0.11332
C	-0.12177	0.68356	0.95412
C	0.30722	-0.59932	0.40772
C	-1.23318	-1.04317	-0.93087
C	-1.60435	0.23197	-1.66839
C	-0.67747	1.43398	-1.34882
H	-1.06957	2.33836	-1.82051
H	0.30093	1.23673	-1.79260
H	-0.16630	0.76016	2.03452
O	-0.91307	2.89422	0.49621

C	-0.78461	3.21414	1.88361
H	-1.45299	2.59243	2.48899
H	-1.06696	4.26012	1.97707
H	0.25001	3.07001	2.21361
O	0.29247	-1.57599	1.33253
C	0.95296	-2.80627	0.99733
H	0.60161	-3.18778	0.03604
H	0.69025	-3.49942	1.79325
H	2.03607	-2.66669	0.96207
O	1.28340	-0.66339	-0.50491
Si	2.85219	0.08685	-0.32121
C	3.93514	-1.06666	-1.28986
H	4.96654	-0.70365	-1.31720
H	3.59050	-1.15607	-2.32376
H	3.94889	-2.06967	-0.85459
C	2.81116	1.80097	-1.04703
H	2.12251	2.46530	-0.51529
H	2.54445	1.80327	-2.10765
H	3.80929	2.24488	-0.96794
C	3.23953	0.14167	1.50013
H	3.19001	-0.84080	1.97812
H	2.56045	0.81258	2.03578
H	4.25433	0.52349	1.65005
C	-2.19198	-1.60639	-0.08592
H	-0.65567	-1.78655	-1.47304
O	-3.20000	-0.97786	0.46747
C	-3.49488	0.38552	0.00690
C	-3.08397	0.56010	-1.43207
H	-2.98326	1.06235	0.69489
H	-4.56968	0.48338	0.15710
H	-3.30876	1.58894	-1.72936
H	-3.70655	-0.09207	-2.05423
H	-1.48056	0.04087	-2.73882
O	-2.05887	-2.85453	0.31429
H	-2.74773	-3.06855	0.96466

10.15 Protonated Cycloadduct *exo*-I1-H^+

SCF energy:	–1214.080493 hartree
Zero-point correction:	+0.381320 hartree
Enthalpy correction:	+0.404977 hartree
Free energy correction:	+0.330999 hartree
Truhlar's Delta G correction:	+0.333530 hartree
Grimme's Delta G correction:	+0.333525 hartree

Cartesian Coordinates

C	-1.06906	2.23903	-0.16715
C	0.07624	1.54207	-0.30070
C	0.02917	0.07702	-0.03181
C	-1.21202	-0.54488	-0.74008
C	-2.53349	0.17624	-0.38154
C	-2.32148	1.55489	0.27025
H	-2.28417	1.43027	1.36114
H	-3.18107	2.19690	0.06411
H	1.01085	1.97732	-0.62518

O	-1.22786	3.55764	-0.36453
C	-0.05618	4.29129	-0.71906
H	-0.36464	5.33088	-0.80377
H	0.34245	3.94038	-1.67694
H	0.71108	4.18869	0.05678
O	-0.16906	-0.30053	1.32846
C	0.36669	0.55797	2.33096
H	0.29494	0.00403	3.26688
H	-0.20504	1.48948	2.40997
H	1.41649	0.80898	2.14428
O	1.17578	-0.57682	-0.56853
Si	2.84816	-0.51622	-0.08900
C	3.42195	1.24806	0.09694
H	4.48230	1.23942	0.37091
H	2.89807	1.81272	0.87358
H	3.33889	1.80329	-0.84229
C	3.05391	-1.51750	1.46890
H	2.56048	-2.49088	1.38314
H	4.11765	-1.71107	1.64116
H	2.66490	-1.02703	2.36386
C	3.66008	-1.35518	-1.53461
H	3.32820	-2.39185	-1.64793
H	4.74562	-1.38049	-1.40058
H	3.45520	-0.83128	-2.47211
C	-1.24034	-2.02538	-0.52549
H	-1.01105	-0.44101	-1.81482
O	-2.28570	-2.72545	-0.28655
C	-3.59059	-2.06061	-0.08647
C	-3.39493	-0.70255	0.51584
H	-4.12464	-2.75986	0.55415
H	-4.06422	-2.03935	-1.07140
H	-2.92654	-0.80106	1.50341
H	-4.37699	-0.24493	0.66812
H	-3.07203	0.33086	-1.32391
O	-0.16401	-2.71126	-0.63418
H	0.59610	-2.03871	-0.70967

10.16 Protonated Cycloadduct *endo*-I1-H$^+$

SCF energy:	–1214.083171 hartree
Zero-point correction:	+0.380460 hartree
Enthalpy correction:	+0.404353 hartree
Free energy correction:	+0.329417 hartree
Truhlar's Delta G correction:	+0.332377 hartree
Grimme's Delta G correction:	+0.332229 hartree

Cartesian Coordinates

C	1.44739	1.73013	-0.28151
C	0.16061	1.32712	-0.25687
C	-0.25659	0.04459	0.37459
C	0.92557	-0.94742	0.49336
C	2.16598	-0.28084	1.09237
C	2.59197	0.93315	0.26309
H	3.21698	0.63720	-0.59165
H	3.22881	1.59230	0.86236

H	-0.63681	1.94057	-0.66068
O	1.89199	2.87752	-0.82518
C	0.92258	3.73631	-1.42298
H	0.41085	3.22790	-2.24692
H	1.47516	4.59330	-1.80155
H	0.18778	4.06595	-0.68043
O	-1.26160	-0.59804	-0.40810
O	-0.81415	0.16878	1.67867
C	1.27481	-1.65197	-0.78239
H	0.57774	-1.74776	1.16460
O	2.44234	-2.12237	-1.03293
C	3.61004	-1.91440	-0.14612
C	3.25633	-1.34098	1.20348
H	4.26091	-1.26898	-0.73931
H	4.06445	-2.90278	-0.09144
H	4.17237	-0.92421	1.63355
H	2.92441	-2.13533	1.87921
H	1.89383	0.04795	2.10040
O	0.40982	-1.87708	-1.69263
C	-0.65448	1.39447	2.38513
H	-1.07970	2.24642	1.84361
H	-1.19516	1.25521	3.32159
H	0.39683	1.61351	2.60777
C	-3.38871	1.39000	-0.09974
H	-3.06902	1.91615	-1.00425
H	-4.47201	1.52292	-0.01717
H	-2.94211	1.88813	0.76571
C	-3.61089	-1.23121	-1.72341
H	-3.31508	-2.28299	-1.78612
H	-4.70410	-1.20388	-1.75455
H	-3.24556	-0.72289	-2.62044
C	-3.51354	-1.34299	1.35917
H	-4.59952	-1.48039	1.35934
H	-3.05972	-2.33736	1.40569
H	-3.23841	-0.80589	2.26950
Si	-2.98013	-0.42635	-0.16811
H	-0.46487	-1.43250	-1.37289

11. Calculated Energies and Geometries for the $AlMe_3$-Catalysed Reaction

11.1 Trimethylaluminium $AlMe_3$

SCF energy:	–362.172934 hartree
Zero-point correction:	+0.106107 hartree
Enthalpy correction:	+0.115789 hartree
Free energy correction:	+0.071181 hartree
Truhlar's Delta G correction:	+0.074646 hartree
Grimme's Delta G correction:	+0.073758 hartree

Cartesian Coordinates

C	1.91736	-0.40742	-0.00087
C	-1.31192	-1.45583	0.00031
C	-0.60579	1.86335	0.00077
Al	0.00040	-0.00035	-0.00038
H	2.18747	-1.06485	-0.83604
H	2.20503	-0.95034	0.90790
H	2.55500	0.48013	-0.06265
H	-1.97837	-1.38923	0.86889
H	-0.86304	-2.45398	0.00896
H	-1.96585	-1.39988	-0.87861
H	-0.26341	2.39083	-0.89811
H	-0.18595	2.41932	0.84781
H	-1.69395	1.97191	0.04559

11.2 Lactone-$AlMe_3$ Complex 7-$AlMe_3$

SCF energy:	–706.815025 hartree
Zero-point correction:	+0.211756 hartree
Enthalpy correction:	+0.228327 hartree
Free energy correction:	+0.167149 hartree
Truhlar's Delta G correction:	+0.170737 hartree
Grimme's Delta G correction:	+0.170366 hartree

Cartesian Coordinates

C	-3.14497	1.04398	-0.06140
C	-1.90161	1.46586	0.22080
C	-0.77910	0.54268	0.12997
O	-1.00294	-0.76102	0.01887
C	-2.34488	-1.24886	0.29457
C	-3.40553	-0.39330	-0.35540
H	-2.46104	-1.25992	1.38399
H	-2.34021	-2.27544	-0.06931
H	-4.38799	-0.69812	0.01773
H	-3.43636	-0.54525	-1.44332
H	-3.96572	1.75477	-0.11167
H	-1.65506	2.50294	0.41300
O	0.39235	0.94763	0.09254
Al	2.08829	-0.10848	-0.04524
C	1.86238	-1.00322	-1.79994
C	1.96556	-1.24636	1.57514

C	3.37295	1.39312	0.02251
H	3.28629	1.97408	0.94988
H	3.23133	2.09836	-0.80670
H	4.41582	1.05563	-0.03393
H	1.11961	-1.81031	-1.77033
H	2.79892	-1.45228	-2.15500
H	1.54444	-0.30282	-2.58392
H	1.22552	-2.05001	1.47192
H	1.69009	-0.66766	2.46715
H	2.92252	-1.72996	1.80996

11.3 Brassard-$AlMe_3$ Complex via OTMS-Group

SCF energy:	–1231.223636 hartree
Zero-point correction:	+0.365909 hartree
Enthalpy correction:	+0.394020 hartree
Free energy correction:	+0.309431 hartree
Truhlar's Delta G correction:	+0.313511 hartree
Grimme's Delta G correction:	+0.313369 hartree

Cartesian Coordinates

C	2.98086	-0.04817	0.04979
C	3.56105	-0.35819	1.22852
H	2.94676	-0.53030	2.09946
H	4.63324	-0.45286	1.33586
C	1.56449	0.09647	-0.23563
O	3.67883	0.18538	-1.11031
C	0.52751	-0.06180	0.60768
H	1.30863	0.31809	-1.26546
O	-0.77619	0.07306	0.16690
O	0.66547	-0.33029	1.91495
C	5.08623	0.07413	-1.03761
H	5.50572	0.79496	-0.32433
H	5.46053	0.28865	-2.03822
H	5.38980	-0.93706	-0.73923
C	-0.48700	-0.74224	2.65552
H	-0.92237	-1.65204	2.23076
H	-1.24787	0.04373	2.68407
H	-0.12211	-0.93914	3.66182
Si	-1.37319	1.72056	-0.08706
Al	-1.64628	-1.64573	-0.64384
C	-1.84619	-1.05405	-2.51983
C	-0.18983	-2.93361	-0.29132
C	-3.28348	-1.81911	0.45923
H	0.67002	-2.77286	-0.95317
H	0.20781	-2.93253	0.73180
H	-0.54261	-3.95716	-0.47579
H	-3.47871	-2.87102	0.70484
H	-3.21714	-1.29091	1.41945
H	-4.18360	-1.44120	-0.04077
C	-0.70952	2.33421	-1.70921
C	-3.21829	1.53182	-0.06466
C	-0.71557	2.70472	1.34901
H	-0.92606	1.63848	-2.52456

H	-1.17497	3.29330	-1.95871
H	0.37146	2.49424	-1.67026
H	-2.53100	-0.20938	-2.67004
H	-0.88279	-0.77195	-2.96520
H	-2.23818	-1.87160	-3.13881
H	-0.97966	3.75982	1.22632
H	-1.12657	2.37529	2.30762
H	0.37565	2.64824	1.40945
H	-3.57002	1.12026	0.88542
H	-3.68718	2.51174	-0.20030
H	-3.57900	0.87938	-0.86447

11.4 Brassard-$AlMe_3$ Complex via OMe-Group (Enol Ether)

SCF energy:	−1231.227027 hartree
Zero-point correction:	+0.364915 hartree
Enthalpy correction:	+0.393722 hartree
Free energy correction:	+0.306747 hartree
Truhlar's Delta G correction:	+0.311260 hartree
Grimme's Delta G correction:	+0.311331 hartree

Cartesian Coordinates

C	-0.08367	0.77923	0.32356
C	1.12123	1.55944	0.26665
C	-1.35314	1.22040	0.09347
C	1.37578	2.83252	-0.06995
H	2.39397	3.20119	-0.04353
H	0.57885	3.49109	-0.38550
O	2.26822	0.77886	0.63062
C	2.39979	0.60644	2.05364
H	1.52026	0.09508	2.46053
H	3.29287	0.00054	2.21442
H	2.51633	1.58699	2.52130
O	-2.44263	0.46300	0.09560
O	-1.57537	2.52160	-0.15133
H	0.04342	-0.27722	0.53025
C	-2.92164	2.93378	-0.40470
H	-2.86055	4.00628	-0.58098
H	-3.32616	2.43093	-1.28600
H	-3.56331	2.73018	0.45535
Si	-2.54302	-1.26181	0.05221
C	-1.87568	-1.95143	1.65199
C	-4.37894	-1.52202	-0.08533
C	-1.61722	-1.84180	-1.45552
H	-2.15802	-3.00500	1.74838
H	-0.78557	-1.90464	1.72674
H	-2.29631	-1.41911	2.51054
H	-4.90511	-1.10827	0.77959
H	-4.78077	-1.04358	-0.98294
H	-4.62092	-2.58758	-0.14100
H	-2.09285	-1.47612	-2.37043
H	-1.59945	-2.93519	-1.50354
H	-0.57772	-1.49901	-1.46203
Al	2.83881	-0.80473	-0.51999

C	1.87987	-2.30858	0.36004
C	2.18844	-0.20724	-2.28395
C	4.78020	-0.74583	-0.14806
H	2.34352	-0.97522	-3.05270
H	1.11503	0.02316	-2.28457
H	2.70220	0.69652	-2.63449
H	5.33501	-1.42813	-0.80484
H	5.20453	0.25280	-0.31176
H	5.03917	-1.03926	0.87773
H	2.36462	-3.26372	0.11757
H	1.89382	-2.24148	1.45726
H	0.82905	-2.42157	0.06333

11.5 Brassard-$AlMe_3$ Complex via OMe-Group (Silyl Ketene Acetal)

SCF energy:	–1231.224975 hartree
Zero-point correction:	+0.365501 hartree
Enthalpy correction:	+0.393859 hartree
Free energy correction:	+0.308763 hartree
Truhlar's Delta G correction:	+0.312909 hartree
Grimme's Delta G correction:	+0.312882 hartree

Cartesian Coordinates

C	1.40521	2.11645	-0.16779
C	0.06507	1.60722	-0.39098
C	-0.33411	0.37425	-0.76043
H	-0.73429	2.31311	-0.18937
C	1.61427	3.36269	0.31244
H	0.76192	3.99266	0.53387
H	2.59916	3.77079	0.49320
O	0.57708	-0.64478	-0.98576
O	-1.59204	-0.01917	-0.94351
O	2.39336	1.23245	-0.47798
C	0.25968	-1.52822	-2.08878
H	1.11823	-2.19380	-2.17822
H	0.14228	-0.93421	-2.99666
H	-0.64752	-2.09547	-1.87613
Si	-2.91048	0.24169	0.13502
C	-2.23479	0.17344	1.86859
H	-3.04907	0.19929	2.59956
H	-1.57462	1.01984	2.08184
H	-1.66055	-0.74264	2.04257
C	-4.04777	-1.16894	-0.28026
H	-3.56961	-2.13244	-0.08069
H	-4.32944	-1.14823	-1.33702
H	-4.96878	-1.12527	0.30844
C	-3.67722	1.89593	-0.25560
H	-3.01811	2.73083	0.00042
H	-4.60387	2.03193	0.31138
H	-3.92616	1.97976	-1.31733
C	3.71778	1.63241	-0.17392
H	4.35399	0.78337	-0.42408
H	3.81934	1.87015	0.89237
H	4.01380	2.50664	-0.76646

Al	1.52578	-1.64399	0.60173
C	1.55235	-0.37396	2.11167
C	3.20733	-2.07559	-0.34799
C	0.16643	-3.08582	0.74372
H	2.51321	0.14674	2.20420
H	1.37733	-0.89699	3.06119
H	0.78290	0.40455	2.04509
H	-0.85967	-2.69001	0.75460
H	0.27903	-3.65685	1.67437
H	0.21107	-3.81708	-0.07383
H	3.38124	-1.36967	-1.17112
H	3.22440	-3.08360	-0.78069
H	4.08282	-2.00239	0.30976

11.6 Transition State TS3a leading to *E*-Intermediate

SCF energy:	–1575.838533 hartree
Zero-point correction:	+0.471484 hartree
Enthalpy correction:	+0.505859 hartree
Free energy correction:	+0.407627 hartree
Truhlar's Delta G correction:	+0.412647 hartree
Grimme's Delta G correction:	+0.412897 hartree
Imaginary Frequency:	415.5 $i$cm^{-1}

Cartesian Coordinates

C	3.22972	0.12269	0.06075
C	2.19125	1.05778	0.25463
C	1.06076	0.91377	1.03976
O	0.04200	1.73348	1.02330
H	2.24219	1.95579	-0.35030
O	4.14454	0.59664	-0.82649
C	3.23514	-1.20573	0.49100
C	5.26803	-0.21530	-1.14182
H	5.83704	-0.47035	-0.24146
H	5.88589	0.38372	-1.80892
H	4.96721	-1.13474	-1.65518
C	1.87508	-2.14277	-0.87573
H	2.67342	-1.42915	1.38665
H	4.12861	-1.80452	0.34847
C	0.78112	-1.31983	-1.15287
C	-0.45551	-1.48718	-0.50877
O	-0.65000	-2.45557	0.41311
C	0.50779	-3.15549	0.92264
C	1.56705	-3.40794	-0.12513
H	2.65102	-2.20953	-1.63440
H	2.45155	-3.82793	0.36417
H	1.21903	-4.16102	-0.84507
H	0.11103	-4.08247	1.33961
H	0.91436	-2.55421	1.74493
O	-1.46621	-0.77274	-0.76999
O	0.97657	-0.06398	1.94203
C	-0.25379	-0.17674	2.68660
H	-0.42537	0.72147	3.28195
H	-0.10876	-1.03887	3.33597

H	-1.09548	-0.34416	2.01334
Si	-0.44838	2.94320	-0.13258
C	-0.34861	2.22735	-1.84343
H	-0.82325	2.91409	-2.55236
H	-0.88646	1.27546	-1.89139
H	0.67957	2.06481	-2.17934
C	-2.18570	3.26718	0.42663
H	-2.78464	2.35319	0.36492
H	-2.67124	4.02302	-0.19776
H	-2.21198	3.61809	1.46235
C	0.67878	4.40680	0.11870
H	0.67911	4.73243	1.16289
H	0.34006	5.25224	-0.48862
H	1.71370	4.19792	-0.16724
Al	-3.35341	-0.90834	-0.42565
C	-3.99315	0.44362	-1.73707
C	-3.62534	-0.41422	1.48710
C	-3.80416	-2.79299	-0.85827
H	-5.08125	0.58750	-1.70250
H	-3.75342	0.14451	-2.76696
H	-3.54722	1.43782	-1.59513
H	-4.88591	-2.95458	-0.95525
H	-3.44913	-3.49223	-0.09021
H	-3.35872	-3.11553	-1.80956
H	-3.17575	0.54273	1.78753
H	-3.24814	-1.17801	2.18162
H	-4.70017	-0.31985	1.69590
H	0.83858	-0.51251	-1.87301

11.7 Transition State leading to *Z*-Intermediate

SCF energy:	–1575.834229 hartree
Zero-point correction:	+0.471512 hartree
Enthalpy correction:	+0.505982 hartree
Free energy correction:	+0.405972 hartree
Truhlar's Delta G correction:	+0.412620 hartree
Grimme's Delta G correction:	+0.412207 hartree
Imaginary Frequency:	427.9 *i*cm^{-1}

Cartesian Coordinates

C	0.97686	2.31427	0.80158
C	0.79187	0.99638	1.28626
C	1.45145	-0.14853	0.88401
H	0.04225	0.89407	2.05906
O	1.80608	2.45770	-0.25269
C	2.10288	3.77328	-0.70711
H	2.48997	4.39332	0.10778
H	1.22213	4.25658	-1.14386
H	2.86519	3.65251	-1.47531
O	1.18091	-1.36316	1.36877
O	2.42416	-0.15135	0.01414
C	0.02457	-1.54412	2.19878
H	-0.87002	-1.16349	1.69529
H	0.16548	-1.04862	3.16360
H	-0.06405	-2.61977	2.33936

C	0.18464	3.35717	1.29651
H	0.40799	4.38008	1.01102
H	-0.15430	3.24282	2.32009
C	-2.30187	1.90819	1.02000
C	-2.51940	0.79267	0.19509
H	-2.58792	1.79723	2.06024
O	-2.22413	0.82467	-1.12361
C	-1.31798	1.85141	-1.56885
C	-1.64695	3.19988	-0.96616
H	-1.40860	1.85389	-2.65626
H	-0.30013	1.53022	-1.31136
H	-2.60991	3.55726	-1.35699
H	-0.89793	3.93380	-1.28012
O	-3.06660	-0.27618	0.60081
Al	-3.09236	-1.97104	-0.31843
Si	3.42829	-1.49499	-0.45587
C	4.36517	-2.04042	1.05876
C	4.52118	-0.66484	-1.71092
C	2.38601	-2.84350	-1.19224
H	4.89422	-1.20433	1.52518
H	5.11157	-2.79727	0.79765
H	3.69611	-2.47852	1.80384
H	5.26355	-1.36164	-2.11132
H	5.05898	0.17960	-1.27070
H	3.93460	-0.28550	-2.55247
H	1.80892	-3.36867	-0.42741
H	1.67263	-2.45070	-1.92245
H	3.02121	-3.57475	-1.70301
C	-1.75289	3.09812	0.53071
H	-2.00926	4.01834	1.05048
C	-3.54434	-3.16376	1.20717
C	-4.48106	-1.79700	-1.72454
C	-1.21315	-2.17907	-0.97820
H	-4.53212	-2.94455	1.63383
H	-3.55753	-4.22457	0.92268
H	-2.82357	-3.07612	2.03273
H	-4.22786	-1.01502	-2.45256
H	-5.46632	-1.54071	-1.31296
H	-4.61756	-2.72469	-2.29642
H	-1.16973	-2.28670	-2.07118
H	-0.57984	-1.31035	-0.74332
H	-0.69875	-3.05450	-0.55780

11.8 Intermediate (*E*)-13-AlMe$_3$

SCF energy:	–1575.861071 hartree
Zero-point correction:	+0.474498 hartree
Enthalpy correction:	+0.508464 hartree
Free energy correction:	+0.411675 hartree
Truhlar's Delta G correction:	+0.416221 hartree
Grimme's Delta G correction:	+0.416515 hartree

Cartesian Coordinates

C	-3.15204	-0.61231	0.21683
C	-2.45136	0.56625	0.16532

C	-1.47107	0.98229	1.10576
O	-0.53040	1.81446	0.82543
H	-2.56639	1.22732	-0.68580
O	-4.09632	-0.91687	-0.68565
C	-2.83024	-1.77225	1.07835
C	-4.41455	0.03503	-1.70046
H	-3.55687	0.19090	-2.36449
H	-4.72154	0.98777	-1.25807
H	-5.23860	-0.39783	-2.26309
C	-1.46600	-2.43575	0.65835
H	-3.63733	-2.50292	0.97794
H	-2.75728	-1.48420	2.12866
C	-0.29143	-1.63149	1.08767
C	0.74990	-1.28513	0.26600
O	0.84916	-1.79840	-1.00347
C	0.04990	-2.96570	-1.23879
C	-1.38754	-2.71145	-0.84517
H	-1.48634	-3.39989	1.19047
H	-1.73426	-1.84042	-1.41739
H	-2.02540	-3.55483	-1.13152
H	0.47966	-3.80155	-0.66780
H	0.15959	-3.18059	-2.30383
O	1.69114	-0.45841	0.56682
O	-1.62854	0.68434	2.37966
C	-0.52625	1.00604	3.25913
H	-0.45679	2.08672	3.38724
H	0.40624	0.61873	2.84003
H	-0.76830	0.52101	4.20186
Si	0.08277	2.51717	-0.67430
C	1.75941	3.06683	-0.12752
H	2.33934	2.20418	0.21500
H	2.31440	3.53515	-0.94537
H	1.69397	3.78569	0.69408
C	0.07461	1.26997	-2.04384
H	-0.93289	0.98704	-2.36286
H	0.62747	0.36454	-1.78236
H	0.57978	1.71284	-2.90960
C	-1.10281	3.92065	-0.99721
H	-1.15776	4.60549	-0.14649
H	-2.11756	3.56852	-1.20961
H	-0.77642	4.50077	-1.86604
Al	3.47735	-0.57673	-0.07721
C	4.43978	0.49096	1.30647
C	3.51451	0.23178	-1.90331
C	3.90300	-2.52514	-0.04107
H	5.52086	0.53209	1.11442
H	4.32188	0.05127	2.30684
H	4.10650	1.53521	1.38627
H	4.97957	-2.73170	0.02600
H	3.53719	-3.04629	-0.93621
H	3.43985	-3.02551	0.82226
H	4.53612	0.24498	-2.30862
H	3.15832	1.27069	-1.94392
H	2.90463	-0.34111	-2.61585
H	-0.20317	-1.35133	2.13014

11.9 Intermediate (*Z*)-13-AlMe$_3$

SCF energy:	–1575.854433 hartree
Zero-point correction:	+0.474163 hartree
Enthalpy correction:	+0.508424 hartree
Free energy correction:	+0.409732 hartree
Truhlar's Delta G correction:	+0.415613 hartree
Grimme's Delta G correction:	+0.415291 hartree

Cartesian Coordinates

C	-0.33503	1.90754	0.81810
C	-0.68118	0.60844	1.15539
C	-1.86458	-0.05797	0.80687
H	0.08357	0.03664	1.66505
O	-1.21925	2.66288	0.18242
C	-0.92247	4.03981	-0.09899
H	-0.00170	4.13914	-0.67621
H	-0.85250	4.60980	0.83076
H	-1.76694	4.39430	-0.68595
O	-2.12212	-1.30024	1.16984
O	-2.79405	0.49183	0.09902
C	-1.15735	-2.04587	1.94629
H	-0.98840	-1.55042	2.90491
H	-0.22388	-2.14542	1.38800
H	-1.61513	-3.02042	2.09853
C	1.03259	2.40031	1.09780
H	1.45047	1.82733	1.93015
H	1.01668	3.45671	1.38789
C	2.10579	0.78985	-0.54099
C	3.29076	0.12363	-0.67604
H	1.20678	0.20034	-0.67733
O	4.49805	0.78169	-0.63009
C	4.40212	2.20296	-0.71845
C	3.37518	2.74972	0.24885
H	4.14144	2.47894	-1.75182
H	5.40630	2.57718	-0.50578
H	3.39677	3.84569	0.25194
H	3.63632	2.41505	1.26139
O	3.44732	-1.14565	-0.86697
Al	2.33444	-2.46059	-0.15408
Si	-4.19404	-0.31096	-0.61594
C	-5.29037	-0.87411	0.77550
C	-3.54020	-1.68376	-1.67994
C	-4.91230	1.10293	-1.58003
H	-4.84021	-1.69580	1.33714
H	-6.25065	-1.22383	0.38402
H	-5.50024	-0.05820	1.47293
H	-4.34338	-2.10697	-2.29106
H	-3.10961	-2.49311	-1.08540
H	-2.76313	-1.32398	-2.36044
H	-5.17903	1.93528	-0.92287
H	-4.20061	1.47477	-2.32218
H	-5.81759	0.79669	-2.11193
C	1.99546	2.21611	-0.12924
H	1.58602	2.84562	-0.93996

C	2.29905	-2.03574	1.81079
C	0.53419	-2.36963	-1.05539
C	3.26476	-4.16052	-0.58497
H	1.87163	-1.04770	2.04294
H	1.76909	-2.76821	2.43625
H	3.33181	-2.00691	2.18587
H	-0.08714	-1.49324	-0.81519
H	-0.08202	-3.25396	-0.83033
H	0.65363	-2.35823	-2.14846
H	4.27687	-4.20085	-0.16111
H	2.73138	-5.04270	-0.20475
H	3.37425	-4.30874	-1.66778

11.10 Transition State TS3b

SCF energy:	−1575.859700 hartree
Zero-point correction:	+0.474324 hartree
Enthalpy correction:	+0.507382 hartree
Free energy correction:	+0.413056 hartree
Truhlar's Delta G correction:	+0.417328 hartree
Grimme's Delta G correction:	+0.417455 hartree
Imaginary Frequency:	272.0 *i*cm^{-1}

Cartesian Coordinates

C	-3.27500	-0.74745	-0.03660
C	-2.57631	0.36163	0.32176
C	-1.24691	0.27808	0.88723
C	-0.50008	-1.05733	-0.85386
C	-1.43972	-2.22913	-0.92054
C	-2.68897	-2.10879	0.01476
H	-2.38604	-2.34526	1.03827
H	-3.44244	-2.84022	-0.28904
H	-2.93203	1.36202	0.10506
O	-4.49810	-0.71950	-0.59903
C	-5.10453	0.55638	-0.78411
H	-5.20618	1.07956	0.17293
H	-6.08718	0.36334	-1.20963
H	-4.51551	1.17055	-1.47551
O	-1.08957	-0.54754	1.93317
C	0.16879	-0.47114	2.62938
H	0.24501	0.47486	3.16965
H	1.00787	-0.55587	1.93713
H	0.16066	-1.30677	3.32752
O	-0.43356	1.30169	0.87977
Si	-0.24496	2.72568	-0.09621
C	1.32155	3.41065	0.61631
H	1.20303	3.65182	1.67684
H	1.63684	4.31986	0.09622
H	2.13034	2.67872	0.52651
C	-0.12151	2.27694	-1.89374
H	-1.05546	1.85876	-2.28346

H	0.68649	1.56038	-2.06472
H	0.09706	3.18046	-2.47315
C	-1.72532	3.81827	0.23601
H	-2.60867	3.52181	-0.33881
H	-1.49377	4.84972	-0.04934
H	-1.99549	3.82761	1.29604
C	0.84988	-1.21060	-0.51383
H	-0.66623	-0.24733	-1.55403
O	1.29619	-2.30240	0.15110
C	0.30141	-3.28336	0.51194
C	-0.67760	-3.51892	-0.61414
H	-0.21422	-2.93124	1.41629
H	0.87179	-4.17717	0.76962
H	-1.36121	-4.32892	-0.33581
H	-0.12640	-3.85083	-1.50076
H	-1.83909	-2.29106	-1.94128
O	1.71914	-0.31489	-0.74432
Al	3.57083	-0.12539	-0.29576
C	4.01052	1.42976	-1.45847
C	4.46121	-1.82030	-0.82443
C	3.56682	0.22967	1.66942
H	3.93166	1.15623	-2.52023
H	5.55425	-1.72456	-0.87137
H	4.13965	-2.16034	-1.81873
H	4.24438	-2.63972	-0.12705
H	4.55809	0.55684	2.01204
H	3.32690	-0.67116	2.25253
H	2.86169	1.01092	1.98726
H	5.03509	1.79679	-1.31179
H	3.34960	2.29565	-1.31543

11.11 Cycloadduct *exo*-I1-$AlMe_3$

SCF energy:	–1575.898583 hartree
Zero-point correction:	+0.476574 hartree
Enthalpy correction:	+0.509896 hartree
Free energy correction:	+0.414341 hartree
Truhlar's Delta G correction:	+0.419100 hartree
Grimme's Delta G correction:	+0.419123 hartree

Cartesian Coordinates

C	-3.50022	-0.54514	-0.48526
C	-2.71921	0.32717	0.17101
C	-1.21871	0.32319	0.11478
C	-0.65358	-0.79014	-0.82661
C	-1.54499	-2.03459	-0.91704
C	-2.96615	-1.66577	-1.31230
H	-3.62722	-2.53351	-1.21184
H	-3.00687	-1.37657	-2.37093
H	-3.14131	1.16714	0.71127
O	-4.85657	-0.51584	-0.51749
C	-5.49123	0.53912	0.19054
H	-5.24964	0.49341	1.25917
H	-6.56153	0.39877	0.04891
H	-5.18827	1.51521	-0.20511

O	-0.61984	0.02461	1.38522
C	-1.11744	0.76762	2.48400
H	-1.19805	1.83637	2.24522
H	-0.39808	0.63970	3.29441
H	-2.09904	0.39682	2.80706
O	-0.85793	1.59769	-0.31595
Si	0.48426	2.63110	-0.26149
C	1.36721	2.55505	-1.90457
H	1.83281	1.58308	-2.07935
H	0.66864	2.75893	-2.72275
H	2.15842	3.31126	-1.95025
C	-0.32083	4.31020	-0.06997
H	-1.02011	4.51364	-0.88652
H	0.42761	5.10953	-0.06750
H	-0.87908	4.37918	0.86957
C	1.60773	2.32762	1.19770
H	1.09788	2.53390	2.14402
H	2.46659	3.00576	1.13789
H	1.99624	1.30812	1.23984
C	0.72880	-1.15563	-0.36753
H	-0.54756	-0.32730	-1.81304
O	0.96529	-2.25504	0.30943
C	-0.05126	-3.24217	0.65344
C	-1.46770	-2.79029	0.40436
H	0.14657	-3.47737	1.70061
H	0.20710	-4.11640	0.04748
H	-1.82474	-2.15693	1.22249
H	-2.10393	-3.68108	0.37716
H	-1.12988	-2.68134	-1.70284
O	1.69845	-0.42487	-0.62302
Al	3.58415	-0.77119	-0.02959
C	4.44171	0.84790	-0.77857
C	3.99103	-2.47317	-0.96307
C	3.38488	-0.84959	1.94959
H	5.51664	0.87380	-0.55614
H	3.52790	-3.34271	-0.47976
H	5.06976	-2.67311	-1.00061
H	3.64792	-2.46584	-2.00670
H	3.93834	-0.05514	2.46669
H	3.73758	-1.80440	2.36122
H	2.33553	-0.75056	2.26144
H	4.35090	0.91022	-1.87096
H	4.01992	1.77677	-0.37311

11.12 Cycloadduct *endo*-I1-$AlMe_3$

SCF energy:	–1575.899258 hartree
Zero-point correction:	+0.476295 hartree
Enthalpy correction:	+0.509823 hartree
Free energy correction:	+0.413185 hartree
Truhlar's Delta G correction:	+0.418364 hartree
Grimme's Delta G correction:	+0.418480 hartree

Cartesian Coordinates

C	3.25397	-1.06933	0.27588

C	2.53445	0.05602	0.42158
C	1.03949	0.08468	0.50199
C	0.41847	-1.34654	0.57397
C	1.18741	-2.38307	-0.24908
C	2.64801	-2.43127	0.17979
H	3.23535	-3.03149	-0.52382
H	2.74158	-2.93244	1.15298
H	3.00786	1.03029	0.48020
O	4.60746	-1.12986	0.20815
C	5.30975	0.09820	0.34180
H	5.04620	0.79095	-0.46629
H	6.36825	-0.14952	0.28351
H	5.09038	0.57152	1.30634
O	0.47676	0.72361	-0.61449
O	0.60204	0.86656	1.61608
C	-1.00838	-1.23735	0.13197
H	0.40201	-1.64678	1.62766
O	-1.38140	-1.62709	-1.06614
C	-0.46817	-2.12407	-2.08972
C	0.99774	-2.05981	-1.72673
H	-0.71099	-1.53228	-2.97559
H	-0.79850	-3.15302	-2.26007
H	1.40799	-1.07017	-1.94870
H	1.52943	-2.78468	-2.35199
H	0.74177	-3.36820	-0.05232
O	-1.87647	-0.78061	0.88873
C	1.18060	0.56869	2.87027
H	0.69515	1.22646	3.59206
H	0.99797	-0.47219	3.17433
H	2.26353	0.74976	2.87839
C	-1.22529	2.98332	0.27691
H	-2.09763	2.33882	0.13013
H	-0.98667	2.97861	1.34332
H	-1.51677	4.00023	-0.00814
C	1.77900	3.33942	-0.27079
H	2.64343	3.02653	-0.86591
H	1.64118	4.41356	-0.43516
H	2.02110	3.20095	0.78809
C	-0.12526	2.57494	-2.57162
H	-1.00042	1.98270	-2.85866
H	-0.33044	3.61421	-2.84673
H	0.72317	2.23083	-3.17141
Si	0.22364	2.40744	-0.74468
Al	-3.76697	-0.36694	0.41396
C	-4.21969	0.75395	1.98063
H	-5.22521	1.18909	1.91443
H	-3.52435	1.59531	2.10074
H	-4.18812	0.18406	2.91848
C	-4.55599	-2.18505	0.35280
H	-4.40042	-2.74463	1.28460
H	-4.13220	-2.79002	-0.45995
H	-5.64084	-2.16162	0.18657
C	-3.57977	0.56236	-1.33797
H	-3.90501	-0.07266	-2.17290
H	-2.53288	0.82316	-1.54563
H	-4.15132	1.49735	-1.40329

12. Calculated Energies and Geometries for the Formation of the Active Catalyst and its Brønsted Acidity

12.1 Tf_2CH_2-$AlMe_3$ Complex

SCF energy:	−2174.156859 hartree
Zero-point correction:	+0.184841 hartree
Enthalpy correction:	+0.210341 hartree
Free energy correction:	+0.129545 hartree
Truhlar's Delta G correction:	+0.134078 hartree
Grimme's Delta G correction:	+0.133935 hartree

Cartesian Coordinates

C	-0.33250	-0.48323	0.33482
S	0.56459	0.53397	-0.86512
C	0.87704	2.18118	0.05551
F	2.14845	2.50199	-0.12413
F	0.63403	2.00197	1.35273
F	0.09429	3.11709	-0.44026
S	-2.00636	0.07466	0.78447
H	0.25599	-0.52000	1.25863
H	-0.35909	-1.49164	-0.09306
C	-3.07314	-0.95044	-0.38106
F	-4.34061	-0.65596	-0.13301
O	-2.15566	1.46929	0.40010
O	-2.27462	-0.42645	2.12101
O	-0.22352	0.80131	-2.04885
O	1.89557	-0.09545	-0.98199
F	-2.85042	-2.24466	-0.14950
F	-2.77281	-0.66931	-1.64348
Al	3.00665	-1.44388	0.24192
C	1.83659	-3.02909	0.04432
H	1.06433	-3.12374	0.81872
H	2.42738	-3.95170	0.10004
H	1.33428	-3.06546	-0.93215
C	2.90473	-0.46190	1.95762
H	3.64599	-0.89042	2.64519
H	1.95448	-0.51149	2.50633
H	3.16870	0.59898	1.86815
C	4.66805	-1.34058	-0.79211
H	4.53045	-1.67035	-1.82831
H	5.46013	-1.96802	-0.36636
H	5.06423	-0.31913	-0.82964

12.2 Aluminum Methide I2

SCF energy:	−2133.678186 hartree
Zero-point correction:	+0.138203 hartree
Enthalpy correction:	+0.160516 hartree
Free energy correction:	+0.086515 hartree
Truhlar's Delta G correction:	+0.090408 hartree
Grimme's Delta G correction:	+0.090286 hartree

Cartesian Coordinates

S	0.98961	-0.54889	0.71693
C	0.11589	0.50864	-0.37971
S	-1.50032	1.08389	0.02100
C	-2.63318	-0.38680	-0.25933
O	-1.84376	2.03317	-1.03070
O	-1.59387	1.40104	1.43619
F	-3.89635	0.00909	-0.15243
F	-2.38237	-1.33622	0.64302
F	-2.42278	-0.88723	-1.48309
C	1.29409	-2.17395	-0.18715
O	0.39955	-0.84664	2.00483
O	2.34641	0.11697	0.65919
F	0.13689	-2.81020	-0.32678
F	2.13909	-2.90390	0.52292
F	1.80908	-1.91167	-1.38751
Al	1.81119	1.84305	-0.23015
H	0.14285	0.11295	-1.39909
C	2.73688	1.89555	-1.93459
C	1.54190	3.18453	1.14058
H	1.13772	2.74329	2.05792
H	0.82649	3.94857	0.81629
H	2.46740	3.70542	1.40536
H	2.73984	0.91942	-2.43194
H	3.78268	2.19832	-1.81781
H	2.27703	2.60958	-2.62580

12.3 Methane

SCF energy:	–40.506446 hartree
Zero-point correction:	+0.044773 hartree
Enthalpy correction:	+0.048590 hartree
Free energy correction:	+0.025110 hartree
Truhlar's Delta G correction:	+0.025110 hartree
Grimme's Delta G correction:	+0.025110 hartree

Cartesian Coordinates

C	0.00000	0.00000	0.00000
H	0.28578	-1.05040	-0.06168
H	-0.68183	0.14250	0.83882
H	-0.49484	0.29695	-0.92507
H	0.89089	0.61095	0.14793

12.4 Transition State TS4

SCF energy:	–2174.130095 hartree
Zero-point correction:	+0.180406 hartree
Enthalpy correction:	+0.205349 hartree
Free energy correction:	+0.124980 hartree
Truhlar's Delta G correction:	+0.130258 hartree
Grimme's Delta G correction:	+0.129835 hartree

Imaginary Frequency: 1231.8 $i$cm^{-1}

Cartesian Coordinates

C	-0.34438	0.01696	0.63019
S	0.49523	0.83930	-0.66623
C	1.32786	2.23165	0.28550
F	2.17073	2.84306	-0.53075
F	1.98764	1.72216	1.32615
F	0.40357	3.08467	0.71776
S	-1.68600	-1.02280	0.16428
H	-0.62154	0.69991	1.43556
H	0.68188	-0.85464	1.07503
C	-3.18572	0.10358	0.02643
F	-3.05883	0.94033	-0.99819
O	-1.97724	-1.86655	1.31627
O	-1.43553	-1.55732	-1.16776
O	-0.24007	1.42783	-1.76758
O	1.64295	-0.05832	-1.03022
F	-3.30898	0.81024	1.15672
F	-4.26588	-0.65298	-0.14270
Al	2.46904	-1.58408	-0.10633
C	1.59631	-1.64383	1.82589
H	1.93260	-0.90644	2.56063
H	2.36808	-2.43536	1.83991
H	0.72568	-2.17191	2.22829
C	4.28978	-0.93832	0.15802
H	4.92342	-1.68140	0.65530
H	4.31691	-0.03401	0.77729
H	4.77822	-0.68966	-0.79024
C	1.87161	-3.14689	-1.09634
H	2.31326	-4.06897	-0.70144
H	2.14450	-3.09081	-2.15545
H	0.78387	-3.26880	-1.05619

12.5 Isomeric Catalyst Species I3

SCF energy:	–2133.712514 hartree
Zero-point correction:	+0.138446 hartree
Enthalpy correction:	+0.160421 hartree
Free energy correction:	+0.088392 hartree
Truhlar's Delta G correction:	+0.091007 hartree
Grimme's Delta G correction:	+0.091309 hartree

Cartesian Coordinates

S	1.45270	0.35893	-0.58784
C	0.19876	-0.39711	-1.39714
S	-1.41257	-0.39688	-0.93610
C	-1.72944	-1.64766	0.42795
O	-2.26472	-0.80124	-2.03849
O	-1.76164	0.86043	-0.17909
F	-1.13559	-1.23216	1.54779
F	-1.23002	-2.82947	0.07031
F	-3.03777	-1.75768	0.63309
C	2.42283	-0.89607	0.41370

O	0.86967	1.18266	0.54010
O	2.44468	0.94126	-1.47428
F	1.62549	-1.52548	1.27323
F	3.39387	-0.27560	1.07573
F	2.94868	-1.78248	-0.43121
Al	-0.68214	2.31541	0.45904
C	-1.21652	2.60025	2.29745
C	-0.42533	3.54708	-1.01745
H	-1.33494	4.11073	-1.24967
H	-0.13300	3.02297	-1.93508
H	0.35942	4.28172	-0.80768
H	-2.22817	3.01275	2.37107
H	-0.55177	3.29247	2.82410
H	-1.21186	1.65746	2.85555
H	0.43852	-0.94906	-2.29639

12.6 Deprotonated I2

SCF energy:	–2133.221101 hartree
Zero-point correction:	+0.126100 hartree
Enthalpy correction:	+0.148034 hartree
Free energy correction:	+0.075223 hartree
Truhlar's Delta G correction:	+0.078850 hartree
Grimme's Delta G correction:	+0.078837 hartree

Cartesian Coordinates

C	0.00199	0.61506	-0.51047
S	-1.57279	0.24667	-0.96074
S	1.30706	-0.35521	-0.78405
Al	1.15631	2.01305	0.34279
C	1.54509	3.62962	-0.70021
C	1.07956	2.11655	2.30396
H	0.68289	4.30771	-0.72142
H	2.38739	4.20391	-0.29537
H	1.78198	3.38981	-1.74294
H	0.79899	1.15689	2.75294
H	2.03358	2.42174	2.75124
H	0.33299	2.85048	2.63333
O	-2.31676	1.47064	-1.28468
O	-1.71291	-0.94851	-1.80329
O	2.39278	0.56603	-0.19415
O	1.60449	-1.00315	-2.06219
C	-2.37499	-0.27006	0.65361
F	-1.78483	-1.36425	1.16227
F	-2.28950	0.71068	1.56765
C	1.47091	-1.79948	0.41319
F	2.69959	-2.33486	0.33713
F	0.57653	-2.75029	0.11587
F	1.26538	-1.39951	1.67461
F	-3.67543	-0.54999	0.46208

12.7 Deprotonated I3

SCF energy:	–2133.223300 hartree

Zero-point correction:	+0.125989 hartree
Enthalpy correction:	+0.147553 hartree
Free energy correction:	+0.076689 hartree
Truhlar's Delta G correction:	+0.078992 hartree
Grimme's Delta G correction:	+0.079344 hartree

Cartesian Coordinates

S	1.37557	0.34304	-0.64934
C	0.26877	-0.50644	-1.48051
S	-1.28314	-0.46703	-1.01130
C	-1.67064	-1.63386	0.41700
O	-2.23465	-0.92159	-2.02751
O	-1.77314	0.82305	-0.30087
F	-1.08175	-1.20865	1.54642
F	-1.22625	-2.87065	0.14843
F	-2.99249	-1.70465	0.64081
C	2.41711	-0.81890	0.39231
O	0.86864	1.23013	0.52654
O	2.40695	1.00773	-1.45410
F	1.66241	-1.58239	1.19336
F	3.27444	-0.12630	1.15799
F	3.12785	-1.62437	-0.40853
Al	-0.73163	2.19406	0.43192
C	-1.32353	2.51369	2.26828
C	-0.54725	3.60712	-0.90943
H	-1.50090	4.09553	-1.14272
H	-0.15867	3.20199	-1.85214
H	0.14650	4.39557	-0.59407
H	-0.70134	3.25814	2.77922
H	-1.28522	1.59681	2.86799
H	-2.35538	2.88201	2.31231

13. Calculated Energies and Geometries for the I2-Catalysed Reaction

13.1 Lactone 7-AlC

SCF energy:	–2478.324824 hartree
Zero-point correction:	+0.244491 hartree
Enthalpy correction:	+0.273730 hartree
Free energy correction:	+0.184780 hartree
Truhlar's Delta G correction:	+0.189659 hartree
Grimme's Delta G correction:	+0.189699 hartree

Cartesian Coordinates

C	-5.15798	0.71762	0.18860
C	-4.25316	0.25026	-0.68712
C	-3.08933	-0.46965	-0.20726
O	-2.80550	-0.50565	1.08184
C	-3.44782	0.48821	1.93528
C	-4.92692	0.59734	1.65513
H	-2.93209	1.43387	1.73825
H	-3.22580	0.15737	2.94872
H	-5.32438	1.46888	2.18380
H	-5.48146	-0.26820	2.04374
C	0.42848	-0.33448	-0.01814
S	-0.02946	1.16050	-0.83770
S	2.04742	-1.01593	-0.23276
C	1.11479	2.54591	-0.28187
O	0.14604	1.05206	-2.27778
F	1.24575	2.50524	1.05295
F	2.31307	2.44886	-0.84654
C	3.09138	-0.42402	1.22257
F	2.36123	-0.46224	2.34634
F	3.52907	0.81795	1.03244
F	4.13287	-1.24128	1.34881
O	-2.37080	-1.12905	-0.98803
F	0.55797	3.70635	-0.62719
O	-1.31008	1.56568	-0.24673
O	2.70444	-0.54368	-1.44161
O	1.88097	-2.44377	0.05927
H	-6.08232	1.16388	-0.16836
H	-4.37752	0.30245	-1.76150
Al	-0.73194	-2.08420	-0.63973
H	0.21672	-0.23144	1.05039
C	-0.21080	-2.62267	-2.43665
H	0.63708	-3.31489	-2.43399
H	0.07754	-1.76038	-3.04880
H	-1.03685	-3.11922	-2.95910
C	-1.06320	-3.19846	0.93156
H	-0.83302	-2.66885	1.86383
H	-0.43566	-4.09544	0.91397
H	-2.10708	-3.52575	1.00343

13.2 Transition State TS4a

SCF energy:	–3347.350326 hartree

Zero-point correction:	+0.503245 hartree
Enthalpy correction:	+0.550898 hartree
Free energy correction:	+0.422375 hartree
Truhlar's Delta G correction:	+0.431800 hartree
Grimme's Delta G correction:	+0.431355 hartree
Imaginary Frequency:	318.0 *i*cm^{-1}

Cartesian Coordinates

C	-3.46996	1.61409	1.56928
C	-3.51077	0.28868	1.07157
C	-4.22084	-0.17251	-0.01937
O	-4.23048	-1.42098	-0.41731
H	-2.94199	-0.47392	1.58021
O	-2.72612	1.88339	2.67848
C	-4.01658	2.74455	0.98098
C	-2.01140	0.83178	3.32377
H	-2.69549	0.07324	3.72088
H	-1.47891	1.30474	4.14837
H	-1.29586	0.36242	2.64187
C	-2.52266	3.29704	-0.62722
H	-3.97622	3.66764	1.55008
H	-4.82914	2.62669	0.28166
C	-2.10940	2.19531	-1.37180
C	-0.77439	1.76352	-1.35389
O	0.14089	2.33446	-0.55074
C	-0.37774	3.07995	0.57362
C	-1.46873	4.03264	0.14508
H	-3.36506	3.87602	-0.98893
H	-1.87609	4.53284	1.02786
H	-1.04981	4.82236	-0.49560
H	0.48805	3.59396	0.99379
H	-0.74824	2.35088	1.30421
O	-0.32158	0.87469	-2.14553
O	-5.02226	0.64156	-0.70924
C	-5.63154	0.12169	-1.90030
H	-6.29746	-0.71039	-1.66670
H	-4.86736	-0.20842	-2.60888
H	-6.19564	0.95490	-2.31471
Si	-3.03647	-2.69406	-0.40071
C	-1.79228	-2.18291	-1.67304
H	-1.39504	-1.18581	-1.46116
H	-2.23791	-2.16386	-2.67283
H	-0.94022	-2.86872	-1.69674
C	-2.35188	-2.93326	1.31501
H	-1.79940	-3.87843	1.34738
H	-3.14761	-2.99127	2.06440
H	-1.64972	-2.14722	1.60306
C	-4.08128	-4.14016	-0.92302
H	-4.85513	-4.36626	-0.18407
H	-4.57520	-3.94407	-1.87910
H	-3.46814	-5.03784	-1.04617
Al	1.44522	0.33896	-2.43503
H	-2.76544	1.69920	-2.07773
C	2.13773	-0.05599	-0.40149
C	1.30787	-1.35295	-3.40591
S	1.03453	-1.00455	0.58021

S	3.79077	-0.69424	-0.61681
H	2.17858	0.95995	0.00566
C	4.83289	0.55632	0.32147
F	6.11424	0.21802	0.20931
F	4.49392	0.57798	1.61498
F	4.64948	1.77918	-0.18888
C	1.57055	-0.89034	2.38058
F	1.64294	0.40214	2.74035
F	2.73809	-1.47480	2.61379
F	0.62679	-1.48600	3.12168
O	1.08430	-2.41845	0.23465
O	-0.24258	-0.28371	0.62111
O	4.16265	-0.48224	-2.01487
O	4.00985	-1.97755	0.03551
C	2.39984	1.95540	-3.00457
H	2.14302	-1.48267	-4.10296
H	0.37896	-1.42138	-3.98440
H	1.32591	-2.21790	-2.73125
H	1.78746	2.54968	-3.69448
H	2.64240	2.61361	-2.16148
H	3.34125	1.72577	-3.51351

13.3 Intermediate (*E*)-13-AlC

SCF energy:	–3347.381054 hartree
Zero-point correction:	+0.507408 hartree
Enthalpy correction:	+0.554368 hartree
Free energy correction:	+0.429270 hartree
Truhlar's Delta G correction:	+0.436689 hartree
Grimme's Delta G correction:	+0.436838 hartree

Cartesian Coordinates

C	-3.30919	1.65915	1.44634
C	-3.46044	0.33353	1.09692
C	-4.14166	-0.17276	-0.02804
O	-4.10332	-1.41096	-0.37721
H	-2.97592	-0.42762	1.69055
O	-2.65548	2.01303	2.55181
C	-3.69012	2.83642	0.63955
C	-2.04808	1.00261	3.37133
H	-2.80986	0.33445	3.78362
H	-1.55100	1.54226	4.17441
H	-1.31892	0.43520	2.78546
C	-2.59301	3.25891	-0.41957
H	-3.85349	3.68300	1.31270
H	-4.61676	2.63927	0.10510
C	-2.14843	2.15816	-1.32316
C	-0.85635	1.74801	-1.42894
O	0.15700	2.30216	-0.68506
C	-0.26705	2.96326	0.50909
C	-1.38269	3.94402	0.22396
H	-3.12915	4.02269	-1.00090
H	-1.66157	4.45934	1.14988
H	-0.99984	4.70657	-0.46359
H	0.62408	3.46339	0.89845

H	-0.57013	2.19192	1.22978
O	-0.42702	0.81888	-2.23627
O	-4.94029	0.60245	-0.73132
C	-5.50770	0.05830	-1.94183
H	-6.16899	-0.77619	-1.70800
H	-4.71022	-0.27085	-2.61089
H	-6.06387	0.88170	-2.38257
Si	-2.91473	-2.73012	-0.29471
C	-1.70450	-2.27405	-1.61127
H	-1.34268	-1.24649	-1.49717
H	-2.16344	-2.35951	-2.60134
H	-0.82877	-2.92852	-1.58588
C	-2.27219	-2.87276	1.44256
H	-1.67613	-3.78802	1.52022
H	-3.08823	-2.94947	2.16797
H	-1.61487	-2.04736	1.72157
C	-4.01372	-4.16024	-0.73545
H	-4.78952	-4.32348	0.01765
H	-4.50383	-3.99931	-1.69975
H	-3.43034	-5.08243	-0.81328
Al	1.30400	0.30560	-2.49295
H	-2.86360	1.68313	-1.98623
C	2.06008	-0.06826	-0.43996
C	1.24973	-1.42740	-3.41403
S	0.98135	-0.96889	0.59922
S	3.71065	-0.69967	-0.63705
H	2.08559	0.97408	-0.10703
C	4.74461	0.54533	0.31978
F	6.02582	0.19056	0.24541
F	4.37282	0.58013	1.60513
F	4.59463	1.76933	-0.19823
C	1.55506	-0.82290	2.38840
F	1.69235	0.47433	2.70963
F	2.69196	-1.45999	2.63296
F	0.59200	-1.34836	3.16475
O	1.00199	-2.39585	0.30088
O	-0.29211	-0.23995	0.66480
O	4.11969	-0.49593	-2.02403
O	3.91306	-1.98138	0.02685
C	2.36020	1.85611	-3.08034
H	2.07357	-1.52729	-4.13002
H	0.31290	-1.57429	-3.96527
H	1.34128	-2.26980	-2.71624
H	1.73613	2.55686	-3.64964
H	2.77189	2.42600	-2.23843
H	3.20630	1.57039	-3.71409

13.4 Transition State TS4b

SCF energy:	–3347.363118 hartree
Zero-point correction:	+0.506342 hartree
Enthalpy correction:	+0.552695 hartree
Free energy correction:	+0.426328 hartree
Truhlar's Delta G correction:	+0.436988 hartree
Grimme's Delta G correction:	+0.435648 hartree

Imaginary Frequency: 352.2 $i$cm^{-1}

Cartesian Coordinates

C	5.35366	-0.10379	-0.71568
C	4.36426	-1.02563	-0.82050
C	3.13454	-0.86289	-0.05719
C	2.80364	1.18440	-0.86685
C	3.91683	1.96426	-0.22169
C	5.20190	1.13180	0.09830
H	5.16249	0.84086	1.15356
H	6.09221	1.75160	-0.03087
O	6.52938	-0.16301	-1.36959
C	6.76469	-1.31054	-2.18039
H	7.77395	-1.20099	-2.57228
H	6.04875	-1.35411	-3.00852
H	6.69029	-2.22637	-1.58408
O	2.03385	-1.54385	-0.38134
C	1.46126	1.49106	-0.61257
O	1.07187	2.36590	0.32339
C	2.06787	3.29845	0.80354
C	3.39180	2.62319	1.05308
H	1.63225	3.71848	1.71175
H	2.15161	4.10053	0.05877
H	3.26294	1.85643	1.82805
H	4.10799	3.35565	1.44126
H	4.23512	2.75663	-0.91584
O	0.50859	0.88127	-1.21516
C	1.80852	-1.88298	-1.75460
Al	-1.14264	1.44986	-1.82807
C	-2.50243	0.18508	-0.75471
C	-1.22744	0.88298	-3.70647
C	-1.44165	3.34743	-1.43037
S	-1.99567	-1.51742	-0.80982
H	-3.41619	0.24741	-1.35946
S	-2.93357	0.71597	0.90898
O	-2.01054	1.73568	1.39131
O	-1.00825	-1.80170	0.22561
O	-3.30303	-0.41492	1.76008
O	-1.72840	-1.83274	-2.21203
C	-3.51484	-2.59952	-0.39299
F	-3.44459	-3.08495	0.83853
F	-3.54246	-3.61992	-1.25452
F	-4.64219	-1.89094	-0.53518
C	-4.54400	1.62746	0.57037
F	-5.47938	0.78168	0.12799
F	-4.95563	2.18190	1.70849
F	-4.35999	2.58123	-0.34700
H	-0.83347	1.66200	-4.37157
H	-2.25032	0.67134	-4.04153
H	-0.65003	-0.03012	-3.88660
H	-1.53512	3.57247	-0.36467
H	-2.32457	3.74968	-1.94050
H	-0.58126	3.91860	-1.80684
H	4.40322	-1.84320	-1.53060
H	2.96047	0.82780	-1.87948
O	3.23595	-0.64109	1.23955
H	2.43779	-2.72903	-2.04588

H	0.75701	-2.15720	-1.82028
H	2.00016	-1.02017	-2.39782
Si	2.11237	-1.07531	2.50494
C	3.16413	-0.67199	3.98966
C	1.73765	-2.89256	2.37039
C	0.60548	-0.00678	2.37936
H	1.32241	-3.25823	3.31517
H	2.63767	-3.47681	2.15682
H	1.00402	-3.08429	1.58425
H	0.79229	1.02981	2.67251
H	-0.19371	-0.39783	3.01811
H	0.21466	-0.00297	1.35971
H	4.06929	-1.28512	4.02126
H	2.61248	-0.84413	4.91876
H	3.47209	0.37855	3.98275

13.5 Cycloadduct *endo*-I2-AlC

SCF energy:	–3347.411341 hartree
Zero-point correction:	+0.509089 hartree
Enthalpy correction:	+0.555347 hartree
Free energy correction:	+0.430298 hartree
Truhlar's Delta G correction:	+0.439664 hartree
Grimme's Delta G correction:	+0.438760 hartree

Cartesian Coordinates

C	4.28343	-2.12653	-0.97567
C	4.32864	-1.00737	-0.23315
C	3.11547	-0.23929	0.18376
C	1.78095	-0.96695	-0.17893
C	1.84632	-1.77126	-1.47977
C	3.01289	-2.75001	-1.45189
H	3.17783	-3.17417	-2.44835
H	2.77329	-3.60253	-0.80164
H	5.26746	-0.57262	0.09616
O	5.36115	-2.84690	-1.37264
C	6.63306	-2.39170	-0.93181
H	6.84357	-1.38601	-1.31406
H	7.36178	-3.09650	-1.32858
H	6.68558	-2.37813	0.16329
O	3.07916	1.01848	-0.43964
O	3.15638	0.05596	1.58231
C	0.72682	0.09015	-0.22755
H	1.51720	-1.63234	0.65042
O	0.26774	0.57407	-1.34459
C	0.60448	0.01383	-2.65468
C	1.87358	-0.79927	-2.65438
H	0.64568	0.88819	-3.30572
H	-0.27053	-0.58545	-2.92371
H	2.75388	-0.14985	-2.60118
H	1.91888	-1.34257	-3.60407
H	0.91826	-2.35256	-1.54913
O	0.27863	0.57326	0.83713
C	3.27222	-1.04167	2.46509
H	4.12043	-1.68895	2.20664

H	3.42782	-0.61978	3.45873
H	2.35540	-1.64843	2.48903
C	5.46526	2.23894	0.75395
H	5.89719	3.18783	1.08876
H	5.53245	1.53309	1.58754
H	6.09147	1.86463	-0.06260
C	-2.76506	0.31509	0.48255
C	2.62709	3.11406	1.60068
H	1.58289	3.20827	1.28521
H	2.65256	2.44943	2.46755
H	2.96120	4.10884	1.91489
Si	3.69107	2.47087	0.20742
C	-1.39893	2.01903	2.93438
H	-0.73823	2.82550	3.27430
H	-1.14679	1.13378	3.52808
H	-2.41754	2.31298	3.21470
C	-1.00732	3.19711	-0.27303
H	0.02584	3.56898	-0.26042
H	-1.66002	4.04491	-0.04121
H	-1.22057	2.89792	-1.30383
S	-2.42201	-1.44483	0.44043
O	-1.20401	-1.74959	-0.31237
O	-3.63478	-2.22286	0.20267
C	-2.01043	-1.78841	2.25120
F	-2.88889	-1.17742	3.05133
F	-0.77910	-1.38784	2.56124
F	-2.09456	-3.10582	2.42640
S	-3.54221	0.90646	-1.00656
O	-2.76135	0.52954	-2.18007
C	-5.22125	0.04889	-1.21703
F	-5.11523	-1.09668	-1.87752
F	-5.99765	0.87804	-1.91871
O	-3.92244	2.28674	-0.71915
F	-5.78304	-0.16094	-0.02225
H	-3.52369	0.43399	1.26664
C	3.56096	3.59035	-1.28104
H	3.84453	4.62000	-1.04265
H	2.53104	3.61075	-1.65340
H	4.20162	3.24682	-2.09872
Al	-1.21268	1.73480	1.00860

14. Calculated Energies and Geometries for the I3-Catalysed Reaction

14.1 Lactone 7-AlO

SCF energy:	–2478.343805 hartree
Zero-point correction:	+0.243763 hartree
Enthalpy correction:	+0.273179 hartree
Free energy correction:	+0.183306 hartree
Truhlar's Delta G correction:	+0.189189 hartree
Grimme's Delta G correction:	+0.188841 hartree

Cartesian Coordinates

C	3.95476	-1.05344	-1.44591
C	3.49029	0.19318	-1.63566
C	2.74306	0.84081	-0.57686
O	2.76633	0.37740	0.65221
C	3.70357	-0.69812	0.97493
C	3.80128	-1.72262	-0.12824
H	3.31540	-1.11464	1.90250
H	4.66798	-0.21344	1.16089
H	2.90877	-2.36368	-0.15143
H	4.64613	-2.38598	0.07933
C	-1.02832	-0.43921	1.24476
S	-1.67910	0.95492	0.61923
S	0.03507	-1.49845	0.46538
C	-2.95866	0.62760	-0.71410
O	-2.39855	1.68946	1.65241
F	-3.52232	1.77915	-1.08007
F	-2.39775	0.05393	-1.77682
C	-0.99945	-2.97168	-0.05195
F	-1.55729	-3.54253	1.02223
F	-0.21420	-3.86898	-0.66007
F	-1.96953	-2.60655	-0.89509
O	2.03295	1.84381	-0.82010
F	-3.89631	-0.18324	-0.21853
O	-0.70271	1.71172	-0.25800
O	0.95469	-2.09866	1.43621
O	0.52741	-0.94734	-0.80036
H	4.39827	-1.60115	-2.27328
H	3.52403	0.70589	-2.58872
Al	0.76592	2.83541	0.18028
H	-1.41958	-0.73019	2.21105
C	0.53010	4.45057	-0.87518
H	-0.31618	5.05290	-0.52751
H	0.34306	4.21357	-1.92860
H	1.41251	5.09975	-0.84580
C	1.09098	2.73006	2.09786
H	0.40160	3.37188	2.65811
H	0.96939	1.71151	2.48367
H	2.10610	3.04989	2.36060

14.2 Transition State 5a

SCF energy:	–3347.362159 hartree

Zero-point correction:	+0.503628 hartree
Enthalpy correction:	+0.551059 hartree
Free energy correction:	+0.423002 hartree
Truhlar's Delta G correction:	+0.433394 hartree
Grimme's Delta G correction:	+0.432421 hartree
Imaginary Frequency:	269.3 $i$cm^{-1}

Cartesian Coordinates

C	-2.88494	0.23019	2.37981
C	-1.94727	0.92492	1.57122
C	-2.18157	1.61835	0.40361
O	-1.22101	2.16283	-0.30769
O	-2.43840	-0.40335	3.49716
C	-4.21710	-0.01689	2.09109
C	-1.11756	-0.15275	3.97432
H	-1.04554	-0.69003	4.91864
H	-0.96154	0.91762	4.14826
H	-0.35281	-0.52338	3.28612
C	-4.02109	-1.76248	0.58244
H	-4.72177	0.60802	1.37074
H	-4.81675	-0.50189	2.85431
C	-2.94598	-1.51516	-0.25453
C	-3.12773	-0.92688	-1.51419
O	-4.33866	-0.59129	-1.97795
C	-5.45460	-0.58639	-1.05185
C	-5.38877	-1.68727	-0.01874
H	-3.87462	-2.40819	1.44257
H	-6.16354	-1.51201	0.73316
H	-5.61539	-2.66028	-0.47760
H	-6.33714	-0.66646	-1.68758
H	-5.45967	0.39861	-0.57176
O	-2.19582	-0.66290	-2.34494
O	-3.41778	1.76181	-0.09257
C	-3.56590	2.54608	-1.28934
H	-3.24850	3.57664	-1.11655
H	-4.63071	2.51608	-1.51504
H	-2.99417	2.11026	-2.11074
Si	0.03423	3.29838	0.08606
C	1.36726	2.87645	-1.12823
H	1.65719	1.82383	-1.04833
H	1.04175	3.05366	-2.15678
H	2.26288	3.47896	-0.95174
C	-0.74696	4.96050	-0.23651
H	-1.61357	5.12491	0.41252
H	-1.07815	5.05367	-1.27513
H	-0.03776	5.77123	-0.04258
C	0.54433	3.14305	1.86650
H	1.27057	3.93256	2.08893
H	-0.30052	3.27584	2.54929
H	1.02194	2.18421	2.08176
Al	-0.41396	-1.05903	-2.54437
H	-1.92999	-1.71210	0.05221
C	2.66697	-1.03128	-0.67119
C	-0.21092	-2.95559	-2.95637
S	4.23177	-0.89139	0.00165
S	1.28253	-0.96090	0.22896

C	0.85679	-2.60142	1.04310
F	0.78887	-3.56688	0.12462
F	-0.33693	-2.50046	1.65197
F	1.77654	-2.91422	1.94918
C	4.63886	0.93006	-0.13048
F	5.89681	1.12966	0.27643
F	3.81896	1.65692	0.63880
F	4.52552	1.35249	-1.39640
O	5.17773	-1.52085	-0.91677
O	4.25097	-1.13803	1.44076
O	0.10080	-0.85855	-0.70658
O	1.30112	-0.03979	1.35950
C	0.37736	0.41059	-3.54992
H	-0.79927	-3.58435	-2.27743
H	0.82587	-3.30609	-2.88910
H	-0.55017	-3.18046	-3.97402
H	1.44311	0.55771	-3.33668
H	0.28750	0.26090	-4.63181
H	-0.12665	1.35561	-3.31521
H	-0.89911	0.86545	1.82950
H	2.59338	-1.41543	-1.68112

14.3 Intermediate (*E*)-13-AlO

SCF energy:	–3347.397651 hartree
Zero-point correction:	+0.506993 hartree
Enthalpy correction:	+0.554303 hartree
Free energy correction:	+0.426713 hartree
Truhlar's Delta G correction:	+0.436309 hartree
Grimme's Delta G correction:	+0.435645 hartree

Cartesian Coordinates

C	-2.43772	2.94439	0.56707
C	-3.10961	1.77043	0.84149
C	-3.54639	0.81055	-0.09794
O	-3.96455	-0.35618	0.24443
O	-2.06796	3.77339	1.53276
C	-1.89225	3.37655	-0.74501
C	-2.32889	3.44498	2.90341
H	-3.40326	3.34337	3.07936
H	-1.93256	4.27752	3.47909
H	-1.80459	2.52367	3.17374
C	-0.70450	2.45248	-1.15420
H	-1.53982	4.40661	-0.63219
H	-2.66250	3.34882	-1.52016
C	0.25119	2.17576	-0.03616
C	1.58140	2.44537	-0.10174
O	2.12580	3.22562	-1.08165
C	1.16181	3.94702	-1.85803
C	0.05844	3.03232	-2.34549
H	-1.15657	1.49930	-1.46468
H	-0.60795	3.57718	-3.02411
H	0.50890	2.21497	-2.92132
H	1.72735	4.39560	-2.67695
H	0.75129	4.75589	-1.23333

O	2.46309	1.97872	0.74275
O	-3.60749	1.12412	-1.36823
C	-3.95834	0.08067	-2.31067
H	-5.00577	-0.19291	-2.17965
H	-3.30873	-0.78144	-2.15747
H	-3.79579	0.52853	-3.28808
Si	-3.54192	-1.40444	1.61368
C	-4.93632	-1.12895	2.81498
H	-5.90780	-1.34516	2.36238
H	-4.82462	-1.78436	3.68427
H	-4.95988	-0.09990	3.18790
C	-3.57045	-3.06899	0.80929
H	-2.75401	-3.16957	0.08931
H	-4.51709	-3.23795	0.28854
H	-3.45295	-3.86075	1.55513
C	-1.88945	-0.90113	2.30840
H	-1.38982	-1.79123	2.70579
H	-1.97452	-0.18191	3.12817
H	-1.23076	-0.48333	1.54176
Al	4.09308	1.23630	0.55674
H	-0.07158	1.55747	0.79852
C	1.15900	-0.96804	-0.51901
C	5.06408	1.73738	-1.06361
S	-0.19979	-1.81516	-1.02935
S	2.52277	-1.65146	0.14030
C	2.24681	-1.86067	1.98292
F	3.38066	-2.22727	2.58480
F	1.31391	-2.79227	2.20106
F	1.82944	-0.70249	2.51026
C	-0.06446	-1.91493	-2.89127
F	1.01896	-2.59400	-3.26604
F	-0.00452	-0.67867	-3.41337
F	-1.15294	-2.52571	-3.38653
O	-1.38583	-0.95181	-0.87712
O	-0.24436	-3.20991	-0.59378
O	3.60530	-0.61310	0.15085
O	2.87594	-2.98168	-0.32781
C	4.90046	1.11295	2.33403
H	5.34089	2.79802	-1.06872
H	4.45174	1.56871	-1.95725
H	5.98775	1.16054	-1.18984
H	5.38868	2.04779	2.63393
H	4.15434	0.88513	3.10436
H	5.66439	0.32812	2.38621
H	-3.29769	1.49779	1.87162
H	1.05412	0.11122	-0.44055

14.4 Transition State TS5b

SCF energy:	−3347.363118 hartree
Zero-point correction:	+0.506342 hartree
Enthalpy correction:	+0.552695 hartree
Free energy correction:	+0.426328 hartree
Truhlar's Delta G correction:	+0.436988 hartree
Grimme's Delta G correction:	+0.435648 hartree

Imaginary Frequency: 352.2 $i$cm^{-1}

Cartesian Coordinates

C	5.35366	-0.10379	-0.71568
C	4.36426	-1.02563	-0.82050
C	3.13454	-0.86289	-0.05719
C	2.80364	1.18440	-0.86685
C	3.91683	1.96426	-0.22169
C	5.20190	1.13180	0.09830
H	5.16249	0.84086	1.15356
H	6.09221	1.75160	-0.03087
O	6.52938	-0.16301	-1.36959
C	6.76469	-1.31054	-2.18039
H	7.77395	-1.20099	-2.57228
H	6.04875	-1.35411	-3.00852
H	6.69029	-2.22637	-1.58408
O	2.03385	-1.54385	-0.38134
C	1.46126	1.49106	-0.61257
O	1.07187	2.36590	0.32339
C	2.06787	3.29845	0.80354
C	3.39180	2.62319	1.05308
H	1.63225	3.71848	1.71175
H	2.15161	4.10053	0.05877
H	3.26294	1.85643	1.82805
H	4.10799	3.35565	1.44126
H	4.23512	2.75663	-0.91584
O	0.50859	0.88127	-1.21516
C	1.80852	-1.88298	-1.75460
Al	-1.14264	1.44986	-1.82807
C	-2.50243	0.18508	-0.75471
C	-1.22744	0.88298	-3.70647
C	-1.44165	3.34743	-1.43037
S	-1.99567	-1.51742	-0.80982
H	-3.41619	0.24741	-1.35946
S	-2.93357	0.71597	0.90898
O	-2.01054	1.73568	1.39131
O	-1.00825	-1.80170	0.22561
O	-3.30303	-0.41492	1.76008
O	-1.72840	-1.83274	-2.21203
C	-3.51484	-2.59952	-0.39299
F	-3.44459	-3.08495	0.83853
F	-3.54246	-3.61992	-1.25452
F	-4.64219	-1.89094	-0.53518
C	-4.54400	1.62746	0.57037
F	-5.47938	0.78168	0.12799
F	-4.95563	2.18190	1.70849
F	-4.35999	2.58123	-0.34700
H	-0.83347	1.66200	-4.37157
H	-2.25032	0.67134	-4.04153
H	-0.65003	-0.03012	-3.88660
H	-1.53512	3.57247	-0.36467
H	-2.32457	3.74968	-1.94050
H	-0.58126	3.91860	-1.80684
H	4.40322	-1.84320	-1.53060
H	2.96047	0.82780	-1.87948
O	3.23595	-0.64109	1.23955
H	2.43779	-2.72903	-2.04588

H	0.75701	-2.15720	-1.82028
H	2.00016	-1.02017	-2.39782
Si	2.11237	-1.07531	2.50494
C	3.16413	-0.67199	3.98966
C	1.73765	-2.89256	2.37039
C	0.60548	-0.00678	2.37936
H	1.32241	-3.25823	3.31517
H	2.63767	-3.47681	2.15682
H	1.00402	-3.08429	1.58425
H	0.79229	1.02981	2.67251
H	-0.19371	-0.39783	3.01811
H	0.21466	-0.00297	1.35971
H	4.06929	-1.28512	4.02126
H	2.61248	-0.84413	4.91876
H	3.47209	0.37855	3.98275

14.5 Cycloadduct *endo*-I1-AlO

SCF energy:	–3347.432093 hartree
Zero-point correction:	+0.508432 hartree
Enthalpy correction:	+0.554888 hartree
Free energy correction:	+0.430474 hartree
Truhlar's Delta G correction:	+0.438728 hartree
Grimme's Delta G correction:	+0.438474 hartree

Cartesian Coordinates

C	-2.09134	2.76768	0.88997
C	-2.71593	1.58382	1.01386
C	-2.29213	0.34654	0.28980
C	-0.93750	0.53182	-0.46880
C	-0.76127	1.92846	-1.06557
C	-0.89767	2.98765	0.01940
H	-0.96482	3.98634	-0.42600
H	0.00447	3.00100	0.64687
H	-3.59204	1.46337	1.64284
O	-2.44431	3.90761	1.53321
C	-3.53276	3.81976	2.44251
H	-4.45547	3.53420	1.92362
H	-3.64676	4.81186	2.87603
H	-3.32367	3.08921	3.23308
O	-3.25045	-0.03911	-0.65868
O	-2.17542	-0.77267	1.17779
C	-0.89515	-0.53997	-1.51193
H	-0.12835	0.32833	0.23848
O	-1.20877	-0.30155	-2.75463
C	-1.46226	1.04509	-3.27153
C	-1.74240	2.08967	-2.21971
H	-2.29152	0.90460	-3.96655
H	-0.55996	1.28066	-3.84449
H	-2.77388	2.01432	-1.86173
H	-1.62610	3.07088	-2.69214
H	0.25662	1.98463	-1.47090
O	-0.64080	-1.73081	-1.24302
C	-1.53205	-0.51178	2.41475
H	-0.61904	0.07969	2.28180

H	-2.19240	0.01792	3.11308
H	-1.25765	-1.48160	2.83533
C	-5.59859	-0.74830	-1.90532
H	-6.48464	-1.38901	-1.94712
H	-5.93615	0.29221	-1.93522
H	-5.01534	-0.93379	-2.81349
C	3.65269	-0.76602	0.01374
C	-5.46918	-0.62093	1.18304
H	-4.82979	-0.74712	2.06249
H	-5.81848	0.41661	1.15825
H	-6.34890	-1.25770	1.32402
Si	-4.56417	-1.08651	-0.38736
C	-0.62005	-3.91493	0.96724
H	-1.34841	-3.32705	1.53527
H	-1.18592	-4.67517	0.41654
H	0.00656	-4.44976	1.69084
C	1.85220	-3.52566	-1.38817
H	2.73989	-3.84558	-0.82894
H	2.19015	-2.80314	-2.13871
H	1.49256	-4.40621	-1.93335
S	3.17808	-0.00784	-1.42536
O	1.72305	-0.03152	-1.60470
O	4.08924	-0.39308	-2.49778
C	3.54089	1.81740	-1.21112
F	4.82068	2.00852	-0.87943
F	2.76541	2.34709	-0.25344
F	3.28796	2.45662	-2.35940
O	3.36791	-2.29091	2.06786
C	2.41369	0.11676	2.52527
F	3.58846	0.62328	2.89941
F	1.77898	-0.36402	3.59571
O	1.25462	-1.45919	0.89194
F	1.67089	1.09279	1.98601
C	-4.00429	-2.86429	-0.35392
H	-3.43551	-3.08558	0.55255
H	-3.35895	-3.08857	-1.20966
H	-4.86051	-3.54582	-0.39724
S	2.70601	-1.25786	1.28156
Al	0.46451	-2.81761	-0.21870
H	4.70497	-0.99387	0.12459

15.References

[1] T. Fischer, J. Pietruszka, *Adv. Synth. Catal.* **2012**, 2521-2530; 'Alcohol Dehydrogenase-Catalyzed Synthesis of Enantiomerically Pure δ-Lactones as Versatile Intermediates for Natural Product Synthesis'.

[2] D. Böse, P. Niesobski, M. Lübcke, J. Pietruszka, *J. Org. Chem.* **2014**, *79*, 4699-4703; 'A diastereoselective one-pot, three-step cascade toward α-substituted allylboronic esters'.

[3] S. Fiorito, F. Epifano, V. A. Taddeo, S. Genovese, *Tetrahedron Lett.* **2016**, *57*, 2939-2942; 'Ytterbium triflate promoted coupling of phenols and propiolic acids: synthesis of coumarins'.

[4] J. L. Banks, H. S. Beard, Y. Cao, A. E. Cho, W. Damm, R. Farid, A. K. Felts, T. A. Halgren, D. T. Mainz, J. R. Maple, R. Murphy, D. M. Philipp, M. P. Repasky, L. Y. Zhang, B. J. Berne, R. A: Friesner, E. Gallicchio, R. M. Levy, Integrated Modeling Program, Applied Chemical Theory (IMPACT). *J. Comput. Chem.* **2005,** *26* (16), 1752–1780.

[5] Schrödinger Release 2017−4; MacroModel, Schrödinger, LLC: New York, NY, 2017.

[6] Y. Zhao, D. G. Truhlar, A new local density functional for main-group thermochemistry, transition metal bonding, thermochemical kinetics, and noncovalent interactions. *J. Chem. Phys.* **2006,** *125* (19), 194101.

[7] S. Grimme, J. Antony, S. Ehrlich, H. Krieg, A consistent and accurate ab initio parametrization of density functional dispersion correction (DFT-D) for the 94 elements H-Pu. *J. Chem. Phys.* **2010,** *132* (15), 154104.

[8] E. Cancès, B. Mennucci, J. Tomasi, A new integral equation formalism for the polarizable continuum model: Theoretical background and applications to isotropic and anisotropic dielectrics. *J. Chem. Phys.* **1997,** *107* (8), 3032–3041.

[9] R. F. Ribeiro, A. V. Marenich, C. J. Cramer, D. G. Truhlar, Use of Solution-Phase Vibrational Frequencies in Continuum Models for the Free Energy of Solvation. *J. Phys. Chem. B* **2011,** *115* (49), 14556–14562.

[10] Y. Zhao, D. G. Truhlar, The M06 suite of density functionals for main group thermochemistry, thermochemical kinetics, noncovalent interactions, excited states, and transition elements: two new functionals and systematic testing of four M06-class functionals and 12 other functionals. *Theor. Chem. Acc.* **2008,** *120* (1), 215–241.

[11] F. Weigend, R. Ahlrichs, Balanced basis sets of split valence, triple zeta valence and quadruple zeta valence quality for H to Rn: Design and assessment of accuracy. *Phys. Chem. Chem. Phys.* **2005,** *7* (18), 3297–3305.

[12] M. J. Frisch, G. W. Trucks, H. B. Schlegel, G. E. Scuseria, M. A. Robb, J. R. Cheeseman, G. Scalmani, V. Barone, B. Mennucci, G. A. Petersson, H. Nakatsuji, M. Caricato, X. Li, H. P. Hratchian, A. F. Izmaylov, J. Bloino, G. Zheng, J. L. Sonnenberg, M. Hada, M. Ehara, K. Toyota, R. Fukuda, J. Hasegawa, M. Ishida, T. Nakajima, Y. Honda, O. Kitao, H. Nakai, T. Vreven, J. A. Montgomery Jr., J. E. Peralta, F. Ogliaro, M. Bearpark, J. J. Heyd, E. Brothers, K. N. Kudin, V. N. Staroverov, R. Kobayashi, J. Normand, K. Raghavachari, A. Rendell, J. C. Burant, S. S. Iyengar, J. Tomasi, M. Cossi, N. Rega, J. M. Millam, M. Klene, J. E. Knox, J. B. Cross, V. Bakken, C. Adamo, J. Jaramillo, R. Gomperts, R. E. Stratmann, O. Yazyev, A. J. Austin, R. Cammi, C. Pomelli, J. W. Ochterski, R. L. Martin, K. Morokuma, V. G. Zakrzewski, G. A. Voth, P. Salvador, J. J. Dannenberg, S. Dapprich, A. D. Daniels, Ö. Farkas, J. B. Foresman, J. V. Ortiz, J. Cioslowski, D. J. Fox, *Gaussian 09, Revision E.01*, Gaussian, Inc.: Wallingford CT, 2009.
